# Deciphering the Retinal Epigenome during Development, Disease and Reprogramming: Advancements, Challenges and Perspectives

**DOI:** 10.3390/cells11050806

**Published:** 2022-02-25

**Authors:** Cristina Zibetti

**Affiliations:** Department of Ophthalmology, Institute of Clinical Medicine, University of Oslo, Kirkeveien 166, Building 36, 0455 Oslo, Norway; zibettic@gmail.com

**Keywords:** retinal epigenome, next-generation sequencing, transcription factors, retinal progenitor cells, neurogenesis, cell fate, gene regulatory networks, neurodegeneration, reprogramming, stem cells, retina, genome, chromatin, non-coding RNA, enhancer, epigenetics, iPS cells, animal models, single-cell analysis, transcriptomic

## Abstract

Retinal neurogenesis is driven by concerted actions of transcription factors, some of which are expressed in a continuum and across several cell subtypes throughout development. While seemingly redundant, many factors diversify their regulatory outcome on gene expression, by coordinating variations in chromatin landscapes to drive divergent retinal specification programs. Recent studies have furthered the understanding of the epigenetic contribution to the progression of age-related macular degeneration, a leading cause of blindness in the elderly. The knowledge of the epigenomic mechanisms that control the acquisition and stabilization of retinal cell fates and are evoked upon damage, holds the potential for the treatment of retinal degeneration. Herein, this review presents the state-of-the-art approaches to investigate the retinal epigenome during development, disease, and reprogramming. A pipeline is then reviewed to functionally interrogate the epigenetic and transcriptional networks underlying cell fate specification, relying on a truly unbiased screening of open chromatin states. The related work proposes an inferential model to identify gene regulatory networks, features the first footprinting analysis and the first tentative, systematic query of candidate pioneer factors in the retina ever conducted in any model organism, leading to the identification of previously uncharacterized master regulators of retinal cell identity, such as the nuclear factor I, NFI. This pipeline is virtually applicable to the study of genetic programs and candidate pioneer factors in any developmental context. Finally, challenges and limitations intrinsic to the current next-generation sequencing techniques are discussed, as well as recent advances in super-resolution imaging, enabling spatio-temporal resolution of the genome.

## 1. Introduction

The eye, the primary sense organ for vision, is often seen as an extension of the brain, and a model system to study the central nervous system. Despite sharing the same neuroectodermal origin, secreted signalling factors drive the regionalization of the neural plate along the anteroposterior axis, originating the prosencephalon that comprises the telencephalon, the eye, and the diencephalon, followed by the midbrain, the hindbrain, and the spinal cord. Neuroepithelial cells originating from different subdomains can acquire distinct, progressively restricted cell identities, under the combinatorial, dosage-dependent action of several transcription factors (TFs), giving rise to the precursors of the eye field [1,2,3].

Functionally equivalent in its laminar structure [4,5,6,7], the retina displays a similarly stereotypic temporal patterning resulting from cell-intrinsic clocks and extrinsic signals [8,9], similar response to injury, and similar immune responses [10,11,12,13,14,15]. Ultimately, the retina has proved to be an accessible tool to investigate molecular determinants of neurogenesis and neurodegeneration [16]. In fact, several neurodegenerative diseases have ocular manifestation [17,18,19], and signs of visual impairment may enable the non-invasive diagnosis of central nervous system and spinal cord disorders.

Color perception, memory, and object recognition can be monitored and serve as early biomarkers for neurological dysfunction, to assess concussion incurred while playing sport [20], or demyelinating diseases such as multiple sclerosis that attacks the optic nerve resulting in optic neuritis [21,22].

Optical coherence tomography, traditionally adopted to scan retinal thickness in age-related macular degeneration and glaucoma can be used to monitor other neurological conditions, such as multiple sclerosis and Parkinson’s disease [23,24,25,26], for which visual acuity tests can also be leveraged, involving measuring low-contrast letter acuity, the speed of rapid number naming and rapid picture naming [27,28,29]. Combined usage of optical coherence tomography and angiography can be adopted to identify early signs of multiple sclerosis as well as early, pre-symptomatic lesions as Alzheimer’s disease before the onset of cognitive signs, by monitoring the enlargement of the foveal avascular zone [30,31].

Recent evidence suggests that loss of retinal thickness, structural abnormalities, and neovascularization elicit an impaired electrical response to the light by electroretinography and may be early signs of neuropsychiatric disorders, such as schizophrenia, major depressive, and bipolar disorders [32,33,34].

Understanding the molecular mechanisms that underpin retinal neurogenesis and degeneration leading to visual dysfunction holds promise for the early neurological diagnosis and potential treatment of neurodegenerative diseases.

Several model organisms have been leveraged to investigate the retinogenesis, retinal degeneration, and differences in the regenerative capacity of the retina, leading to a compendium of data over genetics, epigenetic master regulators of cell fate identity, and related gene regulatory networks.

This review will present the state-of-the-art approaches to functionally interrogate the retinal epigenome during development, disease, and reprogramming.

I will then describe a pipeline to query the epigenetic and transcriptional networks underlying cell fate specification that is virtually applicable to any developmental context, for the study of genetic regulatory programs and candidate pioneer factors.

Recent advances in super-resolution imaging, enabling spatio-temporal resolution of the genome will be briefly described. Finally, I will discuss challenges and limitations intrinsic to current next-generation sequencing techniques.

## 2. Temporal Patterning in the Retina

Over the course of neurogenesis neural progenitors transition through different states of developmental competence, in which they successively acquire and lose their ability to generate individual cell subtypes, as a result of context-dependent TFs regulatory functions [35,36,37,38]. This process, commonly referred to as temporal patterning, occurs throughout the nervous system in vertebrates and invertebrates, it is largely cell-autonomous and proceeds in a unidirectional and irreversible manner [35,39,40,41,42,43,44,45]. TFs contribute to defining, together with DNA methylation and histone modification marks, the so-called “epigenetic code” or chromatin states and configurations that the DNA is topologically structured into.

While seemingly redundant, TFs diversify their regulatory outcome on gene expression by coordinating variations in chromatin landscapes, driving divergent cell specification programs. Retinal cell identity as well can be defined by chromatin states, transcriptional profiles, TFs recruitment, and clustering of epigenetic marks.

High-order chromatin structure is organized across diffuse and dense chromatin compartments [46,47,48,49,50,51,52,53,54] and is defined by long-distance chromatin interactions that bring distal regulatory elements, like the enhancers, into proximity of promoter regions by protruding DNA loops. DNA loops are anchored by the insulator factor CCCTC-binding factor (CTCF) and cohesin proteins occurring across topologically associated domains (TADs). Disruptions of topological chromatin domains are known to cause pathogenic rewiring of gene-enhancer interactions [55].

Ultimately, long-distance chromatin interactions enable distal regulation of promoter regions and underlie transcriptional programs in cell specification pathways [56,57,58,59,60,61].

### 2.1. Molecular Staging and Taxonomic Classification of the Developing Retina

The recent advent of single-cell next-generation sequencing methods has enabled molecular classification, comparative taxonomy, and stratification of molecular heterogeneity within cell populations at an unprecedented scale [62,63,64,65,66,67,68,69,70,71,72,73,74].

Molecular staging of the developing retina has been led by clustering of gene expression [63] and by clustering of coordinately accessible open chromatin followed by identification of relevant TFs and related binding patterns in mice [75] and humans [69]. Single-cell transcriptomics has allowed to further define cellular identity and heterogeneity within populations [65].

Several studies have integrated gene expression profiling by RNA-sequencing (RNA-seq) with in situ hybridization and immunofluorescence, profiling three major epochs in human retinal development and identifying transcriptional determinants of the central-to-peripheral gradient as well as nasal-temporal developmental gradient, where cellular organization and lamination in the cone-only fovea are complete by day 96, whereas the nasal retina and the peripheral retina have a much more prolonged development. The first epoch is characterized mostly by retinal progenitor cells then retinal ganglion cells by human gestational day D50-D60, the second epoch by horizontal and amacrine cells, and the third epoch by photoreceptors, bipolar cells, and Müller glia [63,76].

Comparative studies led by single-cell RNA-seq of the retina across macaque, humans, marmoset, and mice have addressed the heterogeneity of the seven major retinal subtypes, identifying roughly 120 subtypes in mice, 70 cell types in the peripheral retina of primates and 65 in the fovea, while 63 peripheral subtypes and 40 foveal retinal subtypes were found in human [65]. Higher orthologous mouse-to-primate conservation was found for photoreceptors, amacrine, and bipolar cells related transcriptomes compared to retinal ganglion cells. Surprisingly, lower retinal ganglion cells heterogeneity was found in primates compared to mice, although selective expression of retinal ganglion cell-specific TFs was retained across species [65]. Transcriptome analysis identified 53 cell subtypes in the peripheral human adult retina, 41 in the fovea [72], and 40 cell types in retinal organoids obtained from human fibroblasts. Stages of the human developing retina have been cross-referenced against and correlated with those in developing organoids by comparative open chromatin profiling [69].

### 2.2. Temporal Patterning from an Epigenomic Perspective

Several methods have been developed to profile open chromatin regions, nucleosome phasing and TFs binding such as the assay for transposase accessible chromatin (ATAC-seq) and footprinting [77,78,79], coupled with chromatin immunoprecipitation sequencing for any TF of interest (ChIP-seq), transcriptional profiling (RNA-seq), and chromosome conformation capture methods (3C and derivative high-throughput methods such as HiC), or the chromatin interaction analysis by paired-end tag sequencing (ChiA-PET) for the identification of long-distance, intra, and inter-chromosomal chromatin interactions [50,80,81,82].

Extensive work has led to the profiling of epigenetic marks during retinal development, tumorigenesis, and reprogramming, by comparing the human fetal retina at different stages of development with human and murine retinoblastoma and murine rod-derived iPSCs [83].

Chromatin states throughout retinal development can be defined by clustering of histone acetylation and methylation marks by hidden Markov models (HMM) and paired variations in gene expression [83,84]. Post-translational modifications of histones on corresponding promoter regions are predictive of gene expression and can be profiled by chromatin immunoprecipitation sequencing (ChIP-seq). ChIP-seq can identify genome-wide binding for the insulator proteins CTCF, BRD4, mediator, RNA-Pol-II, the histone marks H3K4-me1,2,3, H3K36me3, and enhancers decorated by H3K27ac and/or H3K4me1 [85,86].

As retinogenesis proceeds, de-repression of cell type-specific genes occurs and it is paralleled by progressive silencing of cell-cycle related and retinal progenitor cells related genes, such as *Ascl1* [87], decorated by H3K27me3 and H3K9me3 repressive marks. HMM states and stage-specific super-enhancers marked by H3K27ac histone acetylation within TADs [61], are evolutionarily conserved between mouse and human, whereas DNA methylation regions are less conserved. Whole-genome bisulfite sequencing indicates DNA methylation and gene expression are inversely correlated but account only for a small fraction of the total number of developmentally regulated genes.

### 2.3. A Fine Balance between Reprogramming Capacity and Epigenetic Memory

All vertebrate retinas display a common organization and share basic cell types, although the retina has adapted to suit each species’ ecological niche in response to different levels of light exposure, diversifying neural circuitry, cell subtypes, [88,89], cellular organization functional to light scattering and visual acuity [90,91,92], optical transparency [93,94] as well as subnuclear compartmentalization properties [95].

Pioneering studies on nuclear architecture led by immunocytochemistry [95], optics, and biophotonics [96] have shown that rod photoreceptors in nocturnal animals have an inverted nuclear structure, compared to diurnal animals, with specific refractive properties that enable better light propagation throughout the outer nuclear layer. In nocturnal rods, transcriptionally active euchromatin is situated at the periphery of the nucleus, while transcriptionally inaccessible and denser heterochromatin is located at its center, with an interposed layer of facultative heterochromatin. The rods of diurnal retinas, instead, possess the conventional architecture found in nearly all eukaryotic cells, with most heterochromatin situated at the nuclear periphery and active euchromatin residing toward the nuclear interior.

Later studies have relied on these foundational findings, in the attempt to elucidate differences in the nuclear architecture and cell type-specific reprogramming properties of the vertebrate retina [83,97,98]. Five different cell types in the murine retina and derivative iPSCs lines were produced in mosaic co-cultures with post-natal retinal pellets; the iPSCs lines were compared at two developmental stages for their ability to regenerate retinal organoids and to display cellular pliancy [99], that is, predisposition to undergo reprogramming by the acquisition of epigenetically active states. An inverse correlation was noticed between epigenetic memory and regenerative capacity across all derived cell types [97].

The regenerative capacity of retinal-derived iPSCs reminisces the progressive expansion of open chromatin states occurring during cancer, which is accompanied by the induction of proto-oncogenes [100,101], and it is inversely related to the epigenetic memory.

Epigenetic memory is cell-type specific; it is accompanied by persistent retention of repressive histone methylation marks and DNA methylation, and it partially depends on the sequestration of reprogramming genes to nuclear domains of facultative heterochromatin.

Conversely, full reprogramming capacity entails silencing of cell type-specific genes, resetting of poised enhancers and re-organization of heterochromatin to a pluripotent state.

In rods-derived iPSCs, that retain epigenetic memory of their cellular origin, pluripotency-related genes are sequestered into compartments of facultative heterochromatin and the overall heterochromatin content is higher compared to that in other cell types.

H3K9me3 marks are more resilient in rods-derived murine iPSCs where genes involved in the neurogenic process are actively repressed. Rods and bipolar-derived iPSCs have a more efficient retinal differentiation and the lowest reprogramming efficiency. In contrast, Müller glia, horizontal and amacrine cells have a lower proportion of nuclear volume sequestered into heterochromatin and are more prone to undergo complete reprogramming. Subsequent studies on purified murine rod photoreceptors have furthered these findings indicating that developmental, cancer, and stress-response genes are localized to facultative heterochromatin domains in rods [102]. Furthermore, integration of HiC analysis with H3K27ac marks and CTCF chromatin immunoprecipitating sequencing (ChIP-seq) have led to the identification of stage-specific super-enhancers, distal regulatory elements brought to the proximity of promoter regions by protruding DNA loops, and long-distance chromatin interactions across multiple TADs [98,102].

Cell competence and timing of neuronal differentiation are driven by several TFs that control the proper ratio of cell types via cell-intrinsic clocks [103,104,105,106,107], gene regulatory networks [108,109], and exposure to extrinsic signals [7,9,110,111,112].

Expression patterns for most of the retinal TFs have been extensively characterized [36,113,114,115,116,117,118,119]. Nonetheless, the specific mechanisms by which TFs coordinate epigenetic changes across retinal development remain elusive, since the direct assessment of binding sites is limited to select cases, such as for CRX, OTX2, and RORB, NFI, NRL, or MEF2D [120,121,122,123,124,125], whereas most of these mechanisms are inferred [69,98,126,127,128,129].

Furthermore, the epigenomic studies may be affected by experimental biases that are intrinsic to cell cultures systems and neoplastic conditions, or due to the spurious contribution of heterogeneous cell types composing the whole retinal tissue [83], where multiple cell types contribute to histone methylation and acetylation marks, enhancers elements, and long-range chromatin interactions [98].

While transcriptional and epigenetic descriptors do not necessarily address the mechanistic aspects of cell fate determination in the retina, the advent of single-cell sequencing has allowed, at least, to resolve such molecular complexity, by deconvolving the cell type specificity of regulatory elements that could not otherwise be discerned from conventional bulk sequencing [98,124].

A systematic census of TFs *cis*- and *trans*-regulatory sites that are necessary and sufficient for the establishment of developmental trajectories is still missing. Nonetheless, the knowledge of the epigenomic mechanisms that drive the acquisition and stabilization of retinal cell fates holds the potential for the treatment of retinal degeneration.

An overwhelming amount of data have begun to accumulate, yet a question remains to be addressed and it is how, exactly, evolutionarily conserved, and divergent TF binding patterns coordinate epigenetic changes across retinal development.

## 3. The Epigenome: A Pandora’s Box

### 3.1. A Truly Unbiased Screening of Open Chromatin States: From Clustering of De Novo Oligonucleotides to the Identification of TFs Binding Patterns in Retinal Cell Fate Specification

Functional interrogation of next-generation sequencing profiles can reveal TFs regulatory networks underlying retinal cell fate specification.

Previous work was led on flow-sorted retinal progenitor cells, purified from transgenic mice by virtue of the pan-retinal reporter VSX2 (*Chx10-GFP-Cre*) [75]. An agnostic screening of open chromatin states in early and late-stage retinal progenitor cells was paralleled by age-matched mRNA expression, inferential analysis of *cis*- and *trans*-acting TFs that control temporal patterning, and footprinting profiling of TFs binding patterns, coupled with the identification of gene regulatory networks.

Interrogation of ATAC-seq and RNA-seq, paired with age-matched ChIP-seq for the top candidate TF identified the LIM-homeodomain TF LHX2 as a central regulator of chromatin accessibility across several stages of retinal development.

Hierarchical clustering of probabilistically assigned position weight matrices (PWM) led to the identification of the most functionally enriched TFs, among which homeobox domain-containing TFs, followed by C2H2-type, GC-rich, and KLF zinc finger proteins, then POU-homeodomain, MADS-box, and CCAAT-binding TFs. Individual motif instances were also found associated with NFY factors and the insulator protein CTCF, involved in chromatin looping and conformation. Among the putative assignments, many motif instances reflected positional variations of the LHX2 consensus [75]. LHX2 was the most represented TF motif by binomial scoring of known PWM both in retinal progenitor cells and stage-specific accessible regions. LHX2 occupancy was ascertained by footprinting analysis at both stages. ChIP-seq was performed for LHX2, integrated with age-matched RNA-seq and ATAC-seq profiles from flow-sorted retinal progenitor cells. The phenotypic readout on chromatin accessibility and transcriptome was re-assessed upon *Lhx2* loss of function (*Chx10-GFP-Cre; Lhx2^fl/fl^*) in early- and late-stage retinal progenitor cells.

### 3.2. Lhx2 Is Required for the Regionalization of the Optic Vesicle through Cell-Autonomous Regulation of Gene Expression and for the Neuro-Retinal Suppression of Alternative Diencephalic Cell Fates

LHX2 regulates the neural differentiation of human embryonic stem cells by inhibition of the BMP-WNT signaling pathway [130] and is required for the transition of early-stage retinal progenitor cells from an immature state, when they give rise to retinal ganglion cells [131], to a later stage, when they generate late-born Müller glia by enhancing the Notch signalling pathway [132,133]. Ultimately, LHX2 maintains the retinal progenitor cells’ proliferative potential and neurogenic competence by preventing the generation of later cell types and an ectopic expression of genes specific to the anterodorsal hypothalamus and thalamic eminence [134].

LHX2 also defines the cortical identity of neuroepithelial progenitors, while repressing the expression of genes enriched within the cortical hem and ectopic hippocampal fields [135,136]. Additional studies indicate LHX2 has a role in radial glial cell populations that give rise to neurons and astrocytes in the hippocampus and neocortex [137,138]. Ablation of *Lhx2* in neocortical radial glial progenitor cells approaching cortical neurogenesis results in a cell cycle exit, premature production of neurons and a loss of the stellate astrocytes [139].

This LIM homeodomain TF has been characterized in a plethora of developmental contexts and it is required for the eye field specification and the morphogenesis of the optic cup through cell autonomous regulation of gene expression [134,140,141]. While *Lhx2* mutants are anophthalmic, display hypoplasia of the neocortex and severe anaemia, neuroretina specific loss of function of *Lhx2* causes severe microphthalmia, loss of expression of a subset of retinal progenitor cell-specific genes, and ectopic expression of hypothalamic genes [134,140,142].

Ocular expression of *Lhx2* has been observed as early as embryonic day e8.5; it can be detected in the retinal neuro-epithelium and retinal pigmented epithelium (RPE) at e10 and ciliary margin by e14, becoming progressively restricted to the inner nuclear layer. In the post-natal retina, the LHX2 TF is expressed in mitotic retinal progenitor cells that co-express VSX2 (CHX10) and Ki67. By post-natal day P7, LHX2 expression becomes restricted to differentiating Müller glia and is preserved in adult Müller glia, a subset of bipolar and starburst amacrine cells [132].

### 3.3. Lhx2 Is Expressed in Neuro-Epithelial, Bipotent Progenitors That Give Rise to Segregated, Neuroretina-like and RPE-like Domains in hiPSCs-Derived Optic Cups and Organoids

Eye development in the embryo arises from the eye field, a centrally organized domain of the neural plate, composed of neuroepithelial cells that express several TFs including PAX6, RX, LHX2, SIX3, SIX6, and it is surrounded by anterior neuroepithelial cells expressing PAX6 and SOX1. In the eye field formation, cell-fate specification of the anterior neuroectoderm into either the neuro-retina or the RPE is regulated critically by two TFs, the visual system homeobox 2 (VSX2) and the microphthalmia-associated TF (MITF), which are initially co-expressed in the bipotent progenitor cells but subsequently become restricted to the neuroretina and RPE, respectively [143,144,145,146].

The expression patterns of neuroepithelial cells can be recapitulated fairly well by the optic cups, where retinal progenitor cells begin to emerge after one week in vitro: the central region of differentiating aggregates downregulates the pluripotency markers OCT4 and SOX1 and induces the neural markers PAX6 and SIX3, followed by LHX2 and RX, and eventually SIX6, mimicking the chronology of expression of the eye field TFs seen in vivo [143,147]. Segregation of human-induced pluripotent stem cells (hiPSCs)-derived optic cups into the neuroretina-like and presumptive RPE domains also occurs upon reciprocal inhibition by the eye field TFs VSX2 and MITF, with MITF preceding the expression of the neuro-retinal marker VSX2 [148]. The Neuroretina-like domain expresses PAX6, LHX2, RX, and VSX2, whereas the peripheral, RPE-like domain expresses MITF and PAX6 and, between the third and the fourth week in vitro, optic cup formation and invagination occurs, with the neuroretina progressively acquiring a horseshoe shape reminiscent of the inner wall of the optic cup, surrounded by the RPE [143]. Optic cups have been shown to recapitulate the process of topological organization of the retina, displaying all major retinal cell types and photosensitivity [143].

Since the advent of 3D optic cups from mouse embryonic stem cells, pioneered over a decade ago [149], the protocol has been adapted to produce human retinal organoids, for the investigation of cell non-autonomous processes and multi-factorial traits.

Human organoids are multi-layered, light-responsive iPSCs-derived structures with a phase-bright, stratified neuroepithelium that originates from an embryoid body and optic cup-like structures. Following an initial stage of the phase-bright retinal neuroepithelium, pre-organoids develop, on a second stage, into opaque structures, followed by a third stage with brush-like protrusions corresponding to photoreceptors outer segments.

The phenotypic analysis of organoids has proved that the temporal patterning of the retina can be recapitulated in vitro, supporting the notion that TFs-mediated, reciprocal inhibition drives the dichotomic cell fate specification of neuroepithelial progenitors towards the neuroretina as opposed to the RPE, through feedback regulatory loops [150]. *Mitf*-mutant organoids exhibit delayed proliferation during the early stages of development and downregulate the proliferation marker Ki67, but the overall growth is unaffected in the long term except for the fact the RPE develops abnormally and the neuro-retinal marker VSX2 is upregulated [151,152]. Conversely, *Vsx2*-mutant organoids exhibit reduced proliferation at early stages and reduced expression of the marker Ki67, are intrinsically biased towards an RPE cell fate, and upregulate *Mitf*.

Overexpression of *Fgf9* partially rescues the mutant phenotype, and the expression of *Vsx2*, although proliferation remains delayed [144,148].

It has been suggested that *Mitf* expression may regulate the early proliferation of the neuroepithelial progenitors and that accumulation of β-catenin in the dorsal optic vesicle may specify the RPE cell fate through the canonical Wnt/β-catenin pathway, while subsequent repression of *Mitf* by VSX2 would be essential to induce the neuroretina and the optic cup development [148].

Single-cell RNA-seq from the intact retina has identified transcriptional signatures for several non-neuronal cells such as Müller glia, astrocytes, RPE, choroidal melanocytes, microglia, monocytes, natural killer cells, T and B cells, mast cells, pericytes, fibroblasts, and vascular endothelial cells. In developed organoids, most retinal neuronal cell types and some non-neuronal cell types are present, including rods, S cones, and L/M cones, one type of horizontal cells, all ten types of bipolar cells, and 14 of 17 types of amacrine cells typically found in the adult [72]. However, the only non-neuronal cell types that can be detected are Müller cells, astrocytes, and RPE cells. Conversely, immune, vascular-associated, and choroidal cell types are not observed in developed organoids, including pericytes, endothelial cells, melanocytes, fibroblasts, monocytes, T cells, monocytes, and microglia [72]. Overall, retinal organoids have been shown to recapitulate lamination, synaptic function, staging, cell-type diversity, and specificity of gene expression seen in the intact human retina, by single-cell RNA-seq and ATAC-seq comparative profiling, providing a potential model system to investigate retinogenesis and retinal degenerative diseases [69,72].

### 3.4. Lhx2 and Its Neurogenic Potential

Additional evidence indicates that early embryonic ablation of *Lhx2* results in proliferative defects of glial precursors [132] while early post-natal ablation (*Pdgfrα-Cre; Lhx2^fl/fl^*), as well as overexpression by electroporation (*pCAG-Cre; Lhx2^fl/fl^*), result in loss of glial markers and dysmorphic apical structures [153]. Later in development, *Lhx2* loss of function results in forms of reactive gliosis in absence of stress (*RaxCreERT2; Lhx2^fl/fl^*), (*GlastCreERT2; Lhx2^fl/fl^*) and increased susceptibility to light damage by reduced expression of neuroprotective factors [153].

While temporally controlled conditional knockouts indicate that LHX2 induces and stabilizes the retinal glial fate during the first post-natal week of development, by sustaining the expression of the HES5-mediated Notch signalling effectors [132], its function does not seem to be confined to the differentiation and homeostasis of glial precursors: *Lhx2* early embryonic ablation by tamoxifen at embryonic day e10.5, analyzed at e12.5, results in the depletion of multipotent retinal progenitor cells and overproduction of retinal ganglion cells, at the expense of horizontal cells and photoreceptors (*Hes1-^CreERT2^; Lhx2^fl/fl^*). Although, if the ablation of *Lhx2* is postponed to e15.5 and analyzed at postnatal day P0, when the peak of production for retinal ganglion cells has passed, then rods are overproduced [131]. This indicates that LHX2 may contribute to the maintenance of embryonic progenitors in a proliferative and multipotent state, by restricting their exit from the cell cycle and allowing for the generation of later cell types within critical windows [131].

Overexpression of the intracellular domain of Notch signalling, N1^ICD^ by electroporation in the post-natal retina promotes retinal progenitor cells maintenance evaluated by radial morphology and inhibits the expression of gliogenic markers in the wild-type retina (*pCAG-Cre; pCAGGS-N1^ICD^*). When overexpression of N1^ICD^ is coupled with conditional ablation of Lhx2 (*pCAG-Cre; pCAGGS-N1^ICD^; Lhx2^fl/fl^*), Müller glia markers are induced, instead [133]. Furthermore, individual overexpression of the LHX2 transcriptional targets and interactors SoxB1 and SoxC family members *Sox2, Sox8, Sox9* [154,155] inhibits amacrine cells production, although whenever overexpression of *Sox* family members is coupled with *Lhx2* ablation (*Cre-Sox2/8/9: Lhx2^fl/fl^*), amacrine cells production is induced, instead, depleting the retinal progenitor cells [133].

Finally, overexpression of *Lhx2* with its transcriptional co-activator *Ldb1* triggers cell cycle arrest, overproduction of wide-field amacrine cells, and inhibits both Notch signalling and retinal gliogenesis. LHX2 drives expression of several neurogenic bHLHs, among which, *Ascl1* and *Neurog2* [75,156]. In the wild-type retina, RNF12, a negative regulator of LDB1, is necessary for the initiation of gliogenesis, during which the formation of the LHX2/LDB1 complex is reduced [156].

This cumulative evidence indicates that LHX2 regulates the genesis, terminal differentiation, and homeostasis of Müller glia throughout retinal development, although its function is not confined to retinal gliogenesis. Retinal cell fate determination correlates with the time and context of *Lhx2* inactivation and dependence on Notch signalling and LHX2 may control the cell fate determination by deflecting and/or overriding default developmental pathways.

### 3.5. Lhx2 as a Transcriptional Determinant of Cell Fate Identity

Repression release of retinal cell type-specific genes occurs indeed in retinal progenitor cells upon *Lhx2* loss of function (*Chx10-Cre-eGFP; Lhx2^fl/fl^*) revealing underlying, default developmental pathways.

Early embryonic ablation of *Lhx2* in early, flow-sorted retinal progenitor cells induced repression release for genes associated with amacrine cells, retinal ganglion cells, horizontal and bipolar cells, whereas post-natal ablation of *Lhx2* (*pCAG-Cre-GFP; Lhx2^fl/fl^*) induces over-expression of genes associated to rods, cones, and late retinal progenitor cells.

Novel PWMs, found enriched in the LHX2 ChIP-seq datasets at early and late retinal progenitor cell stages, were aggregated by average linkage by increasingly stringent similarity where the minimum correlation threshold for motifs partitioning was set to be 0.6. All major clusters and related roots were then re-assigned to age-matched retinal TFs showing expression by RNA-seq. Multiple motifs instances were found within a broad range of similarity to the known LHX2 consensus and exhibited differential occupancy, possibly underlying differences in affinity and/or combinatorial interaction with other TFs [75]. While LHX2 motifs instances identified by ChIP-seq are homogeneously distributed across retinal categories, high similarity motif instances are slightly prevalent in promoters of retinal ganglion cells specific genes at embryonic stages. Instead, motifs that diverge from consensus are prevalently located in promoters of genes that are enriched in Müller glia and cones. At post-natal stages, high similarity LHX2 motifs instances are slightly prevalent in promoters of rods-associated genes whereas low similarity motifs are slightly prevalent in promoters of retinal ganglion cells and early retinal progenitor cells-associated genes. Low similarity instances identified by ChIP-seq seem underrepresented across all retinal categories at post-natal stages compared to embryonic stages, suggesting a broader diversification of LHX2 binding sites repertoires occurring at early stages. The empirical distribution of LHX2 motifs diverging from consensus across retinal promoters seems to reflect transcriptional dependence of these genes on *Lhx2* expression, with retinal ganglion cells and bipolar cells prevalently de-repressed at embryonic stages and rods derepressed at post-natal stages, following *Lhx2* loss of function.

### 3.6. Chromatin Regulators in Retinal Development

Multiple TFs involved in retinal development have been characterized. Some affect the di- and tri-methylation of histone H3K4, which results in gene activation and is primarily mediated by the family of histone methyltransferases MLL1. Conditional ablation of *Mll1* results in disruption of progenitor cells proliferation, cell-type composition, and neuron–glia balance [157].

Other relevant TFs compose the histone methyltransferase polycomb repressive complex 2 (PRC2) which is known to regulate the maintenance and differentiation of retinal progenitor cells [158,159] by mediating the deposition of the histone repressive mark H3K27me3. Methylation occurs via recruitment of the H3K27 trimethylase EZH2 [117,160], whereas H3K27me3 demethylation is mediated by JMJD3 [161]. H3K27me3 is progressively removed as retinal development proceeds and is paralleled by enhancers’regulation on cell type-specific genes [83].

In the frog retina, expression of the PRC2 subunits is driven by Wnt/β-catenin signalling via the receptor Frizzled 5 and is independent of SOX2. Functional inactivation of the PRC2 core subunits *Ezh2* affects retinal proliferation in frog and mouse, promoting Müller glia cells formation at the expense of neuronal cell types [158,159]. Despite the enrichment of *Ezh2* in retinal ganglion cells, *Atoh7*-driven deletion of *Ezh2* (*Math5-Cre*) does not seem to elicit any defect in retinal ganglion cells development, survival, or homeostasis [162].

Nonetheless, conditional ablation of *Ezh2* (*Pax6-αCre, Six3-Cre*) depletes postnatal retinal progenitors, disrupts retinal lamination, enhances differentiation of photoreceptors and Müller glia, and induces glial reactivity [117]. Embryonic deletion of *Ezh2* in retinal progenitor cells results in photoreceptor degeneration, by de-repression of the transcriptional targets *Six1* and *Eya2* [163]. Furthermore, the TF CASZ1 cooperatively interacts with members of the PRC complex to repress *Lamin A* which is necessary to preserve an inverted nuclear structure in nocturnal rods. Consistently, CASZ1 expands and centralizes the heterochromatin in fibroblasts [164], suggesting that the PRC complex and its cognate factors may have a role in the rods’ genome organization. Finally, inactivation of the PRC2 subunit *Eed* leads to post-natal depletion of multipotent progenitors, a reduced production of Müller glia, bipolar, and rod photoreceptor cells, and overproduction of amacrine cells.

Notably, the former gliogenic phenotype seen with the inactivation of *Ezh2* phenocopies *Lhx2* postnatal ablation, coupled with overexpression of the intracellular *Notch* domain [133]. Glial reactivity is also observed upon *Lhx2* ablation in differentiated Müller glia [153]. The latter phenotype with overproduction of amacrine cells upon *Ead* inactivation, instead, phenocopies *Lhx2* loss of function coupled with overexpression of *SoxB1-SoxC* family members [133].

Besides the PRC2 complex, alteration of the SWI/SNF chromatin remodelling complex is also known to affect chromatin organization on a compartment level [165,166]. The SWI/SNF chromatin remodelling ATPase BRM promotes Notch inhibition, cell cycle exit, and BRN3B-mediated retinal ganglion cell production [167].

The SWI/SNF complex also affects retinal progenitors’ proliferation and photoreceptors’ differentiation through the core component BRG1, which regulates enhancers involved in cell-lineage specification [168,169]. Stage-specific and lineage-specific enhancer elements associate with non-coding RNAs [170], and some have been individually validated for the transcriptional regulation of the TF *Vsx2*, *Prdm1* [171], and *Otx2* [172], whose expression can be driven by ASCL1 or, alternatively, by NEUROG2 [173].

As the advent of CRISPR-based genome editing has enabled ex vivo perturbation of regulatory elements and functional validation of enhancers, more and more *cis*- and *trans*-regulatory elements are now being surveyed and assessed.

### 3.7. Leveraging Next-Generation Sequencing towards a Deeper Understanding of Gene Regulatory Networks and Hierarchies

Pioneer factors are known to target partial DNA motifs on nucleosomes to initiate reprogramming [174,175,176].

Some of these factors were identified by process of elimination, as necessary and sufficient to mediate somatic cells reprogramming to embryonic stem cells-like pluripotency, when ectopically induced [177,178].

Starting with a minimum of two developmental time points, a progressive time-course acquisition of chromatin accessibility can be monitored by ATAC-seq around the motifs center, for any given TF, and binding sites can be validated by ChIP-seq.

**Figure 1:**
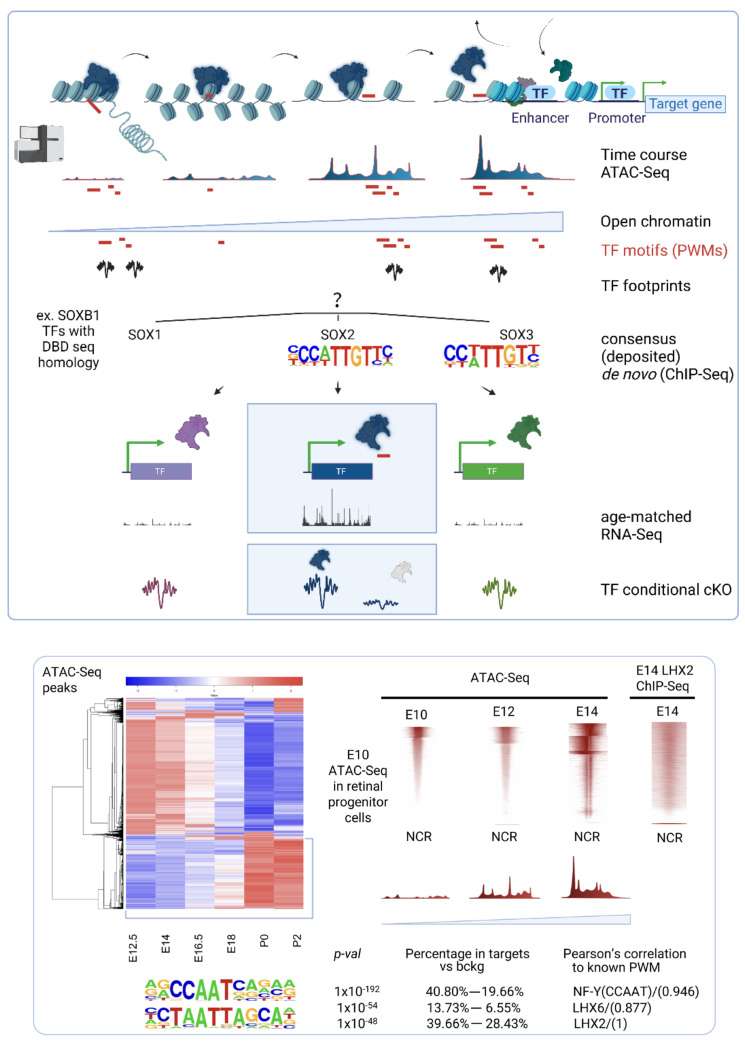
Pipeline to identify pioneer factors. Principles of integration of ATAC-seq with ChIP-seq for any given candidate pioneer factor and age-matched RNA-seq. (Upper panel) Progressive chromatin opening can be monitored in a time-course analysis of open chromatin states by ATAC-seq, integrated with known, deposited motifs and with de novo TFs binding signatures from ChIP-seq (TFs PWMs). Binding sites for TFs that display sequence homology of DNA binding domains (DBD) can be unequivocally assigned to a given TF based on age-matched expression levels by RNA-seq and footprinting analysis comparing control and knock out conditions (cKO). (Lower panel) Supervised hierarchical clustering of open chromatin states identifies clusters of coordinately accessible open chromatin. LHX2 ChIP-seq binding sites are compiled across genome-wide heatmaps of k-means clustered, nucleosome centered (NCR), developmentally regulated, open chromatin regions undergoing progressive opening (blue to red gradient). Representative motif logos and related enrichment are displayed. Besides having a positive pioneer index (PIQ), LHX2 displays signs of competition for nucleosome occupancy. LHX2 may exert a pioneer function at earlier embryonic stages of retinal development (E10, E12) when the induction of open chromatin states anticipates stage-specific targets selection identified by ChIP-seq at E14. Adapted from [75,185] (CC BY 4.0). Credit: Figure 1 was created with BioRender.com.

TFs can be ranked by their likelihood of chromatin opening, scored by protein interaction quantitation (PIQs) [179]. Signs of competition for nucleosome occupancy, or displacement, also emerge from the positive overlay between ATAC-seq nucleosome-centered regions and ChIP-seq for the TF of interest, where ChIP-seq binding sites can be compiled across genome-wide heatmaps of k-means clustered open chromatin regions (Figure 1) (p. 12).

Footprinting enables to detect active TF recruitment across open chromatin regions, where bindings sites can be cross-referenced against deposited, known PWMs, or de novo, empirically detected oligonucleotides by ChIP-seq [180,181,182]. Given the multitude of causative loci identified in complex diseases and their variable penetrance, the identification of TF binding sites can eventually lead to post hoc prioritization of genomic variants in non-coding regulatory regions from genome-wide association studies [183,184], to devise customized, genome editing-based therapeutics for visual restoration.

Comparison of ChIP-seq binding sites with footprints lost upon conditional knock-out for the TF of interest allows unequivocal assignment of binding sites, which is useful in case of consensus similarity across TF family members, due to sequence homology for DNA binding domains (Figure 1) (p. 12).

Furthermore, footprinting analysis of predicted pioneer factors can reflect their developmentally regulated reliance on the TF of interest, by comparing control with TF knock-out conditions [75].

It was noticed that 40% of LHX2 ChIP-seq targets at embryonic stages and 8% of post-natal targets lose accessibility upon *Lhx2* ablation. Furthermore, LHX2 affected chromatin accessibility genome-wide, regardless of its binding patterns, resulting in 48% loss at embryonic stages and 22% at post-natal ones.

**Figure 2:**
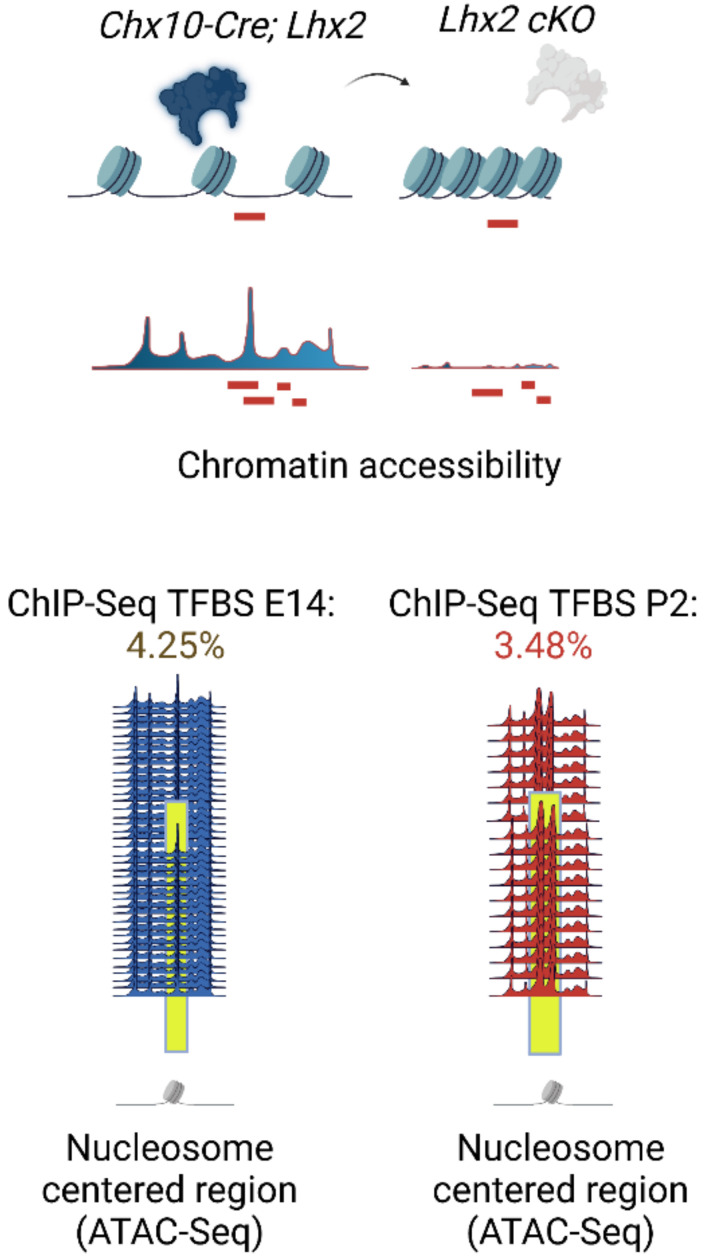
LHX2 competes with nucleosome occupancy by mediating partial opening of chromatin regions. Schematic representing loss of chromatin accessibility following *Lhx2* loss of function (cKO). ChIP-seq binding sites were compiled across genome-wide heatmaps of k-means clustered, nucleosome-centered, open chromatin regions at embryonic day 14 (E14) and post-natal day 2 (P2). Percentage of physical juxtaposition of LHX2 binding sites with nucleosome-centered regions is reported. Global loss of chromatin accessibility exceeds LHX2 binding targets (TFBS). Adapted from [75] (CC BY 4.0). Credit: Figure 2 was created with BioRender.com.

**Figure 3:**
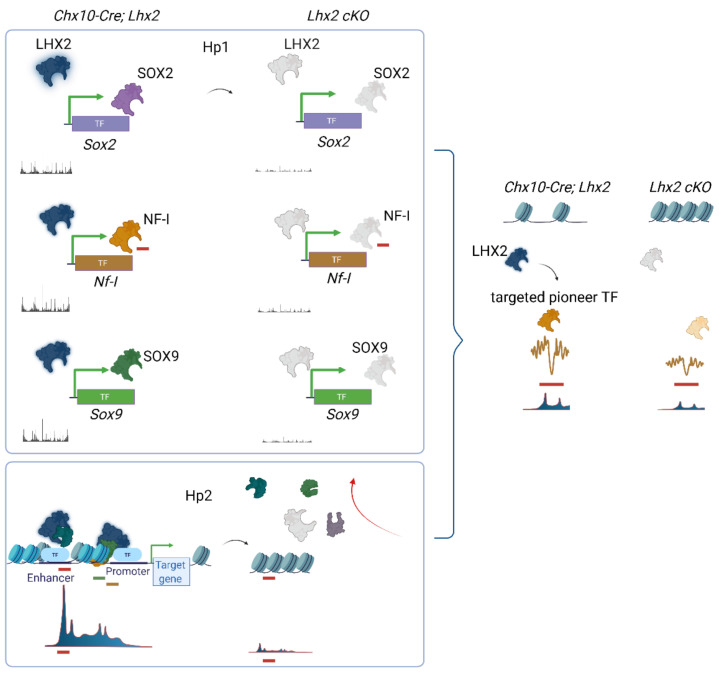
Working hypothesis: secondary regulation of pioneer factors by LHX2 affects genome-wide chromatin accessibility. Global loss of chromatin accessibility following *Lhx2* loss of function exceeds LHX2 binding capacity and can be reconciled with the following hypothesis: secondary, transcriptional, and epistatic regulation by LHX2 (Hp1) or steric regulation of putative pioneer factors by LHX2 (Hp2) contributes to the widespread decrease in chromatin accessibility upon Lhx2 loss of function. Adapted from [75] (CC BY 4.0). Credit: Figure 3 was created with BioRender.com.

**Figure 4:**
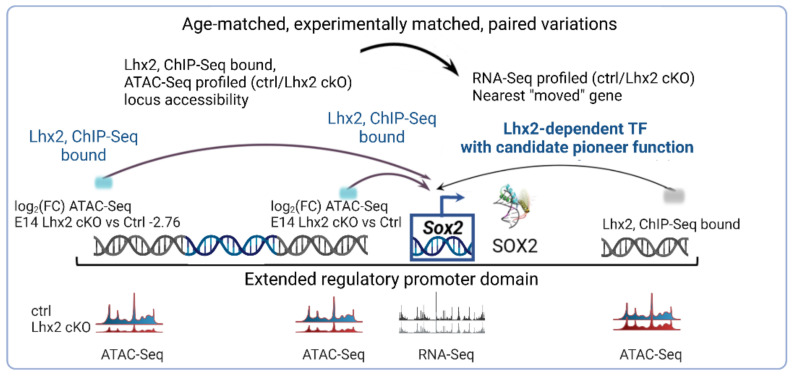
Transcriptional dependence tested (simplified schematic). TFs with high pioneer potential are among LHX2 genetic targets, based on age-matched RNA-seq results from conditional *Lhx2* knock outs (cKO). LHX2 coordinates paired variations in TFs gene expression (RNA-seq) and in chromatin accessibility (fold change [FC] by ATAC-seq) at bound regulatory loci (ChIP-seq) within extended regulatory promoter domains, resulting in epistatic and transcriptional de-regulation of genetic targets including other TFs with pioneer potential. The nearest promoters, LHX2 ChIP-seq peaks were assigned to, were defined by extending TSS regions 5000 bp upstream, 1000 bp downstream for up to 1,000,000 bp max extension. Enhancers were defined by H3K27ac coverage. Adapted from [75] (CC BY 4.0).

Because physical juxtaposition of LHX2 binding sites with nucleosome-centered regions (4% at embryonic stages and 3% at post-natal stages) (Figure 2) cannot explain the global loss in chromatin accessibility following Lhx2 loss of function, it was hypothesized that secondary regulation of LHX2-dependent TFs with high pioneer potential may occur and amplify the phenomenon. Hence, the widespread decrease in chromatin accessibility would result from transcriptional de-regulation of pioneer factors by LHX2 and/or from the loss of steric interaction of such factors with LHX2 [75] (Figure 3 and Figure 4) (p. 14).

**Figure 5:**
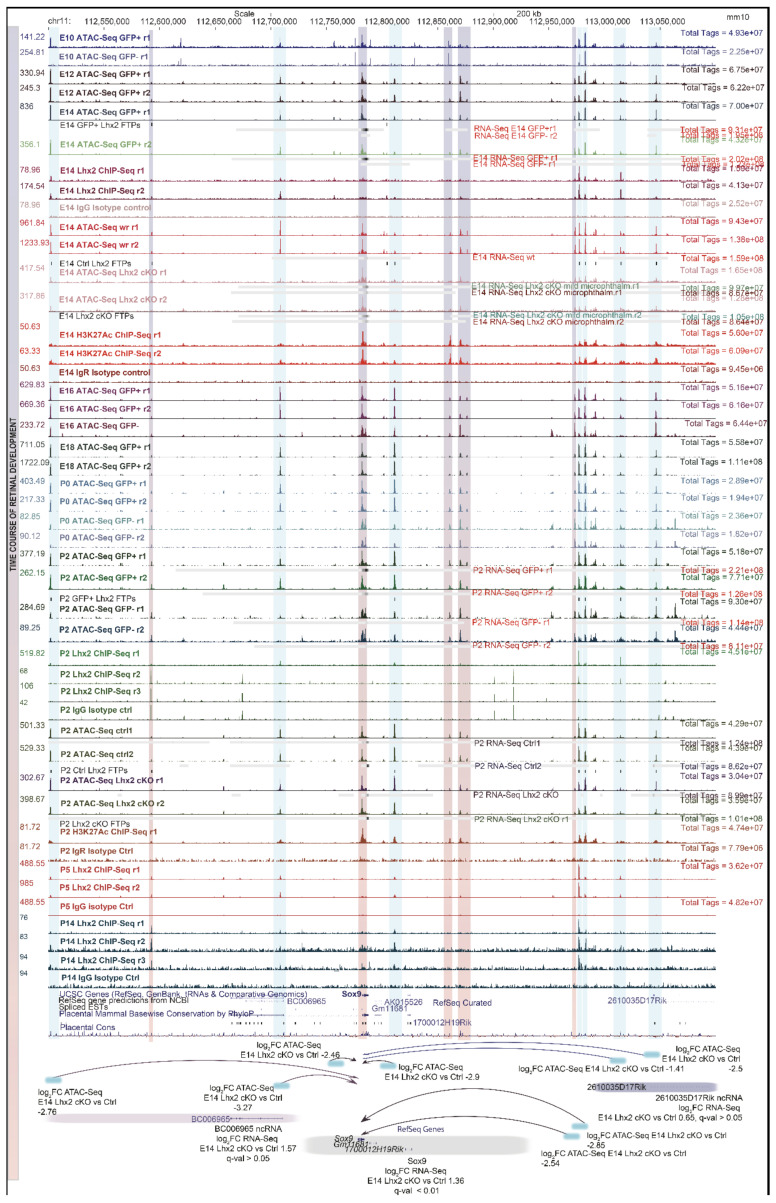
Inferential module of transcriptional regulation for candidate pioneer factor *Sox9*. UCSC custom tracks were configured onto the mm10 mouse genome. A probabilistic, regulatory module of LHX2-coordinated variations in chromatin accessibility can be inferred for any given genetic target from a time-course analysis of differentially accessible open chromatin regions. ChIP-seq for any TF of interest can be integrated with enhancer elements decorated by H3K27ac, with RNA-seq, ATAC-seq and footprinting analysis (FTPs) in control and TF knock out conditions (cKO). As for Figure 4, ChIP-seq-bound targets that overlay with coordinately accessible chromatin loci (altered accessibility upon age-matched TF loss of function) are probabilistically assigned to the nearest gene displaying age-matched, statistically significant variations in gene expression upon TF loss of function. Probabilistic assignment of regulatory loci is allowed to any nearest gene located within extended curated regulatory domains that are defined by extending TSS regions 5000 bp upstream, 1000 bp downstream for up to 1,000,000 bp maximum. This pipeline is virtually applicable to identify genetic regulatory networks and transcriptional hierarchies for any TF of interest in any developmental context. Adapted from [75,185] (CC BY 4.0). SOX9 is among LHX2 genetic and steric targets with predicted pioneer potential. For explicative purposes, blue and red vertical shadows are drawn across regional reads pileups that are developmentally regulated over time. Time-course analysis encompasses ATAC-seq and age-matched RNA-seq at embryonic day E10, E12, E14, E16, E18, P0, and P2 for flow-sorted retinal progenitor cells and post-mitotic fractions (*Chx10-GFP-Cre*). Lhx2 targets are ascertained by comparing LHX2 ChIP-seq readout with ATAC-seq and RNA-seq from age-matched control and conditional knock-out at E14 and P2. Representative LHX2 peaks at P5 and P14 are also displayed.

Enhancer regulatory elements, decorated by H3K27ac marks, bound by LHX2, and transcriptionally accessible by ATAC-seq were identified for several retinal progenitor cell-associated genes such as *Sox2*, *Sox9*, and *Vsx2* (*Chx10*) (Figure 5) (p. 16) [75], and later replicated [98].

SOX2 plays a role in the embryonic and adult central nervous system, where its depletion impacts both neural progenitor cells maintenance and neurogenesis [186,187,188,189,190]. SOX2 is a dose-dependent regulator of retinal neural progenitor competence [191,192], it preserves retinal progenitor cells post-natal pluripotency [154] and it is involved in amacrine and Müller Glia formation from murine retinal progenitor cells [193]. Postnatally, SOX2 is also essential to preserve Müller glia precursors’ radial morphology and cell cycle quiescence [154]. SOX2 and SOX9 are expressed in retinal progenitor cells postnatally and in Müller glia, accounting for 2% of the retinal population [194].

The HMG-box TF SOX9 is essential for the differentiation and survival of postnatal Müller glial cells [195], it regulates genes involved in the visual cycle and the timing of RPE maturation [196,197], it is involved in the proliferation and differentiation in corneal epithelial stem cells [198], it mediates neurogenic-to-gliogenic fate transitions in the cerebellum [199] and astrogliogenesis along the dorsal–ventral axis of the spinal cord [200].

Here, gene regulatory networks are inferred by pairing ATAC-seq peaks of variable accessibility to all nearest genes displaying age-matched statistically significant expression variations, or variations in experimentally matched knock-out conditions (Figure 5) (pp. 17, 18) [75].

Subsequent work from the same group [124] has heavily relied on this first inferential model [75], when constructing gene regulatory networks. The original work does not set a stringent limit to pair peak-to-transcription start sites (TSS), since it enables probabilistic assignment of a given peak up to 5000 bp upstream the TSS, 1000 bp downstream for up to 1,000,000 bp maximum extension. Moreover, it incorporates H3K27ac marks since enhancers can span across megabases of distance from the TSS. A lack of integration with enhancer marks and the overall stringency in peak-to-TSS pairing adopted in the subsequent work, that is upstream 100 kb or downstream 100 kb of the intergenic peaks [124], may lead to proximity-related biases and false-negative assignments. In the subsequent work, prediction of cell type-specific TFs binding in *cis*-regulatory elements [124] also relies on the pairing of TF binding sites based on footprinting scores with corresponding stage-matched TF expression, as proposed in the original work [75].

Last, but not least, what is here referred to as “feedback TF pairs” [124] arises from the fundamental observation described in the original work [75] that TFs can exhibit mutually epistatic and/or steric dependencies and that integration of ChIP-seq, with ATAC-seq and RNA-seq in conditional knock out conditions for any given TF, can address that.

Such regulatory dependencies can be inferred by a) assigning ATAC-seq peaks exhibiting a given TF binding signature (PWM, ChIP-seq binding site, ATAC-seq footprinting) and variable accessibility, to all nearest genes displaying RNA-seq expression variations in experimentally matched knock out conditions for a given TF b) pairing all co-occurring TF binding signatures (PWM, ChIP-seq binding site, ATAC-seq footprints) with footprinting variations in experimentally matched knock out conditions for a given TF.

Simply put, in a positive feedback scenario, for any overexpressed TF1 that positively regulates a given TF2, there would be co-occurrent accessibility of TF1 binding sites, induced gene expression for TF2, and positive footprinting metrics for both TF1 and TF2. When TF1 is downregulated, accessibility of TF1 binding sites would be reduced, expression for TF2 would be downregulated and footprinting signatures for both TF1 and TF2 would be reduced or lost.

It is, therefore, not clear how this work [124] advances the state of the art on gene regulatory networks besides stratifying cellular heterogeneity, and how exactly the authors intend to leverage the new single-cell inventories of *cis*- and *trans*-acting factors to guide cell-based therapies in retinal degeneration.

### 3.8. Footprinting Analysis and Competition for Nucleosome Occupancy by Predicted Pioneer Factors Identify Steric Dependencies and Transcriptional Hierarchies in Cell Fate Specification

**Figure 6:**
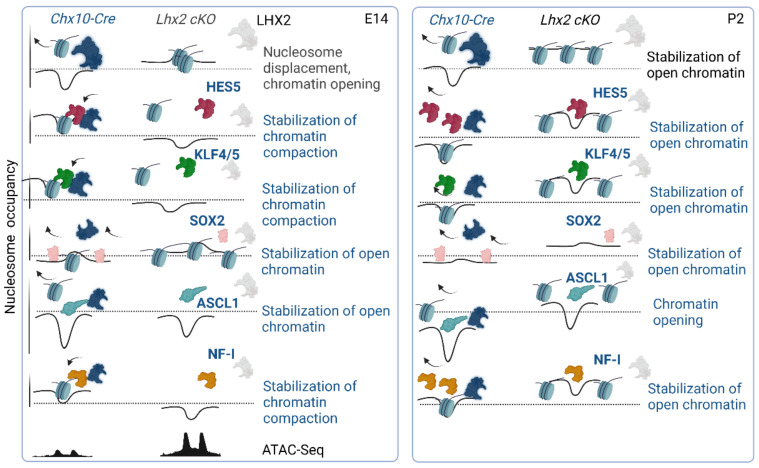
Footprinting analysis and competition for nucleosome occupancy by predicted pioneer factors reflect their developmentally regulated dependence on LHX2. Comparative analysis of ATAC-seq footprinting and nucleosome occupancy in controls and *Lhx2* knock-out conditions (cKO) (*Chx10-GFP-Cre; Lhx2^lox/lox^*) identifies TFs whose genome-wide binding and competition for nucleosome occupancy is sterically affected by LHX2. Steric dependence on LHX2 is first assessed by comparing TFs footprinting metrics in control and *Lhx2* knock-out conditions (absolute counts and reads distribution over 200 bp from motif centers). TFs whose binding associates to progressive, developmentally regulated chromatin opening (positive pioneer PIQ index), hence candidate pioneer factors, are then screened for signs of nucleosome displacement. A schematic is finally derived from genome-wide histograms of ATAC-seq reads, compiled across TF motif centers for candidate pioneer factors. Nucleosomes distribution, or occupancy, is an inferred, specular representation of ATAC-seq reads across motif centers. The lower the distribution of ATAC-seq reads around motif centers is, the higher chromatin compaction is expected to be, since ATAC-seq only profiles, in principle, open chromatin regions. Nucleosome dips of 150 bp circa arise from the depletion of the ATAC-seq signal around motif center because nucleosomes bound chromatin cannot be transposed, resulting in a “train rack” heatmap effect, with visible flanking regions and a central dip in reads coverage, as displayed in Figure 1, bottom panel. A TF with no pioneer potential would result in a relatively homogenous reads distribution around motif centers and a locally limited effect on chromatin accessibility upon loss of function. Conversely, loss of function or destabilization of candidate pioneer factors would result mostly in an altered nucleosome dip (i.e., ASCL1) and higher nucleosome occupancy, hence chromatin compaction, as in [100]. Competition for nucleosome occupancy by candidate pioneer factors is affected by *Lhx2* expression and/or interaction with LHX2 and it is developmentally regulated. Adapted from [75] (CC BY 4.0).

Considering that binding sites for TFs with known and putative pioneer potential are among LHX2 ChIP-seq motifs co-occurrences, steric dependence on LHX2 was tested by footprinting analysis and competition for nucleosome occupancy. Analysis following Lhx2 loss of function has shown that LHX2 may stabilize top candidate pioneer factors prior to and during recruitment at chromatin loci across murine retinal development (Figure 6) (page 19).

Among the computed pioneer factors in retinal progenitor cells, the pro-neural transcription ASCL1 was identified and predicted to mediate chromatin opening by direct competition with nucleosome binding [75].

ASCL1 is a neurogenic bHLH TF expressed in a subpopulation of retinal progenitor cells [87,201], it is known to increase in the fish retina upon injury [202] and its overexpression mediates neuronal differentiation of retinal progenitor cells in dissociated cultured of mouse Müller Glia [194]. While ASCL1-mediated regenerative response seems to be limited to younger mice [194], the addition of an HDAC inhibitor restores the regenerative potential in adult mice in vivo following NMDA-mediated damage [203].

Ultimately, the model postulated here based on a totally agnostic screening reconciles with these findings [203], indicating that LHX2, and negative regulators of the HDAC complex, may stabilize ASCL1 and enable chromatin opening.

Among all candidate regulators of chromatin accessibility in the retina, SOX2 is predicted to stabilize chromatin opening and ablation of Lhx2 results in destabilization of SOX2 and an increase in nucleosome occupancy at early embryonic and post-natal stages [75]. Later work has shown that downregulation of *Sox2* in rod photoreceptors is accompanied by deposition of the repressive histone mark H3K27me3 on specific loci [98], consistent with the above findings [75]. Furthermore, KLF4 is predicted to stabilize chromatin compaction early in development while mediating chromatin opening at post-natal stages [75]. KLF4, similar to SOX2, is a pluripotency factor, as it was shown to mediate somatic reprogramming into induced pluripotent stem cells [177,204,205,206]. *Klf4* repression release occurs upon downregulation of miRNA let7, which is necessary to mediate a regenerative response in the retina upon injury [194]. *Klf4* is also expressed in Müller glia and is induced in the chick and fish retina upon injury [207,208,209]. *Klf4* is expressed in retinal ganglion cells that are primarily affected in glaucoma and optic neuropathies. *Klf4* expression in retinal progenitor cells and post-mitotic precursors does not seem to affect retinal ganglion cells neurogenesis, suggesting that underlying compensatory mechanisms may exist, mediated by functionally redundant Kruppel-like family members. Nonetheless, *Klf4* overexpression does decrease neurite outgrowth, in hippocampal and cortical neurons. Conversely, conditional ablation of *Klf4* (*Chx10-Cre; Klf4^fl/fl^*) results in increased axon growth both in vitro and in vivo, after optic nerve injury, suggesting KLF4 may repress axon regeneration related pathways in retinal ganglion cells upon injury [210,211].

LHX2 binds within modules of coordinately accessible chromatin in retinal progenitor cells targeting promoters and non-coding elements in nucleosome-free regions associated with H3K27ac active enhancers marks. These modules are functionally enriched for genes involved in axonal dystrophy, among other identified functions at post-natal murine stages. LHX2 is known to affect thalamocortical axon guidance by regulating Robo1 and Robo2 receptors [212]. In *Robo1*, *Robo2* mutants, mimicking retinopathy of prematurity, retinal ganglion cell axon guidance is also disrupted, and they display abnormal retinal vascularization, due to the lack of directional information to migrating astrocytes, [213].

Notwithstanding, targeted overexpression of *Klf4* in the retina of neonatal rats is sufficient to reprogram the identity of late retinal progenitor cells towards retinal ganglion cells, by partially reactivating relevant transcriptional networks outside their developmental window [214].

*Klf4*, like *Sox2* and *Sox9*, displays both transcriptional and steric dependence on LHX2 [75]. Additional retinal ganglion cells related determinants, such as *Pou4f2*, *Atoh7*, and *Isl1* [215,216,217,218] are also transcriptionally and/or sterically targeted by LHX2. Retinal ganglion cell-specific genes are released from repression in *Lhx2* conditional knock outs at embryonic stages (*Chx10-Cre; Lhx2^fl/fl^*), when relevant promoters are targeted by LHX2 [75], and early embryonic ablation of *Lhx2* (*Hes1-Cre ERT2; Lhx2^fl/fl^*) in E10.5 mice results in an abnormal production of retinal ganglion cells at the expense of later generated cell types [131].

This cumulative evidence suggests that the interplay between LHX2 and KLF4 may control the onset of retinal ganglion cells by instructing retinal progenitor cells competence within restricted temporal windows. LHX2-KLF4 co-occurring binding patterns may potentially reflect *cis*-and *trans*-regulatory determinants of retinal ganglion cells’ identity and await functional testing.

Some of these findings have been furthered by subsequent studies from other groups, showing that primary enhancers and super-enhancers have a synergistic role in sustaining and grading *Atoh7* expression during critical windows for retinal ganglion cell neurogenesis and they account for species-specific differences in developmental plasticity between mice and humans. Redundant deployment of multiple regulatory elements in mice may provide some buffering capacity, by limiting drastic fluctuations in *Atoh7* expression which would otherwise result in retinal ganglion cell loss, a threshold effect seen both in mice, with the *Atoh7* mutants and in humans, with congenital blindness due to optic nerve aplasia [219]. Similar threshold effects have been observed for *Lhx2* in gliogenesis [75,132,156]. Subsequent work has shown that MEIS1, identified in retinal progenitor cells [75], is predictive of efficient retinogenesis in stem cells models [97] and that MEIS1 and MEIS2 function redundantly to promote the expression of retinal progenitor cell-specific genes, through cooperative interaction with LHX2 and other retinal progenitor cells specific TFs [220].

Among the identified, candidate pioneer factors, this work also shows the first evidence that NF-I may play a role in murine retinal progenitor maintenance and identifies NF-I TFs as candidate regulators of temporal patterning in the developing retina, proposing the mechanism by which they regulate changes in retinal progenitor competence, stabilizing chromatin compaction at embryonic stages while mediating chromatin unfolding at post-natal stages [75].

Thereafter, three derivative studies from the same group have focused on the NF-I regulatory role in retinal progenitor cells, by showing that: (a) NF-I TFs are selectively expressed in late retinal progenitor cells where they control cell-cycle exit, they regulate the generation of late-born retinal cell types, such as bipolar interneurons and Müller glia [127]; (b) disruption of NF-I TFs, which maintain and restore quiescence, induces Müller glia to proliferate and generate neurons in adult mice after injury [221]; and (c) NF-I TFs induce increased accessibility at regulatory sites associated with genes expressed in late-stage retinal progenitor cells and Müller glia at post-natal stages, while NFI loss of function may produce the opposite effect [124]. Despite the fact *Nfib* is expressed by late embryonic stages [127], stage-specific NF-I binding by embryonically staged ChIP-seq is not being assessed. Considering footprinting does not necessarily recall all the ChIP-seq binding sites, as it is led on ATAC-seq-capturing regions of open chromatin, some of the late retinal progenitor cells related NF-I targets are likely to remain undetected, limiting the informativity of the study [124].

NF-I TFs are known to control gliogenesis in the cortex and in the spinal cord [200,222,223,224] and a deeper understanding of the underlying regulatory networks holds promise for possible regenerative therapies.

To summarize, epigenomic profiling of retinal progenitors identifies LHX2 as a central regulator of chromatin accessibility in retinal progenitor cells across several developmental time points and indicates LHX2 controls the timing of retinal neurogenesis by co-opted action of previously uncharacterized, developmentally regulated TFs with high pioneer potential [75].

The knowledge of these mechanisms can be leveraged for the investigation of evolutionarily conserved and divergent mechanisms in humans, paving the way for the restoration of the visual function upon injury.

## 4. Tackling the Epigenetic Contribution to Ocular Diseases for Restoration of the Visual Function: From Corrective Therapies to Autologous Cell Replacement through Comparative Genomics

### 4.1. Target Identification and Repurposing of Epigenetically Relevant Pharmacological Compounds via Drug Delivery Nanosystems

Open chromatin profiling can identify pre-symptomatic configurations of the genome leading to disease progression [100]; variations in chromatin accessibility are predictive of retinal developmental stages [75,119,128,225], as well as disease aggravation [226].

Age-related macular degeneration (AMD) is the leading cause of severe vision loss and blindness affecting the elderly in the developed world and accounts for one-third of all cases of untreatable vision loss. Globally, of the 7.33 billion people alive in 2015, an estimated 36 million were blind, 216 million people had moderate to severe visual impairment, and 188 million had mild visual impairment [227]. AMD accounts for 8.7% of all cases of blindness worldwide [228], has a global prevalence of 170 million individuals and the disease is predicted to affect 288 million individuals by 2040, due to an increased life expectancy.

AMD is a multifactorial, progressive disease that impacts the macular, central region of the retina responsible for visual acuity, affecting the photoreceptors and RPE: it causes destructive and irreversible changes in sharp-sightedness. Asymptomatic in its early stages, some patients experience acute vision loss, blurred vision, scotomas, metamorphopsia, and chronic visual distortion. Different therapeutic approaches are currently available for wet, exudative AMD associated with choroidal neovascularization and for dry, non-exudative AMD, associated with pigmentary abnormalities and deposits (drusen), but no treatment can permanently repair and restore the cellular damage.

The RPE is among the hallmarks of dry AMD accounting for 90% of all cases for which no curative treatment is available prompting the need to develop stem cell therapies to replenish the tissue lost and restore the visual function.

The RPE is involved in light absorption, ions buffering, metabolites recycling, photoreceptors outer segments phagocytosis, secretion, and immune modulation. Inflammatory pathways, activation of the complement system, hypoxia, alterations of the RPE tight junctions involved in the retina–blood barrier, cholesterol metabolism, and dysfunctional rates of photoreceptors outer segments clearance by autophagy or phagocytosis, are all contributing factors to drusen accumulation and to the pathogenesis of AMD [229,230,231,232,233].

The first comparative profiling of macular and peripheral retinal tissue from healthy donors and AMD patients has furthered the understanding of the epigenetic contribution to the progression of age-related macular degeneration, indicating that a widespread decrease in chromatin accessibility occurs during disease progression, and the RPE is affected first [226]. Gene ontologies enriched in differentially accessible chromatin were found devoted to inflammatory pathways, metabolism, and homeostasis. It was later shown, by bisulfite pyrosequencing, that differential DNA methylation occurs in AMD affecting a proto-oncogene involved in TGF beta signalling (*SKI*), and transcription-dependent DNA repair mechanisms (*GTF2H4*) [234]. Following this first evidence by footprinting analysis showing that TFs binding is directly affected in human photoreceptors and RPE during macular degeneration [226], subsequent studies have investigated the impact of genome-wide associated variants and possible single nucleotide polymorphisms on TF mediated regulatory networks and binding sites in the human retina [70,235]. Hence, the candidate *cis*-regulatory sites identified so far may drive the prioritization of non-coding *cis*-regulatory elements in the aetiology of retinal degeneration [184].

Loss of chromatin accessibility correlates with disease severity and exacerbates as disease progresses in an isogenic background [226].

Inflammatory, oxidative stress paradigms in iPSC-derived RPE, as well as overexpression of the histone deacetylase HDAC11 can recapitulate the reduction in chromatin accessibility observed in AMD, consistent with previous observations that: (a) the temporal progression of photoreceptors cell death is accompanied by specific enzymatic activities [236,237]; (b) degenerating photoreceptors trigger an epigenetic program involving de novo DNA methylation and a simultaneously increased HDAC activity, to possibly minimize transcription, protein biosynthesis, hence energy expenditure, in face of a reduced metabolic supply during cell death [238,239,240,241]. Hence, the identification of early events in the pathogenesis of AMD may help devise preventive, therapeutic strategies.

Besides AMD, epigenetic changes have been explored in retinitis pigmentosa, glaucoma, and diabetic retinopathy [242,243,244,245,246]. DNA hypermethylation of genes involved in cell death and survival, cell morphology, and nervous system development has been described in mouse models of retinitis pigmentosa and it is thought to be primarily mediated by the DNA methyltransferase DNMT3a, affecting the expression levels of the genes encoding the TFs *Yy1*, *E2f3*, and *Nrl* [241]. DNA hypermethylation can be paralleled by histone hypoacetylation, hypermethylation and poly-ADP-ribosylation [241,247,248]. Histone-deacetylases inhibitors can mediate neuroprotection by reducing photoreceptors cell death in some models of retinitis pigmentosa, although functional discrepancies have been reported [249,250,251] and pharmacological inhibition of DNA methylation can rescue photoreceptors cell death in organotypic retinal explants. This evidence suggests that inhibition of DNA methylation and, more broadly, manipulation of epigenetic modifiers by the repurposing of currently available pharmacological compounds, could be leveraged for treatment of genetically heterogeneous forms of retinal degeneration.

Nanoscale exosomes can be vehicles for several therapeutic cargos, from small RNAs such as siRNAs and miRNAs, to proteins and neurotrophic molecules, decoy receptors and pharmacological agonists [252].

Lipidic nanocarriers and viral-mediated delivery strategies have also been implemented over time to improve the overall bioavailability, stability, and half-life of the pharmacological compounds in the posterior segment. This has allowed to circumvent the necessity for frequent, topical treatment or intravitreal injections, due to a precocious degradation and clearance of therapeutic molecules [253,254].

Among the viable drug delivery routes utilized to suppress VEGF-mediated angiogenesis, lipidic nanocarriers have been engineered to silence the expression of the human antigen R (*HuR/Elavl1*), encoding an RNA-binding protein known to be involved in diabetic retinopathy, suggesting a similar approach could be leveraged for the treatment of wet age-related macular degeneration and diabetic macular oedema [255]. Ocular angiogenesis has also been tackled by mimicking pharmacologic VEGF inhibition through lentiviral and adenoviral delivery of siRNA and miRNA, or by inducing the expression of the anti-angiogenic PEDF [256,257].

Current considerations for viral vector design and eligibility for pre-clinical trials include retinal tropism, choice of serotype, DNA packaging capacity and transduction efficiency in the cell type of interest, persistence of gene expression, risk of host genome integration, reduced immunogenicity, and off-target effects [253,258,259].

More recently, the advent of CRISPR-mediated gene therapy and genome editing has enabled to tentatively expand the portfolio of therapeutic approaches from monogenic to multi-factorial ocular diseases, to address aberrant gene expression and pathogenic, non-coding variants involved in choroidal neovascularization, altered vascular permeability, lipid homeostasis, neuronal apoptosis or detoxification of reactive oxygen species, and to leverage neuroprotective strategies for the restoration of the visual function [260,261].

### 4.2. Expanding the Therapeutic Portfolio from Gene Editing to Genome Editing towards Personalized Medicine

Several gene editing strategies have been proposed to restore the visual function, depending on the clinical presentation. While gene therapy by subretinal injection is suitable to restore deficient or haploinsufficient copies of a gene and is the elective treatment for monogenic, genetically inherited, recessive forms of blindness, such as Leber congenital amaurosis [262], alternative, corrective strategies are necessary to treat dominant forms of blindness, such as inherited forms of macular degeneration, dominant forms of retinitis pigmentosa and glaucoma. In such case, a suppression and replacement therapy or direct gene editing have been attempted by lentiviral or adeno-associated viral delivery (AAV) of CRISPR-Cas9 [263,264,265,266,267], where a customizable Split–Cas9 system may enable reversible genome engineering [268] and cell type-specific delivery with tissue-specific promoters [269].

With regard to non-coding variants, enhancer de-regulation has been reported, for instance, in autosomal recessive congenital retinal nonattachment, where ablation of a non-coding enhancer regulatory region 20 kb upstream the proneural bHLH TF *ATOH7* gene [270] leads to retinal ganglion cells degeneration and optic nerve atrophy [271].

As mentioned before, ablation of the murine ortholog fails to recapitulate the pathogenic phenotype seen in humans, suggesting that lack of functional equivalence between species may be due to the compensatory deployment of redundant regulatory elements in mice [219].

Mutations of non-coding regulatory elements nearby the *PAX6* gene result in haploinsufficiency due to enhancers de-regulation and have been reported in aniridia and congenital cataract [272,273]. Mutations of the enhancer located 150 kb downstream from *PAX6* disrupt the autoregulatory loop controlling *PAX6* expression in the developing ocular structures.

Another instance of enhancer deregulation emerges from point mutations of CRX binding sites that are proximal to the *SAMD7* gene and are necessary to mediate its expression in photoreceptors. Mutations of CRX binding sites result in *SAMD7* transcriptional deregulation and retinitis pigmentosa [274]. Additional non-coding regulatory elements have been linked to retinitis pigmentosa, and putatively assigned to *EYS, LCA5* and *PCDH15, NMAT1, ABCA4*, *OFD1*, *CEP290*, *USH2A*, and *PROM1*, while other mutations have been identified in the seed region of microRNA-204 and within an open chromatin region located upstream of *PRDM13* [274].

Cryptic enhancers and consequent rewiring of enhancer-promoter interactions can emerge, instead, from the deregulation of TADs, due to some structural variations detected in autosomal dominant forms of retinitis pigmentosa. Structural variations were modelled in iPSCs and retinal organoids and assayed by HiC, resulting in ectopic contacts between retinal enhancers and *GDPD1*, a gene involved in lipid metabolism, and loss of regulatory insultation for *YPEL2* [275].

Advancements in genome-editing technologies may enable to survey all candidate regulatory elements that segregate with disease. Functional dissection of risk-associated alleles can be carried out in retinal organoids and human iPSCs paired with isogenic controls, which allows the comparison of chromatin dysregulation in a virtually identical genetic background.

The translational potential of epigenetics ultimately lies within the functional dissection of non-coding regulatory variants of the genome, which can then lead to the prioritization of such loci for cell type-specific gene therapy and for personalized, genome editing-based medicine.

First, classification of developmentally regulated and disease-related regulatory elements can be leveraged for construct design, to ensure that cell-type specificity of transgene expression is attained when combined with viral vectors exhibiting broad tropism, as to minimize off-target effects of gene therapy.

Second, by leveraging all known *cis*- and *trans*-regulatory sites, CRISPR-Cas9 can be repurposed for simultaneous, reversible gene activation or silencing by conjugating the catalytically inactive caspase9 dCas9 to an effector protein [276,277,278]. A short guide RNA (sgRNA) with a protospacer adjacent motif (PAM) complementary to the targeted genomic sites can then direct the dCas9-KRAB-Effector transcriptional suppresser [279] or the dCas9-SAM synergistic activation mediator [280], to specific genetic loci of interest.

Third, for complex, polymorphic traits, the variability in the phenotypic penetrance of non-coding variants can complicate the diagnosis and the aetiological identification of the causative loci and related genes.

Whenever the genetic burden for some single nucleotide polymorphisms (SNPs) and rare variants cannot be clearly assessed, then several epigenomic features could be leveraged. Overlaying epigenomic features with SNPs may help prioritize specific regulatory loci to ultimately devise CRISPR-Cas9 or CRISPR-Cpf1 genome editing-based strategies [281] for restoration of gene expression. Ultimately, the epigenomic readout and genome-editing strategies could be translated into clinical practice, by orienting patient-tailored therapies in precision medicine.

### 4.3. Agnostic Approaches to Restore the Visual Function, from Prosthetics to Autologous Cell Replacement

Among the agnostic approaches that do not require any prior knowledge of the causative gene or regulatory loci instead, the advent of optogenetic strategies has enabled to restore light sensitivity by delivery of genetically encoded opsins, re-sensitization of photoreceptors, and sensitization of bipolar and retinal ganglion cells, although with limited spectral sensitivity, the only caveat being the reliance on functional, residual cells to relay the signal for visual processing. Potential treatment by optogenetics has been successfully attempted for the treatment of retinitis pigmentosa [282].

Likewise, bionic prosthesis relying on residual cells has been used to augment synaptic transmission, where phosphenes enable the perception of directional movements and shapes with limited resolution [283].

Replacement therapies have been leveraged for treatment of dry age-related macular degeneration, relying on stem cells reprogramming ex vivo, and subsequent autograft that is amenable for RPE transplantation [284] but has limited applicability to retinal neurons, due to the lack of proper integration into the retinal visual circuitry [285].

Furthermore, while iPSCs-derived organoids have proved to recapitulate well the lamination, cell-type diversity, light responses, and single-cell transcriptional profiles of the whole human retina [72], they lack functional foveal-to-peripheral regionalization and representation of vascular-associated, choroidal, and immune cell types, potentially limiting oxygenation and impacting oxidative phosphorylation.

This limits the usage of organoids as potential model systems or, at least, it should warrant some caution when modelling regionalized forms of retinal degeneration, such as AMD that affects primarily the macula, or autoimmune-related disorders associated with ocular manifestations, neovascularization, and an altered retinal permeability.

Finally, reprogramming strategies in vivo have allowed to study the molecular mechanisms underpinning retinal regeneration, or lack thereof, in different model organisms, shedding light on evolutionarily conserved and divergent mechanisms in cell responses upon injury.

### 4.4. Chasing the Secret to Youth: Is the Evolutionary Conservation of the Regulatory Elements in the Genome Truly Key to Unlock the Regenerative Potential of the Retina?

The regenerative potential of the retina varies considerably across species, developmental time frames, and cell types utilized as elective sources for reprogramming. Neurogenic progenitors can be regenerated upon injury from the RPE, the Müller glia, and the ciliary margin zone in nonmammalian vertebrates.

**Figure 7:**
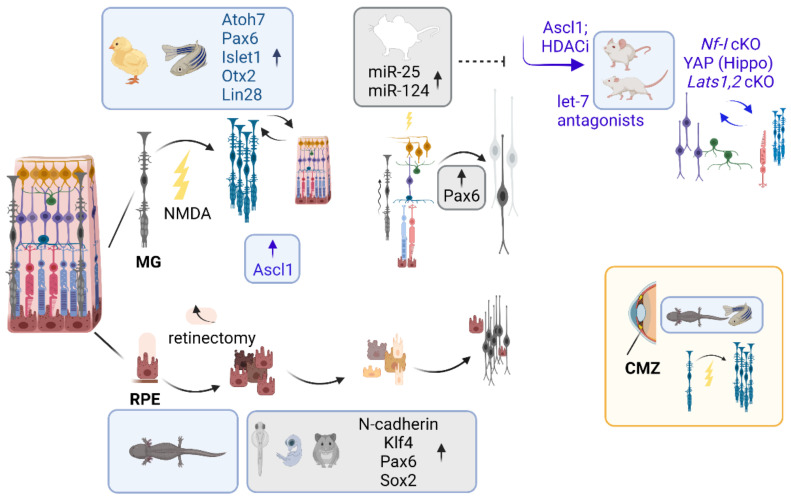
The regenerative potential of the retina varies across species. Neurogenic progenitors can be regenerated upon injury from the retinal pigmented epithelium (RPE), the Müller glia (MG), and the ciliary marginal zone (CMZ) in nonmammalian vertebrates, such as amphibians and fish. In the amphibians, the RPE undergoes dedifferentiation upon retinectomy, depigmentation, and detachment from the basement membrane. The deriving progenitors proliferate and regenerate the neural retina, yet RPE markers are partially retained. In the teleost fish and chick retina Müller glia are the major source of reprogramming instead, where they can dedifferentiate, re-enter into the cell cycle, and acquire neural progenitor cell-like fate upon NMDA excitotoxic injury. Müller glia in mice can partially respond to injury by migrating to the lesion site and upregulating *Pax6*, although they do not fully re-enter the cell cycle, unless specifically manipulated to do so, especially in adult mice. Induction can be driven by *Lin28*/*Ascl1* overexpression combined with HDAC inhibitors, NF-I ablation, or Hippo pathway inactivation. Even then, reprogrammed Müller glia are intrinsically biased towards bipolar, amacrine cells, and a minor proportion of cones (Adapted from [89]).

While the ciliary marginal zone has regenerative potential in amphibians and fish [286] and, to a minor extent, in mice [287], the RPE is the primary source for regeneration in amphibians, such as salamanders and larval frogs [89]. Upon retinectomy, the RPE undergoes dedifferentiation, depigmentation, and detachment from the basement membrane. The deriving cells proliferate as neurogenic progenitors [288,289,290] to ultimately replace the neural retina, with proper lamination and proper ratio of cell types, and they restore a functional RPE. However, newly generated neurons transiently retain expression for RPE markers [89,291] (Figure 7). Thymidine labeling and transgenic lineage tracing indicate that the RPE re-enters the cell cycle and gives rise to a newly derived retina in amphibians [289,292], although the molecular mechanisms underlying amphibian RPE dedifferentiation have not been elucidated. RPE dedifferentiation is accompanied by loss of mature RPE morphology and up-regulation of progenitor markers such as *N-cadherin*, *Klf4*, *Pax6*, and *Sox2* [89,288,293] (Figure 7) (page 26).

Outside of amphibians, the potential of the RPE to generate retinal neurons is restricted to early embryonic stages in fish [294,295], chicken, and mammals [296,297] and even then, it requires substantial manipulation [89].

Within a limited embryonic window, the RPE and Müller glia can be induced to dedifferentiate, proliferate, and reprogram into retinal progenitor cells by mitotic growth factors (EGF) and fibroblast growth factor (FGF) [298].

Müller glia are the major source of reprogramming in the teleost fish and chick retina, where they can dedifferentiate, re-enter into the cell cycle, and acquire neural progenitor cell-like fate upon excitotoxic injury. Müller glia reprogramming in fish occurs through the induction of retinal progenitor cells related TFs such as *atoh7*, *pax6*, *islet1*, and *otx2* [207,221,299,300,301]. Upregulation of the pluripotency marker *lin28*, which represses the miRNA let7 [202] enables the zebrafish Müller glia to revert to a truly pluripotent state when they can give rise to the full repertoire of retinal cell types.

The chick Müller glia can robustly proliferate after injury, although it predominantly regenerates amacrine cells and fails to induce *OTX2*, a TF essential for the photoreceptor cell fate [208,299].

The mammalian RPE and Müller glia instead display an intrinsic reprogramming ability early in development but, as retinogenesis proceeds, they become reluctant to proliferate after injury and, in the adult, they exhibit limited cellular pliancy, and retain terminally differentiated features [89,297,302,303].

Transgenic lineage tracing indicates murine Müller glia can also partially respond to injury by migrating to the lesion site and upregulating *Pax6*, but do not fully re-enter the cell cycle [89,304,305].

A time-course analysis of miRNA from FACS-purified murine retinal progenitor cells at post-natal day 2 (*Sox2: tdTomato^+^*) and Müller glia at subsequent weeks of postnatal development (*Rlbp1: tdTomato^+^*) has revealed enrichment for miRNA (miR)-25 and miR-124.

The neurogenic function for some of the identified miRNA has already been described in the nervous system [306,307,308,309,310], indicating miRNAs as a potential tool for regenerative therapies. Overexpression of miRNA miR-25 and miR-124 in young Müller glia cultured from reporter mice (*Ascl1-CreER: tdTomato^flSTOP/flSTOP^*) elicits neurogenic conversion through downregulation of let-7 and induction of *Ascl1*. The combination of the let-7 antagonist with miR-25 and miR-124 analogs leads to a further increase in *Ascl1* expression in the Müller glia, although the regenerative response was limited when tested in adult Müller glia in vitro [194], suggesting additional mechanisms may be needed to fully recapitulate the regenerative process.

Several groups have comparatively profiled Müller glia upon injury, in the attempt to identify the key differences and epigenomic determinants of their regenerative response across species. Assuming functional equivalence of orthologs and related regulatory loci, they ultimately attempted to manipulate the evolutionarily conserved elements that are responsible for progenitor quiescence and for limitations to the neurogenic potential [124,128,221,311,312,313,314].

Notwithstanding, the experimental paradigms of retinal acute damage that are currently in use for animal models may not adequately recapitulate the degenerative response in humans.

Recent evidence indicates that high-intensity, acute light damage causes Müller glia reactivity in zebrafish, whereas low, continuous light exposure damages the photoreceptors’ outer segments and evokes microglia recruitment, but it does not cause apoptotic cell death, Müller glia gliosis, or cell cycle re-entry [315].

Furthermore, ablation of the retinoblastoma gene *RB* evokes different proliferative responses in human and murine cone precursors [316]. Human maturing cone precursors are propense to re-enter the cell cycle, proliferate, and give rise to quiescent retinomas or retinoblastoma tumors, following *RB* loss of function. Conversely, immature, and mature cone precursors of the *Rb*-ablated mouse retinae, re-enter the cell cycle but are reluctant to proliferate and give rise to retinoblastoma-like lesions.

Moreover, the ectopic expression of the oncogenes *Mdm2* and *Mycn* is not sufficient to initiate oncogenesis in murine cone precursors, which could be explained with stage-specific and species-specific sensitivities to mitogenic signalling.

In zebrafish, chick, and mice, Müller glia re-enter a reactive state upon injury, although species-specific differences were noticed. While zebrafish Müller glia reverts to a retinal progenitor cell-like state by overexpression of *six2b*, *tgif1*, and *lepb*, to undergo proliferation and neurogenesis, murine Müller glia only transiently express cell cycle regulators and neurogenic factors before re-entering quiescence [221].

Mammalian Müller glia, therefore, cannot spontaneously de-differentiate and trans-differentiate in response to retinal injury, especially in adult mice, unless specifically manipulated to do so, through *Ascl1* overexpression coupled with HDAC inhibitors [119,225,317], *Nf-I* repression [221], *Lin28/Ascl1* overexpression [318], or Hippo pathway inactivation [319].

Upon acute injury, murine Müller glia acquire transcriptional and epigenomic signatures that are reminiscent of those seen in retinal progenitor cells, yet the regenerative potential is primarily limited to proliferative Müller glia and neurogenic Müller glia giving rise to bipolar [319], amacrine-like cells, and a minor proportion of cones [221]. Moreover, coupling EdU positive and negative labeling with lineage tracing has revealed that only part of the newly generated neurons derives from mitotic divisions, while others arise from direct trans-differentiation of Müller glia, suggesting additional mechanisms are needed to fully de-differentiate the adult Müller glia.

It has been proposed that the loss of neurogenic gene expression and motif accessibility during glial maturation may prevent efficient reprogramming, accounting for the bipolar fate restriction. However, by comparing open chromatin landscapes in bipolar cells and rod photoreceptors with those in retinal progenitor as opposed to Müller glia, the degree of similarity in accessible chromatin was not sufficient to explain the bias towards bipolar cells seen in Müller glia. The bias towards bipolar neurons appears to be preferentially driven by ASCL1 recruitment on chromatin accessible regions [119] and the neurogenic potential of Müller glia upon *Ascl1* overexpression seems to be restricted by the STAT3 signalling [225]. Furthermore, diversifying the combination of neurogenic bHLH TFs enhances Müller glia trans-differentiation and neurogenesis in vivo. Müller glia co-expressing the bHLH TFs *Atoh1* and *Ascl1* give rise to bipolar and amacrine cells and immature retinal ganglion cell-like cells in the absence of injury, although cell bodies fail to migrate to the ganglion cell layer and the cells have only small axonal processes [320].

Finally, recent evidence has shown that the ectopic expression of the Yamanaka factors reverts the epigenetic clock by restoring transcriptomic patterns and resetting DNA methylation status, by upregulating the DNA demethylases *Tet1* and *Tet2* in retinal ganglion cells and promoting axon regeneration in vivo, in a mouse model of glaucoma with experimentally induced intraocular pressure. DNA methylation also occurs in senescent mice, targeting signature CpG sites enriched for the binding of polycomb repressive complex 2 (PRC2) and its histone methyltransferase product H3K27me3 [321]. Knock-down of the DNA demethylases *Tet1* and *Tet2* affects the methylation status of the CpG sites in a similar way to senescent mice, impacting the expression of *Stat3* and abrogating the ability of retinal ganglion cells to regenerate upon optic nerve crush injury. Yet, overexpression of the DNA demethylases has no protective or regenerative effect on challenged retinal ganglion cells, suggesting additional mechanisms are needed to circumvent aging and to fully restore pluripotency upon injury [321].

Ultimately, the phylogenetic divergence of *cis*- and *trans*-regulatory elements across genomes and the epigenetic retention of cellular origins may account for species-specific differences and for intrinsic biases towards a given cell fate. Additionally, the lack of functional equivalence across orthologs despite sequence homology, possibly due to TFs rewiring and evolutionary repurposing of the related regulatory sequences, could explain cell fate restrictions, differences, and constraints to the endogenous, regenerative potential of the retina among species.

## 5. Current Challenges

### 5.1. Calling for Empirical Diversity and Better Demographic Representation across TFs Position Weight Matrices: The Biases That Underrate the Underdogs

How truly unbiased are the current epigenomic studies?

A simple known motifs enrichment analysis performed on ChIP-seq or ATAC-seq samples can reveal the relative representation of TF motifs by binomial or hypergeometric scoring of known deposited PWMs, that is, TF binding signatures [322]. An input or isotype control should always be performed and visually displayed to account for variations in local reads coverage, especially on low complexity sequences. Motif enrichment analysis should always display the relative representation of a given motif both in the dataset and the genome or control dataset, since the absolute percentage of the motif in the experimental dataset may not necessarily exceed its representation in the background dataset, and it should be corrected by false discovery rate.

Nonetheless, the knowledge over TF binding patterns is somehow biased towards the ones that are most frequently characterized in the literature, at a given developmental stage or physio-pathological context, through deposited ChIP-seq. In vitro assays such as protein binding microarrays [323], the systematic evolution of ligands by exponential enrichment (SELEX) [324,325], or more recently developed affinity capture methods such as multiDAP [326,327] have inherent limits: some binding sites may be undetectable due to low affinity, cofactors requirement or in vivo conditions that cannot be recapitulated [328], leading to a possible overrepresentation of a given matrix in data repositories over other matrices that are not so well characterized, prolific, or invested in.

A TF binding site may not show up as experimentally enriched for the simple fact that there is no deposited matrix associated with it; therefore it cannot be computed, not even in the background set, be it a control, untreated sample, or the genome, leading to possible false negatives.

A de novo-based approach focused on a totally agnostic identification of oligonucleotides, instead, is not limited by the accuracy and comprehensiveness of PWMs repositories, as detailed and up to date as they can be [322,329,330,331,332,333,334]. Whatever DNA sequence is present in any given sample, it can be accounted for, and it may be the most developmentally relevant one.

Multiple empirical variations of the same motif instance can be found by ATAC-seq and ChIP-seq and clustered for similarity by average linkage. Their functional recurrence in the dataset, or across species can be verified and their binding specificity tested upon permutation [75,335,336]. In a good ChIP-seq, the immunoprecipitated TF should always rank first from the de novo analysis, yet additional oligonucleotides can be identified, by similarity to known PWMs, reflecting co-occurring cofactors and steric dependencies.

Moreover, the empirical divergence of oligonucleotides from the known consensus flanking sequences may drive selective, preferential, or combinatorial recruitment of TFs onto specific promoters and co-regulated enhancers. In fact, recently evolved enhancers and cell-type specificity seem to be linked [337,338]. This may entail major implications in cell fate specification pathways and in vivo reprogramming since it would explain how TFs that display apparently redundant expression across several cell types may manage to diversify their function, leading to cellular diversity.

Even though disease-related variants seem to be preferentially associated with evolutionarily conserved enhancers [339,340,341,342], species-specific differences across TF binding patterns [341] and enhancers divergence do occur [342,343,344]. Furthermore, enhancers dispensability despite conservation [219,345,346] or functional repurposing [347], may partially explain the failures of a given TF to recapitulate the regenerative response upon injury observed in other species. Yet, none of the relevant studies have addressed this scenario [69,98,119,124,221].

To conclude, a heuristic pipeline has been proposed allowing reduction of functional redundance across TFs motifs instances populating the genome, by clustering of de novo oligonucleotides represented by ATAC-seq, followed by probabilistic assignment of such oligonucleotides to known PWM [75,348,349].

The TFs, for which binding patterns are being inferred, are then compared with stage-matched expression levels by RNA-seq, to ascertain a given TF is expressed at a given stage. ChIP-seq is then leveraged to comprehensively identify the genome-wide binding patterns of a given TF and its cognate molecular interactors.

Footprinting analysis of all endogenously recruited TFs is then led to identify actual binding sites across the genome, based on the focal depletion of the ATAC-seq reads around motifs centers, similarly to a nuclease-protection assay.

The analysis of TF signatures can be further refined by comparing footprints in control conditions and after conditional knockouts for any TF of interest, ruling out possible consensus redundancy across TF family members, thus enabling unequivocal assignment of a given binding site to a specific TF.

Functional gene ontologies can be identified from modules of coordinately accessible chromatin and related transcriptional targets, validated from conditional knockouts.

Finally, by integrating all predicted molecular interactors and co-dependencies computationally inferred by ChIP-seq motifs enrichment analysis with ATAC-seq footprinting analysis before and after loss of function for a given TF, it is possible to identify the full cohort of inter-dependent TFs that coordinate genetic programs at a given developmental stage.

Last, but not least, by adopting the PIQ metrics [179], it is possible to monitor chromatin accessibility and nucleosome occupancy around TF motif centers over time, and identify signs of nucleosome displacement by competing TFs, that is, candidate pioneer factors [174] that bind highly compacted chromatin, by making it accessible to the transcriptional machinery for the subsequent induction of gene expression.

Functional disruption of pioneer factors that interact with a given TF of interest can result from steric imbalance or epistatic de-regulation of *cis*- and *trans*-regulatory loci coordinated by such factors. Briefly, by combining the pipeline above [75] with cell lineage tracing and purification strategies, one can identify all master epigenetic regulators—as well as their steric and transcriptional dependencies—that drive the chronology of chromatin accessibility states across development, hence, the sequence of events that lead a progenitor cell towards a specific cell fate.

Developmental trajectories towards specific cell fates can be further stratified by RNA velocity pseudotime, by computing the ratio between unspliced and spliced RNA from single-cell RNA-sequencing (scRNA-seq) [350,351].

A similar concept has been recently applied on a single-cell scale, by capturing progressive chromatin opening by chromatin velocity pseudotime. Chromatin velocity is applied to scGET-seq data that enable direct and sequential profiling of open and compacted chromatin regions, an overall better resolution for the latter compared to scATAC-seq, and clonal definition by clustering of genomic copy number variants in cancer [352]. Chromatin velocity is computed as the ratio between Tn5 transposable open chromatin and heterochromatin marked by the hybrid transposase TnH, which is designed to sever closed chromatin regions while retaining affinity for the accessible ones. The TnH transposase is engineered by conjugating the Tn5 transposase to an HP1α domain targeting H3K9me3 chromatin regions. Again, modules of chromatin that acquire accessibility are then characterized by gene ontology analysis and TFs binding, by overlay with TFs PWMs and TFs expression data. To identify TFs roles in cell trajectories, the TFs dynamic score is then fitted to cell clusters by projection to latent structures regression analysis (PLS) [353].

### 5.2. Zero-Inflation, Pseudo-Replication, and Pseudo-Bulk Aggregation in Single-Cell RNA-Seq: Is Single Cell ATAC-Seq Exempt from Such Biases?

The sparsity of the signal is a common problem in single-cell RNA-seq, as it leads to zero-inflation occurrences, whereby dropout events in scRNA-seq cause many transcripts to remain undetected, resulting in high type 2 errors, hence false-negative rates [354]. A technical failure in transcript detection causes an excess of zero values compared to what is expected from a random distribution, making it difficult to distinguish technical artifacts from true biological scarcity [355].

Several technical artifacts, such as inefficient cDNA production, amplification bias, and low sequencing depth due to extreme multiplexing are known to occur [356,357]. Stochastic transcriptional bursting may also lead to zero-inflation and spurious results [358].

The zero-inflation problem is known to occur in single-cell sequencing compared to standard count distributions, such as the linear model with a negative binomial distribution, used in bulk RNA-seq [359].

Model-based analysis of single-cell transcriptomics (MAST) and dimensionality reduction methods have attempted to address these issues [357,360,361]. Expression data can be represented as continuous distribution with additional zero values and proportional relations are identified between the number of zero values and the average expression level per gene, to ultimately impute gene expression per cell, had there not been zero-inflation occurrences [362].

Recent advances in droplet scRNA-seq and mixture models have been developed to mitigate the zero-inflation problem and to reflect actual biological differences in transcripts abundance. This is accomplished by measuring discrete counts of unique molecular identifiers and assuming a negative binomial distribution [362]. Negative controls are spiked-in to mimic the RNA content of a cell, making the RNA content in each droplet identical and accounted for with a Poisson distribution, as opposed to a zero-inflated distribution. However, droplet-based single-cell sequencing data also contain intra-individual hierarchical correlation structure, possibly leading to false positives [354].

Pseudo-replication, or subsampling, can be sacrificial or simple [354]. In sacrificial pseudo-replication, experimental treatment is being performed but replicates are pooled before the analysis is conducted. In simple pseudo-replication, intra-individual samples, that is, samples obtained from the same experimental source are treated as multiple experimental replicates. Pseudo-replication seems to be pervasive in current single-cell sequencing [354].

A generalized linear mixed model and a two-part hurdle model with a random effect for the individual (MAST with RE) have recently been developed to correct for type 1 error rates, hence false positives, when performing pseudo-replication in single-cell RNA-seq showing that: (i) intra-individual correlations across cell types are consistently higher than inter-individual correlations; (ii) gene expression analysis tools that do not take into account intra-individual correlation lead to false positives (iii) as the number of correlated, cells within individual increases, performance over type 1 error rates becomes increasingly worse for all other standardized methods; (iv) power analysis of differential gene expression obtained by increasing the number of independent experimental individuals, increases the power to detect true differences [354].

Batch effect correction can be applied prior to differential expression analysis and cell-type clustering where the batches are individuals but are not exempt from high error type I rates.

Current pseudo-bulk single-cell sequencing analysis methods aggregate cell type-specific values by averaging or summing expression values [363,364,365] open chromatin regions [366,367,368] and TF footprints [124].

A feature matrix can be built upon signals originating from individual cells across genomic coordinates and the deriving, aggregated signal can then be subject to unsupervised clustering by k-means, Louvain, or hierarchical clustering [369]. The signals are then subject to dimensionality reduction and visualization by principal component analysis (PCA), t-SNE or projected with a uniform manifold learning technique for dimensionality reduction (UMAP) [370,371] to identify putative subpopulations and similarities across individual profiles [372,373,374]. The quality of clustering can then be assessed by comparison with bulk sequencing from flow-sorted cells purified by virtue of a reporter or based on the expression of known marker genes.

Simulated pseudo-aggregation of open chromatin signals obtained by synthetically mixing bulk ATAC-seq with 10X Genomics scATAC-seq in the same input set, indicate that cell type-specific peaks from rare populations are consistently underestimated by peak calling. Only 18% of cell type-specific peaks from very rare populations with 1% prevalence, or 40% cell type-specific peaks from rare populations with 5% prevalence can be detected by peak calling when treating the heterogeneous source as a synthetic bulk experiment [372]. Since these peaks would be underrepresented in a consensus, pseudo-aggregated peak set, virtually all computational algorithms are expected to fail the identification of rare populations [372]. In such a scenario, bulk sequencing from purified, flow-sorted populations would serve as an indispensable reference to resolve rare cell populations.

Furthermore, pseudo-bulk methods that aggregate signals across cells from the same individual seem to be underpowered when the number of cells per individual becomes increasingly imbalanced and when intra-individual variance exceeds inter-individual variance, which is likely to occur in a developmental context. As a result, pseudo-bulk aggregation methods across cells from the same individual underestimate within-subject variability, leading to high false-negative rates [354].

Rarefaction of the signal can be leveraged, as a normalization technique, to account for unequal sampling by downscaling samples to the same sequencing depth.

Rarefaction curves can also be leveraged to estimate the saturation point, hence, to standardize the sequencing depth and starting material for any given experimental condition.

A rarefaction analysis curve on scRNA-seq can reveal the minimum number of sequencing reads that are needed to saturate the number of valid somatic mutations identified [375], the number of unique transcripts observed, and gene coverage rates.

Saturation analysis has been traditionally adopted in ChIP-seq to identify the sequencing depth beyond which no new discoveries (binding sites) are made by a given peak caller and additional reads would only result in variations in amplitude rather than novel binding patterns [376]. Under ideal saturation conditions, binding profiles should lead to robust, reproducible results. Consistency between replicates in high-throughput sequencing analysis can be estimated by irreproducible discovery rate (IDRs) [377], to identify concordant ChiP-seq peaks, ATAC-seq open chromatin regions, or gene expression profiles. Although, deterministic processes that are consistent across replicates would rank more significant, with a lower IDR because more unlikely to be irreproducible, compared to those that occur rarely or stochastically [378], generating a sparser signal, hence limiting IDR applicability in single-cell analyses.

The recent implementation of the IDR-based framework has led to the single-cell reproducibility across donors (scRAD), in an attempt to address sacrificial pseudo-replication, overcoming confounding effects due to inter-individual variability to ultimately identify reproducible patterns of gene expression across multiple donors. This method allows to correct for batch-specific differences by measuring the reproducibility over stratified, replicate experiments, that is, multiple donors [379].

While it is not clear if, and how, current ChIP-seq and single-cell ATAC-seq experiments are being standardized [98,124], near-saturation analyses and meta-analyses of signals across multiple donors through a similar IDR framework could be leveraged to implement the method for robust, reproducible results.

Recent efforts to address the sequencing depth that is required for reproducible identification of open chromatin regions and TF footprints have, in fact, shown that the numbers of reproducible footprints gradually decline with decreasing depth and that, unlike peak calling, footprinting efficiency does not saturate but reflects the library complexities at different depths [380].

Furthermore, TF footprinting accuracy is linked to a clear distinction of footprints from the background and is contributed by the sequence biases of TF binding sites. While correcting ATAC-seq for Tn5 sequence bias did not seem to improve footprinting performance [380], keeping the replicates separate to assess reproducibility seemed to lead to more accurate footprint predictions, especially at low-sequencing depths, arguing against possible pseudo-aggregation strategies for scATAC-seq.

To conclude, these considerations warrant some caution over extreme multiplexing of scATAC-seq libraries, to ensure that proper sequencing depth per library is reached. In addition, the minimum amount of starting material, i.e., the number of cells should be empirically determined by saturation and power analysis and balanced across individuals before any pseudo-bulk aggregation attempts. Finally, independent experimental replicates should be collected and tested in power-analysis and mixed-models to address any pseudo-replication-related errors.

### 5.3. The 4D Genome: Complementing Single-Cell Sequencing with Spatiotemporal Information by Super-Resolution Imaging

The spatial organization of the genome underlies several mechanistic aspects in health, development, and disease [55,381,382,383] and recent advances in integrated spatial genomics are now enabling multimodal, simultaneous visualization of thousands of genomic loci, nascent RNA transcripts, epigenetic marks, and cooperative chromatin interactions in native conditions, at an unprecedented resolution [384,385,386,387].

Advancements in single-molecule-based, super-resolution imaging over the past decade have progressively allowed to overcome the diffraction limit and achieve resolution under the 10 nm scale for simultaneous acquisition of multiple DNA targets, relying on two key approaches. With the first approach, individually labeled probes can be transiently bound and unbound to resolve spatially overlapping images of single molecules over time and the blinking rate can be tuned by adjusting the concentration of the labeled oligos and the thermodynamic stability of the hybridized duplex. The super-resolution image is ultimately rendered from sequentially acquired, single-molecule localizations.

Another approach derives from the implementation of the photo-activated localization microscopy (PALM) [388] and utilizes small-molecule dyes, to stochastically illuminate a subset of sufficiently sparse, individual, photo-switchable fluorophores between on and off states, so that the imaged fluorophores do not substantially overlap when in the bright state, by concurrently blinking within a diffraction-limited distance [386,389]. The super-resolution image is ultimately rendered from temporally resolved emitters, from subsequent detection events.

Multiplexed super-resolution imaging of distinct DNA targets has, ever since, been achieved with the aforementioned approaches. With the former approach, libraries of short, single-stranded labeled oligonucleotides (Oligopaint probes) designed to visualize genomic regions in the range of kilobases to megabases [390] are combined with the point accumulation for imaging in nanoscale topography (DNA-PAINT). Transient binding of short fluorescently labeled, single-stranded oligonucleotides has enabled less than 20 nm resolution of synthetic DNA structures in fixed cells (OligoDNA-PAINT). In addition, Exchange-PAINT has enabled the serial imaging of multiple targets in pseudocolors with only one spectral channel [389,391].

With the latter approach derived from PALM microscopy, the iterative, stochastic activation of photo-switchable probes in 3D stochastic optical reconstruction microscopy (STORM and OligoSTORM), [389,392,393], enables axial and lateral acquisition of fluorophores to reconstruct 3D morphology of cellular structures at a nanometer scale.

STORM microscopy has been used to sequentially map and spatially locate interacting genomic loci within the cells and to reconstruct contiguous chromatin segments. Once pairwise and higher-order interactions across chromatin regions are preliminarily inferred by bulk HiC [50], diffraction-limited imaging and multiplexed STORM microscopy can be combined to probe and validate such contacts on a single-cell level, providing high sensitivity and resolution, respectively.

Multiplexed super-resolution imaging can now be leveraged for chromatin tracing, to visualize chromatin conformation across topologically associated domains (TADs), providing insights into chromatin partitioning and chromosome folding [61,394,395], since hundreds to thousands of individual loci per cell can be sequentially mapped [389,396,397,398]. Multiplexed super-resolution FISH combines structural chromatin features with their genomic coordinates in pseudocolors and compensates for the lack of sensitivity inherent to current single-cell HiC, due to the sparsity of the contacts identified [399,400,401,402,403]. Chromatin ultrastructure has been resolved at the level of single nucleosomes on fixed samples by chromatin electron tomography (ChromEMT) [404].

The recent advent of optical reconstruction of chromatin architecture methods (ORCA) [405] coupled with tiled, barcoded Oligopaint probes has ensured full coverage and uniformity of the probed chromatin region [405] and has allowed to scale up super-resolution imaging of multiple loci across tens of thousands of individual cells [386,406]. Optical reconstruction of chromatin architecture methods has enabled separation across chromatin domains [386,407,408,409], separation of recurrent chromatin blobs, and globular domains delimited by TADs boundaries that are shared within cell populations [396,397] and cell type-specific TADs boundaries [384]. DNA sequential fluorescence in situ hybridization (DNAseqFISH+) can also map 3D chromosome structures, by partially replicating the genome-wide interactions map identified by Hi-C [56], whereas cell types identified by scRNA-seq can be recapitulated by RNA seqFISH [410]. Sequential immunofluorescence with oligonucleotide-conjugated antibodies can be used to probe histone modifications, nuclear speckles, and chromatin-binding proteins, to ultimately correlate nuclear organization with transcriptional states [387].

Finally, the recently developed in situ genome sequencing (IGS) enables simultaneous base-pair sequence resolution and imaging of thousands of genomic loci within intact individual nuclei, without any prior knowledge of the sequence target [411]. The method relies on the in situ transposition of fixed samples, where the Tn5 transposase is added to randomly incorporate DNA sequencing adaptors upon fixation, preserving all genomic fragments in their native spatial position. Tagmented fragments are then circularized by ligation mediated by DNA hairpins with unique molecular identifiers (UMI) and the deriving templates are amplified by rolling circle amplification. UMIs are first imaged by immunostaining and deconvolved, then subject to further in situ amplification for library generation. The libraries are ultimately dissociated and sequenced ex situ on a regular Illumina platform. Upon sequencing, deconvolved in situ fluorescent locations are mapped to the corresponding UMI tagged genomic readout, enabling spatial assignment of the sequencing output for every single cell. This method has allowed to assign novel sequencing reads to the nuclear lamina, centromeres, and nucleolar bodies.

Last, but not least, optical sectioning capabilities and axial resolution have been recently increased by 3D interferometric lattice light-sheet microscopy (3D-iLLS) [412] and nanoscale chromatin imaging and analysis (nano-ChIA) [413], enabling unprecedented long-term, spatio-temporally resolved and label-free three-dimensional acquisition of subcellular structures in live biological specimens, ranging between 20 and 300 nm.

While a thorough presentation of the current imaging techniques eludes the scope of this review, super-resolution imaging will undoubtedly advance the mechanistic understanding of gene regulatory networks and functional epigenetic states behind cell fate determination, through direct visualization of chromatin compartmentalization and topology in space and time.

## 6. Conclusions and Future Perspectives

### Harnessing the Full Potential of Next-Generation Sequencing: Resolution vs. Informativity

The advent of single-cell sequencing over the past decade has revolutionized several fields spanning across molecular and developmental biology by enabling stratification of the cellular, transcriptomic, and epigenetic heterogeneity in cell fate specification and within cell populations, leading to the taxonomic identification of novel cell types and to the inference of cell fate probabilities and developmental trajectories across pseudo-times [62,64,65,66,67,68,69,70,71,72,73,74,128,185,350,414,415,416,417,418,419,420,421,422,423,424].

To what extent can we push the boundaries of informativity of next-generation sequencing?

A PubMed search for “single-cell differential expression” returned 9049 results published since 2016. Moreover, 7610 articles were published related to “chromatin accessibility” in the same time frame, of which 812 were led on by single-cell sequencing, while 659 on single-cell chromatin conformation.

Notwithstanding, the spurious contribution of heterogeneous cell types from whole-cell populations can be circumvented by several purification strategies prior to bulk sequencing, such as immunolabeling, flow sorting [75,425], or through in vivo biotin labeling of the nuclear envelope, followed by affinity purification of tagged nuclei (INTACT) [426], leading to a reasonably pure cell population by virtue of a reporter. Conversely, rarefaction issues are known to occur in single-cell sequencing due to the sparsity of the signal generated by few cells, the noise, and the underrepresentation of rare amplicons, which is hard to obviate.

This is particularly relevant for footprinting studies where the inflection of the reads around TF binding sites relies on a population-averaged signal. scATAC-seq data are inherently sparse [372]. Infrequent TF binding in rare cell types, short resident TF binding, or cell type-specific peaks from very rare cell populations may not necessarily be reflected in features matrices from pseudo-aggregated single-cell ATAC-seq signals, especially if under-sampling occurs, leading to possible false negatives, making the classic bulk ATAC-seq from whole-cell populations still a more informative, and an overall more desirable option.

Theoretically, to benchmark a truly representative dataset, one may want to establish minimum sampling requirements and iterate single-cell sequencing data collection, by progressively scaling up the sample size for power analysis and saturation until no new peaks and footprints are being discovered. Since the proportion of rare cell types may differ, depending on the developmental context, this control would have to be repeated for every single experimental condition and developmental time point, resulting in a time-consuming experimental setup and substantial financial burden.

Single-cell sequencing also entails considerable data workload, requires extensive storage and processing capacity. Computationally intensive programs and mixed models-based analysis that are iteratively performed on thousands of samples may limit the applicability of the FAIR principles of findability, accessibility, and interoperability across platforms and users, underscoring the need of pipelines for better data harmonization.

Super-resolution imaging techniques could ultimately complement genome-wide sequencing techniques on a single-cell level.

Finally, single-cell sequencing can graciously supplement the lack of hypothesis testing while sustaining the scientific prolificacy over time and has, in fact, raised exponentially over the past decade. Nonetheless, the above considerations should warrant some caution before resorting to its default usage and to a programmed obsolescence of bulk sequencing, calling for a more rational experimental design to avoid uninformative redundancies [427], and for a more equitable allocation of the funding resources [428,429].

## Data Availability

C.Z. generated the following datasets during her previous post-doctoral research, from 2011 to 2017. Subsequently, the datasets were made available at the NIH NCBI GEO data repository as GSE99287 [226], GSE99818 [75,425] and GSE118880 [185].

## References

[1] Fuhrmann S. (2010). Eye morphogenesis and patterning of the optic vesicle. Curr. Top. Dev. Biol..

[2] Giger F.A., Houart C. (2018). The Birth of the Eye Vesicle: When Fate Decision Equals Morphogenesis. Front. Neurosci..

[3] Miesfeld J.B., Brown N.L. (2019). Eye organogenesis: A hierarchical view of ocular development. Curr. Top. Dev. Biol..

[4] Meunier D., Lambiotte R., Bullmore E. (2010). Modular and Hierarchically Modular Organization of Brain Networks. Front. Neurosci..

[5] Guy J., Staiger J.F. (2017). The Functioning of a Cortex without Layers. Front. Neuroanat..

[6] Hoon M., Okawa H., Della Santina L., Wong R.O. (2014). Functional architecture of the retina: Development and disease. Prog. Retin. Eye Res..

[7] Amini R., Rocha-Martins M., Norden C. (2017). Neuronal Migration and Lamination in the Vertebrate Retina. Front. Neurosci..

[8] Donovan S.L., Dyer M.A. (2005). Regulation of proliferation during central nervous system development. Semin. Cell Dev. Biol..

[9] Kohwi M., Doe C.Q. (2013). Temporal fate specification and neural progenitor competence during development. Nat. Rev. Neurosci..

[10] Yoles E., Hauben E., Palgi O., Agranov E., Gothilf A., Cohen A., Kuchroo V., Cohen I.R., Weiner H., Schwartz M. (2001). Protective autoimmunity is a physiological response to CNS trauma. J. Neurosci..

[11] Benowitz L., Yin Y. (2008). Rewiring the injured CNS: Lessons from the optic nerve. Exp. Neurol..

[12] Gadani S.P., Walsh J.T., Lukens J.R., Kipnis J. (2015). Dealing with Danger in the CNS: The Response of the Immune System to Injury. Neuron.

[13] Simon D.W., McGeachy M.J., Bayir H., Clark R.S., Loane D.J., Kochanek P.M. (2017). The far-reaching scope of neuroinflammation after traumatic brain injury. Nat. Rev. Neurol..

[14] Chen M., Luo C., Zhao J., Devarajan G., Xu H. (2019). Immune regulation in the aging retina. Prog. Retin. Eye Res..

[15] Stepp M.A., Menko A.S. (2021). Immune responses to injury and their links to eye disease. Transl. Res..

[16] London A., Benhar I., Schwartz M. (2013). The retina as a window to the brain—From eye research to CNS disorders. Nat. Rev. Neurol..

[17] Nowacka B., Lubiński W., Honczarenko K., Potemkowski A., Safranow K. (2014). Ophthalmological features of Parkinson disease. Med. Sci. Monit..

[18] Hirose A., Hirose A., Katagiri S., Hayashi T., Matsuura T., Nagai N., Fujinami K., Iwata T., Tsunoda K. (2021). Progress of macular atrophy during 30 months’ follow-up in a patient with spinocerebellar ataxia type1 (SCA1). Doc. Ophthalmol..

[19] Liao C., Xu J., Chen Y., Ip N.Y. (2021). Retinal Dysfunction in Alzheimer’s Disease and Implications for Biomarkers. Biomolecules.

[20] Dhawan P.S., Leong D., Tapsell L., Starling A.J., Galetta S.L., Balcer L.J., Overall T.L., Adler J.S., Halker-Singh R.B., Vargas B.B. (2017). King-Devick Test identifies real-time concussion and asymptomatic concussion in youth athletes. Neurol. Clin. Pract..

[21] Fisher J.B., Jacobs D.A., Markowitz C.E., Galetta S.L., Volpe N.J., Nano-Schiavi M.L., Baier M.L., Frohman E.M., Winslow H., Frohman T.C. (2006). Relation of visual function to retinal nerve fiber layer thickness in multiple sclerosis. Ophthalmology.

[22] Talman L.S., Bisker E.R., Sackel D.J., Long D.A., Galetta K.M., Ratchford J.N., Lile D.J., Farrell S.K., Loguidice M.J., Remington G. (2010). Longitudinal study of vision and retinal nerve fiber layer thickness in multiple sclerosis. Ann. Neurol..

[23] Petzold A., de Boer J.F., Schippling S., Vermersch P., Kardon R., Green A., Calabresi P.A., Polman C. (2010). Optical coherence tomography in multiple sclerosis: A systematic review and meta-analysis. Lancet Neurol..

[24] Mailankody P., Battu R., Khanna A., Lenka A., Yadav R., Pal P.K. (2015). Optical coherence tomography as a tool to evaluate retinal changes in Parkinson’s disease. Parkinsonism Relat. Disord..

[25] Ahn J., Lee J.Y., Kim T.W., Yoon E.J., Oh S., Kim Y.K., Kim J.M., Woo S.J., Kim K.W., Jeon B. (2018). Retinal thinning associates with nigral dopaminergic loss in de novo Parkinson disease. Neurology.

[26] Abd Hamid M.R., Wan Hitam W.H., Abd Halim S. (2021). Retinal Nerve Fiber Layer and Macular Thickness in Parkinson’s Disease Patients. Cureus.

[27] Balcer L.J., Raynowska J., Nolan R., Galetta S.L., Kapoor R., Benedict R., Phillips G., LaRocca N., Hudson L., Rudick R. (2017). Validity of low-contrast letter acuity as a visual performance outcome measure for multiple sclerosis. Mult. Scler. J..

[28] Seay M., Akhand O., Galetta M.S., Cobbs L., Hasanaj L., Amorapanth P., Rizzo J.R., Nolan R., Serrano L., Rucker J.C. (2018). Mobile Universal Lexicon Evaluation System (MULES) in MS: Evaluation of a new visual test of rapid picture naming. J. Neurol. Sci..

[29] Conway J., Ilardi M., Gonzalez C., Dahan N., Fallon S., Moehringer N., Hasanaj L., Joseph B., Serrano L., Rizzo J.R. (2020). Rapid picture naming in Parkinson’s disease using the Mobile Universal Lexicon Evaluation System (MULES). J. Neurol. Sci..

[30] O’Bryhim B.E., Apte R.S., Kung N., Coble D., Van Stavern G.P. (2018). Association of Preclinical Alzheimer Disease with Optical Coherence Tomographic Angiography Findings. JAMA Ophthalmol..

[31] Kleerekooper I., Houston S., Dubis A.M., Trip S.A., Petzold A. (2020). Optical Coherence Tomography Angiography (OCTA) in Multiple Sclerosis and Neuromyelitis Optica Spectrum Disorder. Front. Neurol..

[32] Almonte M.T., Capellan P., Yap T.E., Cordeiro M.F. (2020). Retinal correlates of psychiatric disorders. Ther. Adv. Chronic Dis..

[33] Silverstein S.M., Fradkin S.I., Demmin D.L. (2020). Schizophrenia and the retina: Towards a 2020 perspective. Schizophr. Res..

[34] Arsenault E., Lavigne A.A., Mansouri S., Gagne A.M., Francis K., Bittar T.P., Quessy F., Abdallah K., Barbeau A., Hebert M. (2021). Sex-Specific Retinal Anomalies Induced by Chronic Social Defeat Stress in Mice. Front. Behav. Neurosci..

[35] Turner D.L., Cepko C.L. (1987). A common progenitor for neurons and glia persists in rat retina late in development. Nature.

[36] Ohsawa R., Kageyama R. (2008). Regulation of retinal cell fate specification by multiple TFs. Brain Res..

[37] Bassett E.A., Wallace V.A. (2012). Cell fate determination in the vertebrate retina. Trends Neurosci..

[38] Mo A., Mukamel E.A., Davis F.P., Luo C., Henry G.L., Picard S., Urich M.A., Nery J.R., Sejnowski T.J., Lister R. (2015). Epigenomic Signatures of Neuronal Diversity in the Mammalian Brain. Neuron.

[39] Young R.W. (1985). Cell differentiation in the retina of the mouse. Anat. Rec..

[40] Livesey F.J., Cepko C.L. (2001). Vertebrate neural cell-fate determination: Lessons from the retina. Nat. Rev. Neurosci..

[41] Rapaport D.H., Wong L.L., Wood E.D., Yasumura D., LaVail M.M. (2004). Timing and topography of cell genesis in the rat retina. J. Comp. Neurol..

[42] Dyer M.A., Martins R., da Silva Filho M., Muniz J.A., Silveira L.C., Cepko C.L., Finlay B.L. (2009). Developmental sources of conservation and variation in the evolution of the primate eye. Proc. Natl. Acad. Sci. USA.

[43] He J., Zhang G., Almeida A.D., Cayouette M., Simons B.D., Harris W.A. (2012). How variable clones build an invariant retina. Neuron.

[44] Cepko C. (2014). Intrinsically different retinal progenitor cells produce specific types of progeny. Nat. Rev. Neurosci..

[45] Rossi A.M., Fernandes V.M., Desplan C. (2017). Timing temporal transitions during brain development. Curr. Opin. Neurobiol..

[46] Cremer T., Kurz A., Zirbel R., Dietzel S., Rinke B., Schrock E., Speicher M.R., Mathieu U., Jauch A., Emmerich P. (1993). Role of chromosome territories in the functional compartmentalization of the cell nucleus. Role of chromosome territories in the functional compartmentalization of the cell nucleus. Cold Spring Harb. Symp. Quant. Biol..

[47] Croft J.A., Bridger J.M., Boyle S., Perry P., Teague P., Bickmore W.A. (1999). Differences in the localization and morphology of chromosomes in the human nucleus. J. Cell Biol..

[48] Tanabe H., Muller S., Neusser M., von Hase J., Calcagno E., Cremer M., Solovei I., Cremer C., Cremer T. (2002). Evolutionary conservation of chromosome territory arrangements in cell nuclei from higher primates. Proc. Natl. Acad. Sci. USA.

[49] Misteli T. (2007). Beyond the sequence: Cellular organization of genome function. Cell.

[50] Lieberman-Aiden E., van Berkum N.L., Williams L., Imakaev M., Ragoczy T., Telling A., Amit I., Lajoie B.R., Sabo P.J., Dorschner M.O. (2009). Comprehensive mapping of long-range interactions reveals folding principles of the human genome. Science.

[51] Smallwood A., Ren B. (2013). Genome organization and long-range regulation of gene expression by enhancers. Curr. Opin. Cell Biol..

[52] Rao S.S., Huntley M.H., Durand N.C., Stamenova E.K., Bochkov I.D., Robinson J.T., Sanborn A.L., Machol I., Omer A.D., Lander E.S. (2014). A 3D map of the human genome at kilobase resolution reveals principles of chromatin looping. Cell.

[53] Bianco S., Lupianez D.G., Chiariello A.M., Annunziatella C., Kraft K., Schopflin R., Wittler L., Andrey G., Vingron M., Pombo A. (2018). Polymer physics predicts the effects of structural variants on chromatin architecture. Nat. Genet..

[54] Jerković I., Cavalli G. (2021). Understanding 3D genome organization by multidisciplinary methods. Nat. Rev. Mol. Cell Biol..

[55] Lupianez D.G., Kraft K., Heinrich V., Krawitz P., Brancati F., Klopocki E., Horn D., Kayserili H., Opitz J.M., Laxova R. (2015). Disruptions of topological chromatin domains cause pathogenic rewiring of gene-enhancer interactions. Cell.

[56] Dixon J.R., Selvaraj S., Yue F., Kim A., Li Y., Shen Y., Hu M., Liu J.S., Ren B. (2012). Topological domains in mammalian genomes identified by analysis of chromatin interactions. Nature.

[57] Hnisz D., Abraham B.J., Lee T.I., Lau A., Saint-Andre V., Sigova A.A., Hoke H.A., Young R.A. (2013). Super-enhancers in the control of cell identity and disease. Cell.

[58] Filippova D., Patro R., Duggal G., Kingsford C. (2014). Identification of alternative topological domains in chromatin. Algorithms Mol. Biol..

[59] Won H., de la Torre-Ubieta L., Stein J.L., Parikshak N.N., Huang J., Opland C.K., Gandal M.J., Sutton G.J., Hormozdiari F., Lu D. (2016). Chromosome conformation elucidates regulatory relationships in developing human brain. Nature.

[60] Freire-Pritchett P., Schoenfelder S., Varnai C., Wingett S.W., Cairns J., Collier A.J., Garcia-Vilchez R., Furlan-Magaril M., Osborne C.S., Fraser P. (2017). Global reorganisation of cis-regulatory units upon lineage commitment of human embryonic stem cells. eLife.

[61] Dekker J., Belmont A.S., Guttman M., Leshyk V.O., Lis J.T., Lomvardas S., Mirny L.A., O’Shea C.C., Park P.J., Ren B. (2017). The 4D nucleome project. Nature.

[62] Macosko E.Z., Basu A., Satija R., Nemesh J., Shekhar K., Goldman M., Tirosh I., Bialas A.R., Kamitaki N., Martersteck E.M. (2015). Highly Parallel Genome-wide Expression Profiling of Individual Cells Using Nanoliter Droplets. Cell.

[63] Hoshino A., Ratnapriya R., Brooks M.J., Chaitankar V., Wilken M.S., Zhang C., Starostik M.R., Gieser L., La Torre A., Nishio M. (2017). Molecular Anatomy of the Developing Human Retina. Dev. Cell.

[64] Rheaume B.A., Jereen A., Bolisetty M., Sajid M.S., Yang Y., Renna K., Sun L., Robson P., Trakhtenberg E.F. (2018). Single cell transcriptome profiling of retinal ganglion cells identifies cellular subtypes. Nat. Commun..

[65] Peng Y.R., Shekhar K., Yan W., Herrmann D., Sappington A., Bryman G.S., van Zyl T., Do M.T.H., Regev A., Sanes J.R. (2019). Molecular Classification and Comparative Taxonomics of Foveal and Peripheral Cells in Primate Retina. Cell.

[66] Laboissonniere L.A., Goetz J.J., Martin G.M., Bi R., Lund T.J.S., Ellson L., Lynch M.R., Mooney B., Wickham H., Liu P. (2019). Molecular signatures of retinal ganglion cells revealed through single cell profiling. Sci. Rep..

[67] Tran N.M., Shekhar K., Whitney I.E., Jacobi A., Benhar I., Hong G., Yan W., Adiconis X., Arnold M.E., Lee J.M. (2019). Single-Cell Profiles of Retinal Ganglion Cells Differing in Resilience to Injury Reveal Neuroprotective Genes. Neuron.

[68] Kolsch Y., Hahn J., Sappington A., Stemmer M., Fernandes A.M., Helmbrecht T.O., Lele S., Butrus S., Laurell E., Arnold-Ammer I. (2021). Molecular classification of zebrafish retinal ganglion cells links genes to cell types to behavior. Neuron.

[69] Xie H., Zhang W., Zhang M., Akhtar T., Li Y., Yi W., Sun X., Zuo Z., Wei M., Fang X. (2020). Chromatin accessibility analysis reveals regulatory dynamics of developing human retina and hiPSC-derived retinal organoids. Sci. Adv..

[70] Cherry T.J., Yang M.G., Harmin D.A., Tao P., Timms A.E., Bauwens M., Allikmets R., Jones E.M., Chen R., De Baere E. (2020). Mapping the cis-regulatory architecture of the human retina reveals noncoding genetic variation in disease. Proc. Natl. Acad. Sci. USA.

[71] Sridhar A., Hoshino A., Finkbeiner C.R., Chitsazan A., Dai L., Haugan A.K., Eschenbacher K.M., Jackson D.L., Trapnell C., Bermingham-McDonogh O. (2020). Single-Cell Transcriptomic Comparison of Human Fetal Retina, hPSC-Derived Retinal Organoids, and Long-Term Retinal Cultures. Cell Rep..

[72] Cowan C.S., Renner M., De Gennaro M., Gross-Scherf B., Goldblum D., Hou Y., Munz M., Rodrigues T.M., Krol J., Szikra T. (2020). Cell Types of the Human Retina and Its Organoids at Single-Cell Resolution. Cell.

[73] Armand E.J., Li J., Xie F., Luo C., Mukamel E.A. (2021). Single-Cell Sequencing of Brain Cell Transcriptomes and Epigenomes. Neuron.

[74] Ozel M.N., Simon F., Jafari S., Holguera I., Chen Y.C., Benhra N., El-Danaf R.N., Kapuralin K., Malin J.A., Konstantinides N. (2021). Neuronal diversity and convergence in a visual system developmental atlas. Nature.

[75] Zibetti C., Liu S., Wan J., Qian J., Blackshaw S. (2019). Epigenomic profiling of retinal progenitors reveals LHX2 is required for developmental regulation of open chromatin. Commun. Biol..

[76] Li M., Jia C., Jia C., Kazmierkiewicz K.L., Bowman A.S., Tian L., Liu Y., Gupta N.A., Gudiseva H.V., Yee S.S. (2014). Comprehensive analysis of gene expression in human retina and supporting tissues. Hum. Mol. Genet..

[77] Crawford G.E., Holt I.E., Whittle J., Webb B.D., Tai D., Davis S., Margulies E.H., Chen Y., Bernat J.A., Ginsburg D. (2006). Genome-wide mapping of DNase hypersensitive sites using massively parallel signature sequencing (MPSS). Genome Res..

[78] Buenrostro J.D., Giresi P.G., Zaba L.C., Chang H.Y., Greenleaf W.J. (2013). Transposition of native chromatin for fast and sensitive epigenomic profiling of open chromatin, DNA-binding proteins and nucleosome position. Nat. Methods.

[79] Ziller M.J., Edri R., Yaffe Y., Donaghey J., Pop R., Mallard W., Issner R., Gifford C.A., Goren A., Xing J. (2015). Dissecting neural differentiation regulatory networks through epigenetic footprinting. Nature.

[80] Dekker J., Rippe K., Dekker M., Kleckner N. (2002). Capturing chromosome conformation. Science.

[81] Dostie J., Richmond T.A., Arnaout R.A., Selzer R.R., Lee W.L., Honan T.A., Rubio E.D., Krumm A., Lamb J., Nusbaum C. (2006). Chromosome Conformation Capture Carbon Copy (5C): A massively parallel solution for mapping interactions between genomic elements. Genome Res..

[82] Yardimci G.G., Noble W.S. (2017). Software tools for visualizing Hi-C data. Genome Biol..

[83] Aldiri I., Xu B., Wang L., Chen X., Hiler D., Griffiths L., Valentine M., Shirinifard A., Thiagarajan S., Sablauer A. (2017). The Dynamic Epigenetic Landscape of the Retina During Development, Reprogramming, and Tumorigenesis. Neuron.

[84] Ernst J., Kellis M. (2012). ChromHMM: Automating chromatin-state discovery and characterization. Nat. Methods.

[85] Creyghton M.P., Cheng A.W., Welstead G.G., Kooistra T., Carey B.W., Steine E.J., Hanna J., Lodato M.A., Frampton G.M., Sharp P.A. (2010). Histone H3K27ac separates active from poised enhancers and predicts developmental state. Proc. Natl. Acad. Sci. USA.

[86] Visel A., Blow M.J., Li Z., Zhang T., Akiyama J.A., Holt A., Plajzer-Frick I., Shoukry M., Wright C., Chen F. (2009). ChIP-seq accurately predicts tissue-specific activity of enhancers. Nature.

[87] Brzezinski J.A., Kim E.J., Johnson J.E., Reh T.A. (2011). Ascl1 expression defines a subpopulation of lineage-restricted progenitors in the mammalian retina. Development.

[88] Baden T., Euler T., Berens P. (2020). Understanding the retinal basis of vision across species. Nat. Rev. Neurosci..

[89] Todd L., Reh T.A. (2021). Comparative Biology of Vertebrate Retinal Regeneration: Restoration of Vision through Cellular Reprogramming. Cold Spring Harb. Perspect. Biol..

[90] Land M.F. (1972). The physics and biology of animal reflectors. Prog. Biophys. Mol. Biol..

[91] Kreysing M., Pusch R., Haverkate D., Landsberger M., Engelmann J., Ruiter J., Mora-Ferrer C., Ulbricht E., Grosche J., Franze K. (2012). Photonic crystal light collectors in fish retina improve vision in turbid water. Science.

[92] Blaszczak Z., Kreysing M., Guck J. (2014). Direct observation of light focusing by single photoreceptor cell nuclei. Opt. Express.

[93] Subramanian K., Weigert M., Borsch O., Petzold H., Garcia-Ulloa A., Myers E.W., Ader M., Solovei I., Kreysing M. (2019). Rod nuclear architecture determines contrast transmission of the retina and behavioral sensitivity in mice. eLife.

[94] Subramanian K., Petzold H., Seelbinder B., Hersemann L., Nusslein I., Kreysing M. (2021). Optical plasticity of mammalian cells. J. Biophotonics.

[95] Solovei I., Kreysing M., Lanctot C., Kosem S., Peichl L., Cremer T., Guck J., Joffe B. (2009). Nuclear architecture of rod photoreceptor cells adapts to vision in mammalian evolution. Cell.

[96] Kreysing M., Boyde L., Guck J., Chalut K.J. (2010). Physical insight into light scattering by photoreceptor cell nuclei. Opt. Lett..

[97] Wang L., Hiler D., Xu B., AlDiri I., Chen X., Zhou X., Griffiths L., Valentine M., Shirinifard A., Sablauer A. (2018). Retinal Cell Type DNA Methylation and Histone Modifications Predict Reprogramming Efficiency and Retinogenesis in 3D Organoid Cultures. Cell Rep..

[98] Norrie J.L., Lupo M.S., Xu B., Al Diri I., Valentine M., Putnam D., Griffiths L., Zhang J., Johnson D., Easton J. (2019). Nucleome Dynamics during Retinal Development. Neuron.

[99] Chen X., Pappo A., Dyer M.A. (2015). Pediatric solid tumor genomics and developmental pliancy. Oncogene.

[100] Denny S.K., Yang D., Chuang C.H., Brady J.J., Lim J.S., Gruner B.M., Chiou S.H., Schep A.N., Baral J., Hamard C. (2016). Nfib Promotes Metastasis through a Widespread Increase in Chromatin Accessibility. Cell.

[101] Grabowska M.M., Kelly S.M., Reese A.L., Cates J.M., Case T.C., Zhang J., DeGraff D.J., Strand D.W., Miller N.L., Clark P.E. (2016). Nfib Regulates Transcriptional Networks That Control the Development of Prostatic Hyperplasia. Endocrinology.

[102] Aldiri I., Valentine M., Xu B., Putnam D., Griffiths L., Lupo M., Norrie J., Zhang J., Johnson D., Easton J. (2018). The Nucleome of Developing Murine Rod Photoreceptors. bioRxiv.

[103] Stolt C.C., Lommes P., Sock E., Chaboissier M.C., Schedl A., Wegner M. (2003). The Sox9 TF determines glial fate choice in the developing spinal cord. Genes Dev..

[104] Elliott J., Jolicoeur C., Ramamurthy V., Cayouette M. (2008). Ikaros confers early temporal competence to mouse retinal progenitor cells. Neuron.

[105] Alsio J.M., Tarchini B., Cayouette M., Livesey F.J. (2013). Ikaros promotes early-born neuronal fates in the cerebral cortex. Proc. Natl. Acad. Sci. USA.

[106] La Torre A., Georgi S., Reh T.A. (2013). Conserved microRNA pathway regulates developmental timing of retinal neurogenesis. Proc. Natl. Acad. Sci. USA.

[107] Saurat N., Andersson T., Vasistha N.A., Molnar Z., Livesey F.J. (2013). Dicer is required for neural stem cell multipotency and lineage progression during cerebral cortex development. Neural Dev..

[108] Wang S., Sengel C., Emerson M.M., Cepko C.L. (2014). A gene regulatory network controls the binary fate decision of rod and bipolar cells in the vertebrate retina. Dev. Cell.

[109] Wang S., Cepko C.L. (2016). Photoreceptor Fate Determination in the Vertebrate Retina. Investig. Opthalmol. Vis. Sci..

[110] Stenkamp D.L. (2015). Development of the Vertebrate Eye and Retina. Prog. Mol. Biol. Transl. Sci..

[111] Zhang X.M., Yang X.J. (2001). Regulation of retinal ganglion cell production by Sonic hedgehog. Development.

[112] Rodriguez M., Choi J., Park S., Sockanathan S. (2012). Gde2 regulates cortical neuronal identity by controlling the timing of cortical progenitor differentiation. Development.

[113] Vetter M.L., Brown N.L. (2001). The role of basic helix-loop-helix genes in vertebrate retinogenesis. Semin. Cell Dev. Biol..

[114] Kageyama R., Ohtsuka T., Hatakeyama J., Ohsawa R. (2005). Roles of bHLH genes in neural stem cell differentiation. Exp. Cell Res..

[115] Masland R.H. (2012). The neuronal organization of the retina. Neuron.

[116] Boije H., MacDonald R.B., Harris W.A. (2014). Reconciling competence and transcriptional hierarchies with stochasticity in retinal lineages. Curr. Opin. Neurobiol..

[117] Zhang J., Taylor R.J., La Torre A., Wilken M.S., Cox K.E., Reh T.A., Vetter M.L. (2015). Ezh2 maintains retinal progenitor proliferation, transcriptional integrity, and the timing of late differentiation. Dev. Biol..

[118] Mellough C.B., Bauer R., Collin J., Dorgau B., Zerti D., Dolan D.W.P., Jones C.M., Izuogu O.G., Yu M., Hallam D. (2019). An integrated transcriptional analysis of the developing human retina. Development.

[119] VandenBosch L.S., Wohl S.G., Wilken M.S., Hooper M., Finkbeiner C., Cox K., Chipman L., Reh T.A. (2020). Developmental changes in the accessible chromatin, transcriptome and Ascl1-binding correlate with the loss in Muller Glial regenerative potential. Sci. Rep..

[120] Corbo J.C., Lawrence K.A., Karlstetter M., Myers C.A., Abdelaziz M., Dirkes W., Weigelt K., Seifert M., Benes V., Fritsche L.G. (2010). CRX ChIP-seq reveals the cis-regulatory architecture of mouse photoreceptors. Genome Res..

[121] Ruzycki P.A., Zhang X., Chen S. (2018). CRX directs photoreceptor differentiation by accelerating chromatin remodeling at specific target sites. Epigenet. Chromatin.

[122] Samuel A., Housset M., Fant B., Lamonerie T. (2014). Otx2 ChIP-seq reveals unique and redundant functions in the mature mouse retina. PLoS ONE.

[123] Swaroop A., Kim D., Forrest D. (2010). Transcriptional regulation of photoreceptor development and homeostasis in the mammalian retina. Nat. Rev. Neurosci..

[124] Lyu P., Hoang T., Santiago C.P., Thomas E.D., Timms A.E., Appel H., Gimmen M., Le N., Jiang L., Kim D.W. (2021). Gene regulatory networks controlling temporal patterning, neurogenesis, and cell-fate specification in mammalian retina. Cell Rep..

[125] Andzelm M.M., Cherry T.J., Harmin D.A., Boeke A.C., Lee C., Hemberg M., Pawlyk B., Malik A.N., Flavell S.W., Sandberg M.A. (2015). MEF2D drives photoreceptor development through a genome-wide competition for tissue-specific enhancers. Neuron.

[126] Konstantinides N., Kapuralin K., Fadil C., Barboza L., Satija R., Desplan C. (2018). Phenotypic Convergence: Distinct TFs Regulate Common Terminal Features. Cell.

[127] Clark B.S., Stein-O’Brien G.L., Shiau F., Cannon G.H., Davis-Marcisak E., Sherman T., Santiago C.P., Hoang T.V., Rajaii F., James-Esposito R.E. (2019). Single-Cell RNA-Seq Analysis of Retinal Development Identifies NFI Factors as Regulating Mitotic Exit and Late-Born Cell Specification. Neuron.

[128] Lu Y., Shiau F., Yi W., Lu S., Wu Q., Pearson J.D., Kallman A., Zhong S., Hoang T., Zuo Z. (2020). Single-Cell Analysis of Human Retina Identifies Evolutionarily Conserved and Species-Specific Mechanisms Controlling Development. Dev. Cell.

[129] Brzezinski J.A., Park K.U., Reh T.A. (2013). Blimp1 (Prdm1) prevents re-specification of photoreceptors into retinal bipolar cells by restricting competence. Dev. Biol..

[130] Hou P.S., Chuang C.Y., Kao C.F., Chou S.J., Stone L., Ho H.N., Chien C.L., Kuo H.C. (2013). LHX2 regulates the neural differentiation of human embryonic stem cells via transcriptional modulation of PAX6 and CER1. Nucleic Acids Res..

[131] Gordon P.J., Yun S., Clark A.M., Monuki E.S., Murtaugh L.C., Levine E.M. (2013). Lhx2 balances progenitor maintenance with neurogenic output and promotes competence state progression in the developing retina. J. Neurosci..

[132] de Melo J., Zibetti C., Clark B.S., Hwang W., Miranda-Angulo A.L., Qian J., Blackshaw S. (2016). Lhx2 Is an Essential Factor for Retinal Gliogenesis and Notch Signaling. J. Neurosci..

[133] de Melo J., Clark B.S., Blackshaw S. (2016). Multiple intrinsic factors act in concert with Lhx2 to direct retinal gliogenesis. Sci. Rep..

[134] Roy A., de Melo J., Chaturvedi D., Thein T., Cabrera-Socorro A., Houart C., Meyer G., Blackshaw S., Tole S. (2013). LHX2 is necessary for the maintenance of optic identity and for the progression of optic morphogenesis. J. Neurosci..

[135] Mangale V.S., Hirokawa K.E., Satyaki P.R., Gokulchandran N., Chikbire S., Subramanian L., Shetty A.S., Martynoga B., Paul J., Mai M.V. (2008). Lhx2 selector activity specifies cortical identity and suppresses hippocampal organizer fate. Science.

[136] Chou S.J., Perez-Garcia C.G., Kroll T.T., O’Leary D.D. (2009). Lhx2 specifies regional fate in Emx1 lineage of telencephalic progenitors generating cerebral cortex. Nat. Neurosci..

[137] Subramanian L., Sarkar A., Shetty A.S., Muralidharan B., Padmanabhan H., Piper M., Monuki E.S., Bach I., Gronostajski R.M., Richards L.J. (2011). TF Lhx2 is necessary and sufficient to suppress astro-gliogenesis and promote neurogenesis in the developing hippocampus. Proc. Natl. Acad. Sci. USA.

[138] Chou S.J., O’Leary D.D. (2013). Role for Lhx2 in corticogenesis through regulation of progenitor differentiation. Mol. Cell. Neurosci..

[139] Chinn G.A., Hirokawa K.E., Chuang T.M., Urbina C., Patel F., Fong J., Funatsu N., Monuki E.S. (2015). Agenesis of the Corpus Callosum Due to Defective Glial Wedge Formation in Lhx2 Mutant Mice. Cereb. Cortex.

[140] Porter F.D., Drago J., Xu Y., Cheema S.S., Wassif C., Huang S.P., Lee E., Grinberg A., Massalas J.S., Bodine D. (1997). Lhx2, a LIM homeobox gene, is required for eye, forebrain, and definitive erythrocyte development. Development.

[141] Tetreault N., Champagne M.P., Bernier G. (2009). The LIM homeobox TF Lhx2 is required to specify the retina field and synergistically cooperates with Pax6 for Six6 transactivation. Dev. Biol..

[142] Monuki E.S., Porter F.D., Walsh C.A. (2001). Patterning of the dorsal telencephalon and cerebral cortex by a roof plate-Lhx2 pathway. Neuron.

[143] Zhong X., Gutierrez C., Xue T., Hampton C., Vergara M.N., Cao L.H., Peters A., Park T.S., Zambidis E.T., Meyer J.S. (2014). Generation of three-dimensional retinal tissue with functional photoreceptors from human iPSCs. Nat. Commun..

[144] Phillips M.J., Perez E.T., Martin J.M., Reshel S.T., Wallace K.A., Capowski E.E., Singh R., Wright L.S., Clark E.M., Barney P.M. (2014). Modeling human retinal development with patient-specific induced pluripotent stem cells reveals multiple roles for visual system homeobox 2. Stem Cells.

[145] Capowski E.E., Simonett J.M., Clark E.M., Wright L.S., Howden S.E., Wallace K.A., Petelinsek A.M., Pinilla I., Phillips M.J., Meyer J.S. (2014). Loss of MITF expression during human embryonic stem cell differentiation disrupts retinal pigment epithelium development and optic vesicle cell proliferation. Hum. Mol. Genet..

[146] Raviv S., Bharti K., Rencus-Lazar S., Cohen-Tayar Y., Schyr R., Evantal N., Meshorer E., Zilberberg A., Idelson M., Reubinoff B. (2014). PAX6 regulates melanogenesis in the retinal pigmented epithelium through feed-forward regulatory interactions with MITF. PLoS Genet..

[147] Singh R.K., Mallela R.K., Cornuet P.K., Reifler A.N., Chervenak A.P., West M.D., Wong K.Y., Nasonkin I.O. (2015). Characterization of Three-Dimensional Retinal Tissue Derived from Human Embryonic Stem Cells in Adherent Monolayer Cultures. Stem Cells Dev..

[148] O’Hara-Wright M., Gonzalez-Cordero A. (2020). Retinal organoids: A window into human retinal development. Development.

[149] Eiraku M., Takata N., Ishibashi H., Kawada M., Sakakura E., Okuda S., Sekiguchi K., Adachi T., Sasai Y. (2011). Self-organizing optic-cup morphogenesis in three-dimensional culture. Nature.

[150] Bharti K., Gasper M., Ou J., Brucato M., Clore-Gronenborn K., Pickel J., Arnheiter H. (2012). A regulatory loop involving PAX6, MITF, and WNT signaling controls retinal pigment epithelium development. PLoS Genet..

[151] Baumer N., Marquardt T., Stoykova A., Spieler D., Treichel D., Ashery-Padan R., Gruss P. (2003). Retinal pigmented epithelium determination requires the redundant activities of Pax2 and Pax6. Development.

[152] Capowski E.E., Samimi K., Mayerl S.J., Phillips M.J., Pinilla I., Howden S.E., Saha J., Jansen A.D., Edwards K.L., Jager L.D. (2019). Reproducibility and staging of 3D human retinal organoids across multiple pluripotent stem cell lines. Development.

[153] de Melo J., Miki K., Rattner A., Smallwood P., Zibetti C., Hirokawa K., Monuki E.S., Campochiaro P.A., Blackshaw S. (2012). Injury-independent induction of reactive gliosis in retina by loss of function of the LIM homeodomain TF Lhx2. Proc. Natl. Acad. Sci. USA.

[154] Surzenko N., Crowl T., Bachleda A., Langer L., Pevny L. (2013). SOX2 maintains the quiescent progenitor cell state of postnatal retinal Muller glia. Development.

[155] Muto A., Iida A., Satoh S., Watanabe S. (2009). The group E Sox genes Sox8 and Sox9 are regulated by Notch signaling and are required for Muller glial cell development in mouse retina. Exp. Eye Res..

[156] de Melo J., Clark B.S., Venkataraman A., Shiau F., Zibetti C., Blackshaw S. (2018). Ldb1- and Rnf12-dependent regulation of Lhx2 controls the relative balance between neurogenesis and gliogenesis in the retina. Development.

[157] Brightman D.S., Grant R.L., Ruzycki P.A., Suzuki R., Hennig A.K., Chen S. (2018). MLL1 is essential for retinal neurogenesis and horizontal inner neuron integrity. Sci. Rep..

[158] Aldiri I., Moore K.B., Hutcheson D.A., Zhang J., Vetter M.L. (2013). Polycomb repressive complex PRC2 regulates Xenopus retina development downstream of Wnt/beta-catenin signaling. Development.

[159] Fujimura N., Kuzelova A., Ebert A., Strnad H., Lachova J., Machon O., Busslinger M., Kozmik Z. (2018). Polycomb repression complex 2 is required for the maintenance of retinal progenitor cells and balanced retinal differentiation. Dev. Biol..

[160] Iida A., Iwagawa T., Baba Y., Satoh S., Mochizuki Y., Nakauchi H., Furukawa T., Koseki H., Murakami A., Watanabe S. (2015). Roles of histone H3K27 trimethylase Ezh2 in retinal proliferation and differentiation. Dev. Neurobiol..

[161] Iida A., Iwagawa T., Kuribayashi H., Satoh S., Mochizuki Y., Baba Y., Nakauchi H., Furukawa T., Koseki H., Murakami A. (2014). Histone demethylase Jmjd3 is required for the development of subsets of retinal bipolar cells. Proc. Natl. Acad. Sci. USA.

[162] Cheng L., Wong L.J., Yan N., Han R.C., Yu H., Guo C., Batsuuri K., Zinzuwadia A., Guan R., Cho K.S. (2018). Ezh2 does not mediate retinal ganglion cell homeostasis or their susceptibility to injury. PLoS ONE.

[163] Yan N., Cheng L., Cho K., Malik M.T., Xiao L., Guo C., Yu H., Zhu R., Rao R.C., Chen D.F. (2016). Postnatal onset of retinal degeneration by loss of embryonic Ezh2 repression of Six1. Sci. Rep..

[164] Mattar P., Stevanovic M., Nad I., Cayouette M. (2018). Casz1 controls higher-order nuclear organization in rod photoreceptors. Proc. Natl. Acad. Sci. USA.

[165] Cruz-Molina S., Respuela P., Tebartz C., Kolovos P., Nikolic M., Fueyo R., van Ijcken W.F.J., Grosveld F., Frommolt P., Bazzi H. (2017). PRC2 Facilitates the Regulatory Topology Required for Poised Enhancer Function during Pluripotent Stem Cell Differentiation. Cell Stem Cell.

[166] Barutcu A.R., Lajoie B.R., Fritz A.J., McCord R.P., Nickerson J.A., van Wijnen A.J., Lian J.B., Stein J.L., Dekker J., Stein G.S. (2016). SMARCA4 regulates gene expression and higher-order chromatin structure in proliferating mammary epithelial cells. Genome Res..

[167] Das A.V., James J., Bhattacharya S., Imbalzano A.N., Antony M.L., Hegde G., Zhao X., Mallya K., Ahmad F., Knudsen E. (2007). SWI/SNF chromatin remodeling ATPase Brm regulates the differentiation of early retinal stem cells/progenitors by influencing Brn3b expression and Notch signaling. J. Biol. Chem..

[168] Aldiri I., Ajioka I., Xu B., Zhang J., Chen X., Benavente C., Finkelstein D., Johnson D., Akiyama J., Pennacchio L.A. (2015). Brg1 coordinates multiple processes during retinogenesis and is a tumor suppressor in retinoblastoma. Development.

[169] Alver B.H., Kim K.H., Lu P., Wang X., Manchester H.E., Wang W., Haswell J.R., Park P.J., Roberts C.W. (2017). The SWI/SNF chromatin remodelling complex is required for maintenance of lineage specific enhancers. Nat. Commun..

[170] Perez-Cervantes C., Smith L.A., Nadadur R.D., Hughes A.E.O., Wang S., Corbo J.C., Cepko C., Lonfat N., Moskowitz I.P. (2020). Enhancer transcription identifies cis-regulatory elements for photoreceptor cell types. Development.

[171] Goodson N.B., Kaufman M.A., Park K.U., Brzezinski J.A. (2020). Simultaneous deletion of Prdm1 and Vsx2 enhancers in the retina alters photoreceptor and bipolar cell fate specification, yet differs from deleting both genes. Development.

[172] Emerson M.M., Cepko C.L. (2011). Identification of a retina-specific Otx2 enhancer element active in immature developing photoreceptors. Dev. Biol..

[173] Kaufman M.L., Goodson N.B., Park K.U., Schwanke M., Office E., Schneider S.R., Abraham J., Hensley A., Jones K.L., Brzezinski J.A. (2021). Initiation of Otx2 expression in the developing mouse retina requires a unique enhancer and either Ascl1 or Neurog2 activity. Development.

[174] Soufi A., Garcia M.F., Jaroszewicz A., Osman N., Pellegrini M., Zaret K.S. (2015). Pioneer TFs target partial DNA motifs on nucleosomes to initiate reprogramming. Cell.

[175] Schep A.N., Buenrostro J.D., Denny S.K., Schwartz K., Sherlock G., Greenleaf W.J. (2015). Structured nucleosome fingerprints enable high-resolution mapping of chromatin architecture within regulatory regions. Genome Res..

[176] Chen K., Xi Y., Pan X., Li Z., Kaestner K., Tyler J., Dent S., He X., Li W. (2013). DANPOS: Dynamic analysis of nucleosome position and occupancy by sequencing. Genome Res..

[177] Takahashi K., Yamanaka S. (2006). Induction of pluripotent stem cells from mouse embryonic and adult fibroblast cultures by defined factors. Cell.

[178] Liu X., Huang J., Chen T., Wang Y., Xin S., Li J., Pei G., Kang J. (2008). Yamanaka factors critically regulate the developmental signaling network in mouse embryonic stem cells. Cell Res..

[179] Sherwood R.I., Hashimoto T., O’Donnell C.W., Lewis S., Barkal A.A., van Hoff J.P., Karun V., Jaakkola T., Gifford D.K. (2014). Discovery of directional and nondirectional pioneer TFs by modeling DNase profile magnitude and shape. Nat. Biotechnol..

[180] Grant C.E., Bailey T.L., Noble W.S. (2011). FIMO: Scanning for occurrences of a given motif. Bioinformatics.

[181] Pique-Regi R., Degner J.F., Pai A.A., Gaffney D.J., Gilad Y., Pritchard J.K. (2011). Accurate inference of TF binding from DNA sequence and chromatin accessibility data. Genome Res..

[182] Liu S., Zibetti C., Wan J., Wang G., Blackshaw S., Qian J. (2017). Assessing the model transferability for prediction of TF binding sites based on chromatin accessibility. BMC Bioinform..

[183] McLaren W., Gil L., Hunt S.E., Riat H.S., Ritchie G.R., Thormann A., Flicek P., Cunningham F. (2016). The Ensembl Variant Effect Predictor. Genome Biol..

[184] Iejima D., Itabashi T., Kawamura Y., Noda T., Yuasa S., Fukuda K., Oka C., Iwata T. (2015). HTRA1 (high temperature requirement A serine peptidase 1) gene is transcriptionally regulated by insertion/deletion nucleotides located at the 3′ end of the ARMS2 (age-related maculopathy susceptibility 2) gene in patients with age-related macular degeneration. J. Biol. Chem..

[185] Stein-O’Brien G.L., Clark B.S., Sherman T., Zibetti C., Hu Q., Sealfon R., Liu S., Qian J., Colantuoni C., Blackshaw S. (2019). Decomposing Cell Identity for Transfer Learning across Cellular Measurements, Platforms, Tissues, and Species. Cell Syst..

[186] Pevny L.H., Lovell-Badge R. (1997). Sox genes find their feet. Curr. Opin. Genet. Dev..

[187] Favaro R., Valotta M., Ferri A.L., Latorre E., Mariani J., Giachino C., Lancini C., Tosetti V., Ottolenghi S., Taylor V. (2009). Hippocampal development and neural stem cell maintenance require Sox2-dependent regulation of Shh. Nat. Neurosci..

[188] Ferri A.L., Cavallaro M., Braida D., Di Cristofano A., Canta A., Vezzani A., Ottolenghi S., Pandolfi P.P., Sala M., DeBiasi S. (2004). Sox2 deficiency causes neurodegeneration and impaired neurogenesis in the adult mouse brain. Development.

[189] Wegner M., Stolt C.C. (2005). From stem cells to neurons and glia: A Soxist’s view of neural development. Trends Neurosci..

[190] Wegner M. (2011). SOX after SOX: SOXession regulates neurogenesis. Genes Dev..

[191] Taranova O.V., Magness S.T., Fagan B.M., Wu Y., Surzenko N., Hutton S.R., Pevny L.H. (2006). SOX2 is a dose-dependent regulator of retinal neural progenitor competence. Genes Dev..

[192] Lodato M.A., Ng C.W., Wamstad J.A., Cheng A.W., Thai K.K., Fraenkel E., Jaenisch R., Boyer L.A. (2013). SOX2 co-occupies distal enhancer elements with distinct POU factors in ESCs and NPCs to specify cell state. PLoS Genet..

[193] Lin Y.P., Ouchi Y., Satoh S., Watanabe S. (2009). Sox2 plays a role in the induction of amacrine and Muller glial cells in mouse retinal progenitor cells. Investig. Opthalmol. Vis. Sci..

[194] Wohl S.G., Hooper M.J., Reh T.A. (2019). MicroRNAs miR-25, let-7 and miR-124 regulate the neurogenic potential of Muller glia in mice. Development.

[195] Poche R.A., Furuta Y., Chaboissier M.C., Schedl A., Behringer R.R. (2008). Sox9 is expressed in mouse multipotent retinal progenitor cells and functions in Muller glial cell development. J. Comp. Neurol..

[196] Masuda T., Wahlin K., Wan J., Hu J., Maruotti J., Yang X., Iacovelli J., Wolkow N., Kist R., Dunaief J.L. (2014). TF SOX9 plays a key role in the regulation of visual cycle gene expression in the retinal pigment epithelium. J. Biol. Chem..

[197] Cohen-Tayar Y., Cohen H., Mitiagin Y., Abravanel Z., Levy C., Idelson M., Reubinoff B., Itzkovitz S., Raviv S., Kaestner K.H. (2018). Pax6 regulation of Sox9 in the mouse retinal pigmented epithelium controls its timely differentiation and choroid vasculature development. Development.

[198] Menzel-Severing J., Zenkel M., Polisetti N., Sock E., Wegner M., Kruse F.E., Schlotzer-Schrehardt U. (2018). TF profiling identifies Sox9 as regulator of proliferation and differentiation in corneal epithelial stem/progenitor cells. Sci. Rep..

[199] Vong K.I., Leung C.K., Behringer R.R., Kwan K.M. (2015). Sox9 is critical for suppression of neurogenesis but not initiation of gliogenesis in the cerebellum. Mol. Brain.

[200] Kang P., Lee H.K., Glasgow S.M., Finley M., Donti T., Gaber Z.B., Graham B.H., Foster A.E., Novitch B.G., Gronostajski R.M. (2012). Sox9 and NFIA coordinate a transcriptional regulatory cascade during the initiation of gliogenesis. Neuron.

[201] Tomita K., Nakanishi S., Guillemot F., Kageyama R. (1996). Mash1 promotes neuronal differentiation in the retina. Genes Cells.

[202] Ramachandran R., Fausett B.V., Goldman D. (2010). Ascl1a regulates Muller glia dedifferentiation and retinal regeneration through a Lin-28-dependent, let-7 microRNA signalling pathway. Nat. Cell Biol..

[203] Jorstad N.L., Wilken M.S., Grimes W.N., Wohl S.G., VandenBosch L.S., Yoshimatsu T., Wong R.O., Rieke F., Reh T.A. (2017). Stimulation of functional neuronal regeneration from Muller glia in adult mice. Nature.

[204] Hjelm B.E., Rosenberg J.B., Szelinger S., Sue L.I., Beach T.G., Huentelman M.J., Craig D.W. (2011). Induction of pluripotent stem cells from autopsy donor-derived somatic cells. Neurosci. Lett..

[205] Wernig M., Lengner C.J., Hanna J., Lodato M.A., Steine E., Foreman R., Staerk J., Markoulaki S., Jaenisch R. (2008). A drug-inducible transgenic system for direct reprogramming of multiple somatic cell types. Nat. Biotechnol..

[206] Gao X., Wang X., Xiong W., Chen J. (2016). In vivo reprogramming reactive glia into iPSCs to produce new neurons in the cortex following traumatic brain injury. Sci. Rep..

[207] Todd L., Fischer A.J. (2015). Hedgehog signaling stimulates the formation of proliferating Muller glia-derived progenitor cells in the chick retina. Development.

[208] Todd L., Suarez L., Quinn C., Fischer A.J. (2018). Retinoic Acid-Signaling Regulates the Proliferative and Neurogenic Capacity of Muller Glia-Derived Progenitor Cells in the Avian Retina. Stem Cells.

[209] Zelinka C.P., Volkov L., Goodman Z.A., Todd L., Palazzo I., Bishop W.A., Fischer A.J. (2016). mTor signaling is required for the formation of proliferating Muller glia-derived progenitor cells in the chick retina. Development.

[210] Moore D.L., Blackmore M.G., Hu Y., Kaestner K.H., Bixby J.L., Lemmon V.P., Goldberg J.L. (2009). KLF family members regulate intrinsic axon regeneration ability. Science.

[211] Fang J., Shaw P.X., Wang Y., Goldberg J.L. (2016). Kruppel-Like Factor 4 (KLF4) Is Not Required for Retinal Cell Differentiation. eNeuro.

[212] Marcos-Mondejar P., Peregrin S., Li J.Y., Carlsson L., Tole S., Lopez-Bendito G. (2012). The Lhx2 TF controls thalamocortical axonal guidance by specific regulation of robo1 and robo2 receptors. J. Neurosci..

[213] O’Sullivan M.L., Punal V.M., Kerstein P.C., Brzezinski J.A., Glaser T., Wright K.M., Kay J.N. (2017). Astrocytes follow ganglion cell axons to establish an angiogenic template during retinal development. Glia.

[214] Rocha-Martins M., de Toledo B.C., Santos-Franca P.L., Oliveira-Valenca V.M., Vieira-Vieira C.H., Matos-Rodrigues G.E., Linden R., Norden C., Martins R.A.P., Silveira M.S. (2019). De novo genesis of retinal ganglion cells by targeted expression of Klf4 in vivo. Development.

[215] Brown N.L., Patel S., Brzezinski J., Glaser T. (2001). Math5 is required for retinal ganglion cell and optic nerve formation. Development.

[216] Pan L., Deng M., Xie X., Gan L. (2008). ISL1 and BRN3B co-regulate the differentiation of murine retinal ganglion cells. Development.

[217] Brzezinski J.A., Prasov L., Glaser T. (2012). Math5 defines the ganglion cell competence state in a subpopulation of retinal progenitor cells exiting the cell cycle. Dev. Biol..

[218] Wu F., Kaczynski T.J., Sethuramanujam S., Li R., Jain V., Slaughter M., Mu X. (2015). Two TFs, Pou4f2 and Isl1, are sufficient to specify the retinal ganglion cell fate. Proc. Natl. Acad. Sci. USA.

[219] Miesfeld J.B., Ghiasvand N.M., Marsh-Armstrong B., Marsh-Armstrong N., Miller E.B., Zhang P., Manna S.K., Zawadzki R.J., Brown N.L., Glaser T. (2020). The Atoh7 remote enhancer provides transcriptional robustness during retinal ganglion cell development. Proc. Natl. Acad. Sci. USA.

[220] Dupacova N., Antosova B., Paces J., Kozmik Z. (2021). Meis homeobox genes control progenitor competence in the retina. Proc. Natl. Acad. Sci. USA.

[221] Hoang T., Wang J., Boyd P., Wang F., Santiago C., Jiang L., Yoo S., Lahne M., Todd L.J., Jia M. (2020). Gene regulatory networks controlling vertebrate retinal regeneration. Science.

[222] Deneen B., Ho R., Lukaszewicz A., Hochstim C.J., Gronostajski R.M., Anderson D.J. (2006). The TF NFIA controls the onset of gliogenesis in the developing spinal cord. Neuron.

[223] Matuzelski E., Bunt J., Harkins D., Lim J.W.C., Gronostajski R.M., Richards L.J., Harris L., Piper M. (2017). Transcriptional regulation of Nfix by NFIB drives astrocytic maturation within the developing spinal cord. Dev. Biol..

[224] Nagao M., Ogata T., Sawada Y., Gotoh Y. (2016). Zbtb20 promotes astrocytogenesis during neocortical development. Nat. Commun..

[225] Jorstad N.L., Wilken M.S., Todd L., Finkbeiner C., Nakamura P., Radulovich N., Hooper M.J., Chitsazan A., Wilkerson B.A., Rieke F. (2020). STAT Signaling Modifies Ascl1 Chromatin Binding and Limits Neural Regeneration from Muller Glia in Adult Mouse Retina. Cell Rep..

[226] Wang J., Zibetti C., Shang P., Sripathi S.R., Zhang P., Cano M., Hoang T., Xia S., Ji H., Merbs S.L. (2018). ATAC-Seq analysis reveals a widespread decrease of chromatin accessibility in age-related macular degeneration. Nat. Commun..

[227] Bourne R.R.A., Flaxman S.R., Braithwaite T., Cicinelli M.V., Das A., Jonas J.B., Keeffe J., Kempen J.H., Leasher J., Limburg H. (2017). Magnitude, temporal trends, and projections of the global prevalence of blindness and distance and near vision impairment: A systematic review and meta-analysis. Lancet Glob. Health.

[228] Jonas J.B., Cheung C.M.G., Panda-Jonas S. (2017). Updates on the Epidemiology of Age-Related Macular Degeneration. Asia Pac. J. Ophthalmol..

[229] Cachafeiro M., Bemelmans A.P., Samardzija M., Afanasieva T., Pournaras J.A., Grimm C., Kostic C., Philippe S., Wenzel A., Arsenijevic Y. (2013). Hyperactivation of retina by light in mice leads to photoreceptor cell death mediated by VEGF and retinal pigment epithelium permeability. Cell Death Dis..

[230] Raoul W., Keller N., Rodero M., Behar-Cohen F., Sennlaub F., Combadiere C. (2008). Role of the chemokine receptor CX3CR1 in the mobilization of phagocytic retinal microglial cells. J. Neuroimmunol..

[231] Ma W., Zhang Y., Gao C., Fariss R.N., Tam J., Wong W.T. (2017). Monocyte infiltration and proliferation reestablish myeloid cell homeostasis in the mouse retina following retinal pigment epithelial cell injury. Sci. Rep..

[232] Thakkinstian A., McEvoy M., Chakravarthy U., Chakrabarti S., McKay G.J., Ryu E., Silvestri G., Kaur I., Francis P., Iwata T. (2012). The association between complement component 2/complement factor B polymorphisms and age-related macular degeneration: A HuGE review and meta-analysis. Am. J. Epidemiol..

[233] Szatmari-Toth M., Kristof E., Vereb Z., Akhtar S., Facsko A., Fesus L., Kauppinen A., Kaarniranta K., Petrovski G. (2016). Clearance of autophagy-associated dying retinal pigment epithelial cells—A possible source for inflammation in age-related macular degeneration. Cell Death Dis..

[234] Porter L.F., Saptarshi N., Fang Y., Rathi S., den Hollander A.I., de Jong E.K., Clark S.J., Bishop P.N., Olsen T.W., Liloglou T. (2019). Whole-genome methylation profiling of the retinal pigment epithelium of individuals with age-related macular degeneration reveals differential methylation of the SKI, GTF2H4, and TNXB genes. Clin. Epigenet..

[235] Menon M., Mohammadi S., Davila-Velderrain J., Goods B.A., Cadwell T.D., Xing Y., Stemmer-Rachamimov A., Shalek A.K., Love J.C., Kellis M. (2019). Single-cell transcriptomic atlas of the human retina identifies cell types associated with age-related macular degeneration. Nat. Commun..

[236] Sahaboglu A., Paquet-Durand O., Dietter J., Dengler K., Bernhard-Kurz S., Ekstrom P.A., Hitzmann B., Ueffing M., Paquet-Durand F. (2013). Retinitis pigmentosa: Rapid neurodegeneration is governed by slow cell death mechanisms. Cell Death Dis..

[237] Potic J., Mbefo M., Berger A., Nicolas M., Wanner D., Kostic C., Matet A., Behar-Cohen F., Moulin A., Arsenijevic Y. (2020). An In Vitro Model of Human Retinal Detachment Reveals Successive Death Pathway Activations. Front. Neurosci..

[238] Aapola U., Liiv I., Peterson P. (2002). Imprinting regulator DNMT3L is a transcriptional repressor associated with histone deacetylase activity. Nucleic Acids Res..

[239] Rountree M.R., Bachman K.E., Baylin S.B. (2000). DNMT1 binds HDAC2 and a new co-repressor, DMAP1, to form a complex at replication foci. Nat. Genet..

[240] Wahlin K.J., Enke R.A., Fuller J.A., Kalesnykas G., Zack D.J., Merbs S.L. (2013). Epigenetics and cell death: DNA hypermethylation in programmed retinal cell death. PLoS ONE.

[241] Farinelli P., Perera A., Arango-Gonzalez B., Trifunovic D., Wagner M., Carell T., Biel M., Zrenner E., Michalakis S., Paquet-Durand F. (2014). DNA methylation and differential gene regulation in photoreceptor cell death. Cell Death Dis..

[242] Corso-Diaz X., Jaeger C., Chaitankar V., Swaroop A. (2018). Epigenetic control of gene regulation during development and disease: A view from the retina. Prog. Retin. Eye Res..

[243] Gemenetzi M., Lotery A.J. (2014). The role of epigenetics in age-related macular degeneration. Eye.

[244] Pennington K.L., DeAngelis M.M. (2016). Epidemiology of age-related macular degeneration (AMD): Associations with cardiovascular disease phenotypes and lipid factors. Eye Vis..

[245] Wei L., Liu B., Tuo J., Shen D., Chen P., Li Z., Liu X., Ni J., Dagur P., Sen H.N. (2012). Hypomethylation of the IL17RC promoter associates with age-related macular degeneration. Cell Rep..

[246] Biswas S., Thomas A.A., Chen S., Aref-Eshghi E., Feng B., Gonder J., Sadikovic B., Chakrabarti S. (2018). MALAT1: An Epigenetic Regulator of Inflammation in Diabetic Retinopathy. Sci. Rep..

[247] Sancho-Pelluz J., Alavi M.V., Sahaboglu A., Kustermann S., Farinelli P., Azadi S., van Veen T., Romero F.J., Paquet-Durand F., Ekstrom P. (2010). Excessive HDAC activation is critical for neurodegeneration in the rd1 mouse. Cell Death Dis..

[248] Zheng S., Xiao L., Liu Y., Wang Y., Cheng L., Zhang J., Yan N., Chen D. (2018). DZNep inhibits H3K27me3 deposition and delays retinal degeneration in the rd1 mice. Cell Death Dis..

[249] Trifunovic D., Sahaboglu A., Kaur J., Mencl S., Zrenner E., Ueffing M., Arango-Gonzalez B., Paquet-Durand F. (2012). Neuroprotective strategies for the treatment of inherited photoreceptor degeneration. Curr. Mol. Med..

[250] Mitton K.P., Guzman A.E., Deshpande M., Byrd D., DeLooff C., Mkoyan K., Zlojutro P., Wallace A., Metcalf B., Laux K. (2014). Different effects of valproic acid on photoreceptor loss in Rd1 and Rd10 retinal degeneration mice. Mol. Vis..

[251] Trifunovic D., Arango-Gonzalez B., Comitato A., Barth M., Del Amo E.M., Kulkarni M., Sahaboglu A., Hauck S.M., Urtti A., Arsenijevic Y. (2016). HDAC inhibition in the cpfl1 mouse protects de-generating cone photoreceptors in vivo. Hum. Mol. Genet..

[252] Klingeborn M., Dismuke W.M., Bowes Rickman C., Stamer W.D. (2017). Roles of exosomes in the normal and diseased eye. Prog. Retin. Eye Res..

[253] Wang J.H., Ling D., Tu L., van Wijngaarden P., Dusting G.J., Liu G.S. (2017). Gene therapy for diabetic retinopathy: Are we ready to make the leap from bench to bedside?. Pharmacol. Ther..

[254] Gonzalez-Fernandez F.M., Bianchera A., Gasco P., Nicoli S., Pescina S. (2021). Lipid-Based Nanocarriers for Ophthalmic Administration: Towards Experimental Design Implementation. Pharmaceutics.

[255] Amadio M., Pascale A., Cupri S., Pignatello R., Osera C., D’Agata V., D’Amico A.G., Leggio G.M., Ruozi B., Govoni S. (2016). Nanosystems based on siRNA silencing HuR expression counteract diabetic retinopathy in rat. Pharmacol. Res..

[256] Campochiaro P.A. (2006). Potential applications for RNAi to probe pathogenesis and develop new treatments for ocular disorders. Gene Ther..

[257] Haurigot V., Villacampa P., Ribera A., Bosch A., Ramos D., Ruberte J., Bosch F. (2012). Long-term retinal PEDF overexpression prevents neovascularization in a murine adult model of retinopathy. PLoS ONE.

[258] Achberger K., Cipriano M., Duchs M.J., Schon C., Michelfelder S., Stierstorfer B., Lamla T., Kauschke S.G., Chuchuy J., Roosz J. (2021). Human stem cell-based retina on chip as new translational model for validation of AAV retinal gene therapy vectors. Stem Cell Rep..

[259] Wiley L.A., Burnight E.R., Kaalberg E.E., Jiao C., Riker M.J., Halder J.A., Luse M.A., Han I.C., Russell S.R., Sohn E.H. (2018). Assessment of Adeno-Associated Virus Serotype Tropism in Human Retinal Explants. Hum. Gene Ther..

[260] Chung S.H., Sin T.N., Ngo T., Yiu G. (2020). CRISPR Technology for Ocular Angiogenesis. Front. Genome Ed..

[261] Fenner B.J., Tan T.E., Barathi A.V., Tun S.B.B., Yeo S.W., Tsai A.S.H., Lee S.Y., Cheung C.M.G., Chan C.M., Mehta J.S. (2021). Gene-Based Therapeutics for Inherited Retinal Diseases. Front. Genet..

[262] Russell S., Bennett J., Wellman J.A., Chung D.C., Yu Z.F., Tillman A., Wittes J., Pappas J., Elci O., McCague S. (2017). Efficacy and safety of voretigene neparvovec (AAV2-hRPE65v2) in patients with RPE65-mediated inherited retinal dystrophy: A randomised, controlled, open-label, phase 3 trial. Lancet.

[263] Latella M.C., Di Salvo M.T., Cocchiarella F., Benati D., Grisendi G., Comitato A., Marigo V., Recchia A. (2016). In Vivo Editing of the Human Mutant Rhodopsin Gene by Electroporation of Plasmid-based CRISPR/Cas9 in the Mouse Retina. Mol. Ther. Nucleic Acids.

[264] Jain A., Zode G., Kasetti R.B., Ran F.A., Yan W., Sharma T.P., Bugge K., Searby C.C., Fingert J.H., Zhang F. (2017). CRISPR-Cas9-based treatment of myocilin-associated glaucoma. Proc. Natl. Acad. Sci. USA.

[265] Cideciyan A.V., Sudharsan R., Dufour V.L., Massengill M.T., Iwabe S., Swider M., Lisi B., Sumaroka A., Marinho L.F., Appelbaum T. (2018). Mutation-independent rhodopsin gene therapy by knock-down and replacement with a single AAV vector. Proc. Natl. Acad. Sci. USA.

[266] Woo M. (2019). Eyes hint at hidden mental-health conditions. Nature.

[267] Wu W.H., Tsai Y.T., Huang I.W., Cheng C.H., Hsu C.W., Cui X., Ryu J., Quinn P.M.J., Caruso S.M., Lin C.S. (2022). CRISPR genome surgery in a novel humanized model for autosomal dominant retinitis pigmentosa. Mol. Ther..

[268] Moreno A.M., Fu X., Zhu J., Katrekar D., Shih Y.V., Marlett J., Cabotaje J., Tat J., Naughton J., Lisowski L. (2018). In Situ Gene Therapy via AAV-CRISPR-Cas9-Mediated Targeted Gene Regulation. Mol. Ther..

[269] Zetsche B., Volz S.E., Zhang F. (2015). A split-Cas9 architecture for inducible genome editing and transcription modulation. Nat. Biotechnol..

[270] Keser V., Khan A., Siddiqui S., Lopez I., Ren H., Qamar R., Nadaf J., Majewski J., Chen R., Koenekoop R.K. (2017). The Genetic Causes of Nonsyndromic Congenital Retinal Detachment: A Genetic and Phenotypic Study of Pakistani Families. Investig. Opthalmol. Vis. Sci..

[271] Ghiasvand N.M., Rudolph D.D., Mashayekhi M., Brzezinski J.A., Goldman D., Glaser T. (2011). Deletion of a remote enhancer near ATOH7 disrupts retinal neurogenesis, causing NCRNA disease. Nat. Neurosci..

[272] Bhatia S., Bengani H., Fish M., Brown A., Divizia M.T., de Marco R., Damante G., Grainger R., van Heyningen V., Kleinjan D.A. (2013). Disruption of autoregulatory feedback by a mutation in a remote, ultraconserved PAX6 enhancer causes aniridia. Am. J. Hum. Genet..

[273] Bremond-Gignac D., Bitoun P., Reis L.M., Copin H., Murray J.C., Semina E.V. (2010). Identification of dominant FOXE3 and PAX6 mutations in patients with congenital cataract and aniridia. Mol. Vis..

[274] Van Schil K., Karlstetter M., Aslanidis A., Dannhausen K., Azam M., Qamar R., Leroy B.P., Depasse F., Langmann T., De Baere E. (2016). Autosomal recessive retinitis pigmentosa with homozygous rhodopsin mutation E150K and non-coding cis-regulatory variants in CRX-binding regions of SAMD7. Sci. Rep..

[275] de Bruijn S.E., Fiorentino A., Ottaviani D., Fanucchi S., Melo U.S., Corral-Serrano J.C., Mulders T., Georgiou M., Rivolta C., Pontikos N. (2020). Structural Variants Create New Topological-Associated Domains and Ectopic Retinal Enhancer-Gene Contact in Dominant Retinitis Pigmentosa. Am. J. Hum. Genet..

[276] Zalatan J.G., Lee M.E., Almeida R., Gilbert L.A., Whitehead E.H., La Russa M., Tsai J.C., Weissman J.S., Dueber J.E., Qi L.S. (2015). Engineering complex synthetic transcriptional programs with CRISPR RNA scaffolds. Cell.

[277] Nunez J.K., Chen J., Pommier G.C., Cogan J.Z., Replogle J.M., Adriaens C., Ramadoss G.N., Shi Q., Hung K.L., Samelson A.J. (2021). Genome-wide programmable transcriptional memory by CRISPR-based epigenome editing. Cell.

[278] Zhuo C., Zhang J., Lee J.H., Jiao J., Cheng D., Liu L., Kim H.W., Tao Y., Li M. (2021). Spatiotemporal control of CRISPR/Cas9 gene editing. Signal Transduct. Target. Ther..

[279] Mandegar M.A., Huebsch N., Frolov E.B., Shin E., Truong A., Olvera M.P., Chan A.H., Miyaoka Y., Holmes K., Spencer C.I. (2016). CRISPR Interference Efficiently Induces Specific and Reversible Gene Silencing in Human iPSCs. Cell Stem Cell.

[280] Konermann S., Brigham M.D., Trevino A.E., Joung J., Abudayyeh O.O., Barcena C., Hsu P.D., Habib N., Gootenberg J.S., Nishimasu H. (2015). Genome-scale transcriptional activation by an engineered CRISPR-Cas9 complex. Nature.

[281] Bin Moon S., Lee J.M., Kang J.G., Lee N.E., Ha D.I., Kim D.Y., Kim S.H., Yoo K., Kim D., Ko J.H. (2018). Highly efficient genome editing by CRISPR-Cpf1 using CRISPR RNA with a uridinylate-rich 3′-overhang. Nat. Commun..

[282] Sahel J.A., Boulanger-Scemama E., Pagot C., Arleo A., Galluppi F., Martel J.N., Esposti S.D., Delaux A., de Saint Aubert J.B., de Montleau C. (2021). Partial recovery of visual function in a blind patient after optogenetic therapy. Nat. Med..

[283] Fernandez E. (2018). Development of visual Neuroprostheses: Trends and challenges. Bioelectron. Med..

[284] Kashani A.H., Lebkowski J.S., Rahhal F.M., Avery R.L., Salehi-Had H., Dang W., Lin C.M., Mitra D., Zhu D., Thomas B.B. (2018). A bioengineered retinal pigment epithelial monolayer for advanced, dry age-related macular degeneration. Sci. Transl. Med..

[285] Ikelle L., Al-Ubaidi M.R., Naash M.I. (2020). Pluripotent Stem Cells for the Treatment of Retinal Degeneration: Current Strategies and Future Directions. Front. Cell Dev. Biol..

[286] Miyake A., Araki M. (2014). Retinal stem/progenitor cells in the ciliary marginal zone complete retinal regeneration: A study of retinal regeneration in a novel animal model. Dev. Neurobiol..

[287] Marcucci F., Murcia-Belmonte V., Wang Q., Coca Y., Ferreiro-Galve S., Kuwajima T., Khalid S., Ross M.E., Mason C., Herrera E. (2016). The Ciliary Margin Zone of the Mammalian Retina Generates Retinal Ganglion Cells. Cell Rep..

[288] Reh T.A., Nagy T. (1987). A possible role for the vascular membrane in retinal regeneration in Rana catesbienna tadpoles. Dev. Biol..

[289] Mitashov V.I. (1996). Mechanisms of retina regeneration in urodeles. Int. J. Dev. Biol..

[290] Del Rio-Tsonis K., Tsonis P.A. (2003). Eye regeneration at the molecular age. Dev. Dyn..

[291] Klein L.R., MacLeish P.R., Wiesel T.N. (1990). Immunolabelling by a newt retinal pigment epithelium antibody during retinal development and regeneration. J. Comp. Neurol..

[292] Casco-Robles M.M., Nakamura K., Casco-Robles M.M., Kunahong A., Inami W., Toyama F., Maruo F., Chiba C. (2016). Turning the fate of reprogramming cells from retinal disorder to regeneration by Pax6 in newts. Sci. Rep..

[293] Islam M.R., Nakamura K., Casco-Robles M.M., Kunahong A., Inami W., Toyama F., Maruo F., Chiba C. (2014). The newt reprograms mature RPE cells into a unique multipotent state for retinal regeneration. Sci. Rep..

[294] Maier W., Wolburg H. (1979). Regeneration of the goldfish retina after exposure to different doses of ouabain. Cell Tissue Res..

[295] Knight J.K., Raymond P.A. (1995). Retinal pigmented epithelium does not transdifferentiate in adult goldfish. J. Neurobiol..

[296] Coulombre J.L., Coulombre A.J. (1965). Regeneration of neural retina from the pigmented epithelium in the chick embryo. Dev. Biol..

[297] Zhao S., Thornquist S.C., Barnstable C.J. (1995). In vitro transdifferentiation of embryonic rat retinal pigment epithelium to neural retina. Brain Res..

[298] Moshiri A., Reh T.A. (2004). Persistent progenitors at the retinal margin of ptc+/− mice. J. Neurosci..

[299] Fischer A.J., Reh T.A. (2001). Muller glia are a potential source of neural regeneration in the postnatal chicken retina. Nat. Neurosci..

[300] Fausett B.V., Goldman D. (2006). A role for alpha1 tubulin-expressing Muller glia in regeneration of the injured zebrafish retina. J. Neurosci..

[301] Lahne M., Brecker M., Jones S.E., Hyde D.R. (2020). The Regenerating Adult Zebrafish Retina Recapitulates Developmental Fate Specification Programs. Front. Cell Dev. Biol..

[302] Fuhrmann S., Zou C., Levine E.M. (2014). Retinal pigment epithelium development, plasticity, and tissue homeostasis. Exp. Eye Res..

[303] Dvoriantchikova G., Seemungal R.J., Ivanov D. (2019). The epigenetic basis for the impaired ability of adult murine retinal pigment epithelium cells to regenerate retinal tissue. Sci. Rep..

[304] Levine E.M., Close J., Fero M., Ostrovsky A., Reh T.A. (2000). p27(Kip1) regulates cell cycle withdrawal of late multipotent progenitor cells in the mammalian retina. Dev. Biol..

[305] Joly S., Pernet V., Samardzija M., Grimm C. (2011). Pax6-positive Muller glia cells express cell cycle markers but do not proliferate after photoreceptor injury in the mouse retina. Glia.

[306] Beveridge N.J., Tooney P.A., Carroll A.P., Tran N., Cairns M.J. (2009). Down-regulation of miR-17 family expression in response to retinoic acid induced neuronal differentiation. Cell. Signal..

[307] Foshay K.M., Gallicano G.I. (2009). miR-17 family miRNAs are expressed during early mammalian development and regulate stem cell differentiation. Dev. Biol..

[308] Naka-Kaneda H., Nakamura S., Igarashi M., Aoi H., Kanki H., Tsuyama J., Tsutsumi S., Aburatani H., Shimazaki T., Okano H. (2014). The miR-17/106-p38 axis is a key regulator of the neurogenic-to-gliogenic transition in developing neural stem/progenitor cells. Proc. Natl. Acad. Sci. USA.

[309] Trompeter H.I., Abbad H., Iwaniuk K.M., Hafner M., Renwick N., Tuschl T., Schira J., Muller H.W., Wernet P. (2011). MicroRNAs MiR-17, MiR-20a, and MiR-106b act in concert to modulate E2F activity on cell cycle arrest during neuronal lineage differentiation of USSC. PLoS ONE.

[310] Yang Y., Sun B., Huang J., Xu L., Pan J., Fang C., Li M., Li G., Tao Y., Yang X. (2017). Up-regulation of miR-325-3p suppresses pineal aralkylamine *N*-acetyltransferase (Aanat) after neonatal hypoxia-ischemia brain injury in rats. Brain Res..

[311] Roesch K., Jadhav A.P., Trimarchi J.M., Stadler M.B., Roska B., Sun B.B., Cepko C.L. (2008). The transcriptome of retinal Muller glial cells. J. Comp. Neurol..

[312] Karl M.O., Hayes S., Nelson B.R., Tan K., Buckingham B., Reh T.A. (2008). Stimulation of neural regeneration in the mouse retina. Proc. Natl. Acad. Sci. USA.

[313] Ueki Y., Wilken M.S., Cox K.E., Chipman L., Jorstad N., Sternhagen K., Simic M., Ullom K., Nakafuku M., Reh T.A. (2015). Transgenic expression of the proneural TF Ascl1 in Muller glia stimulates retinal regeneration in young mice. Proc. Natl. Acad. Sci. USA.

[314] Wilken M.S., Reh T.A. (2016). Retinal regeneration in birds and mice. Curr. Opin. Genet. Dev..

[315] Turkalj B., Quallich D., Bessert D.A., Kramer A.C., Cook T.A., Thummel R. (2021). Development and characterization of a chronic photoreceptor degeneration model in adult zebrafish that does not trigger a regenerative response. Exp. Eye Res..

[316] Singh H.P., Wang S., Stachelek K., Lee S., Reid M.W., Thornton M.E., Craft C.M., Grubbs B.H., Cobrinik D. (2018). Developmental stage-specific proliferation and retinoblastoma genesis in RB-deficient human but not mouse cone precursors. Proc. Natl. Acad. Sci. USA.

[317] Aydin B., Kakumanu A., Rossillo M., Moreno-Estelles M., Garipler G., Ringstad N., Flames N., Mahony S., Mazzoni E.O. (2019). Proneural factors Ascl1 and Neurog2 contribute to neuronal subtype identities by establishing distinct chromatin landscapes. Nat. Neurosci..

[318] Yao K., Qiu S., Tian L., Snider W.D., Flannery J.G., Schaffer D.V., Chen B. (2016). Wnt Regulates Proliferation and Neurogenic Potential of Muller Glial Cells via a Lin28/let-7 miRNA-Dependent Pathway in Adult Mammalian Retinas. Cell Rep..

[319] Rueda E.M., Hall B.M., Hill M.C., Swinton P.G., Tong X., Martin J.F., Poche R.A. (2019). The Hippo Pathway Blocks Mammalian Retinal Muller Glial Cell Reprogramming. Cell Rep..

[320] Todd L., Hooper M.J., Haugan A.K., Finkbeiner C., Jorstad N., Radulovich N., Wong C.K., Donaldson P.C., Jenkins W., Chen Q. (2021). Efficient stimulation of retinal regeneration from Muller glia in adult mice using combinations of proneural bHLH TFs. Cell Rep..

[321] Lu Y., Brommer B., Tian X., Krishnan A., Meer M., Wang C., Vera D.L., Zeng Q., Yu D., Bonkowski M.S. (2020). Reprogramming to recover youthful epigenetic information and restore vision. Nature.

[322] Heinz S., Benner C., Spann N., Bertolino E., Lin Y.C., Laslo P., Cheng J.X., Murre C., Singh H., Glass C.K. (2010). Simple combinations of lineage-determining TFs prime cis-regulatory elements required for macrophage and B cell identities. Mol. Cell.

[323] Berger M.F., Bulyk M.L. (2006). Protein binding microarrays (PBMs) for rapid, high-throughput characterization of the sequence specificities of DNA binding proteins. Methods Mol. Biol..

[324] Tuerk C., Gold L. (1990). Systematic evolution of ligands by exponential enrichment: RNA ligands to bacteriophage T4 DNA polymerase. Science.

[325] O’Malley R.C., Huang S.C., Song L., Lewsey M.G., Bartlett A., Nery J.R., Galli M., Gallavotti A., Ecker J.R. (2016). Cistrome and Epicistrome Features Shape the Regulatory DNA Landscape. Cell.

[326] Bartlett A., O’Malley R.C., Huang S.C., Galli M., Nery J.R., Gallavotti A., Ecker J.R. (2017). Mapping genome-wide transcription-factor binding sites using DAP-seq. Nat. Protoc..

[327] Baumgart L.A., Lee J.E., Salamov A., Dilworth D.J., Na H., Mingay M., Blow M.J., Zhang Y., Yoshinaga Y., Daum C.G. (2021). Persistence and plasticity in bacterial gene regulation. Nat. Methods.

[328] Orenstein Y., Shamir R. (2014). A comparative analysis of TF binding models learned from PBM, HT-SELEX and ChIP data. Nucleic Acids Res..

[329] van Helden J. (2003). Regulatory sequence analysis tools. Nucleic Acids Res..

[330] Matys V., Kel-Margoulis O.V., Fricke E., Liebich I., Land S., Barre-Dirrie A., Reuter I., Chekmenev D., Krull M., Hornischer K. (2006). TRANSFAC and its module TRANSCompel: Transcriptional gene regulation in eukaryotes. Nucleic Acids Res..

[331] Mathelier A., Fornes O., Arenillas D.J., Chen C.Y., Denay G., Lee J., Shi W., Shyr C., Tan G., Worsley-Hunt R. (2016). JASPAR 2016: A major expansion and update of the open-access database of TF binding profiles. Nucleic Acids Res..

[332] Kulakovskiy I.V., Vorontsov I.E., Yevshin I.S., Sharipov R.N., Fedorova A.D., Rumynskiy E.I., Medvedeva Y.A., Magana-Mora A., Bajic V.B., Papatsenko D.A. (2018). HOCOMOCO: Towards a complete collection of TF binding models for human and mouse via large-scale ChIP-Seq analysis. Nucleic Acids Res..

[333] Khan A., Fornes O., Stigliani A., Gheorghe M., Castro-Mondragon J.A., van der Lee R., Bessy A., Cheneby J., Kulkarni S.R., Tan G. (2018). JASPAR 2018: Update of the open-access data-base of transcription factor binding profiles and its web framework. Nucleic Acids Res..

[334] Castro-Mondragon J.A., Riudavets-Puig R., Rauluseviciute I., Berhanu Lemma R., Turchi L., Blanc-Mathieu R., Lucas J., Boddie P., Khan A., Manosalva Perez N. (2022). JASPAR 2022: The 9th release of the open-access database of TF binding profiles. Nucleic Acids Res..

[335] Desplan C., Theis J., O’Farrell P.H. (1985). The Drosophila developmental gene, engrailed, encodes a sequence-specific DNA binding activity. Nature.

[336] Treisman J., Gonczy P., Vashishtha M., Harris E., Desplan C. (1989). A single amino acid can determine the DNA binding specificity of homeodomain proteins. Cell.

[337] Vermunt M.W., Tan S.C., Castelijns B., Geeven G., Reinink P., de Bruijn E., Kondova I., Persengiev S., Netherlands Brain B., Bontrop R. (2016). Epigenomic annotation of gene regulatory alterations during evolution of the primate brain. Nat. Neurosci..

[338] Fish A., Chen L., Capra J.A. (2017). Gene Regulatory Enhancers with Evolutionarily Conserved Activity Are More Pleiotropic than Those with Species-Specific Activity. Genome Biol. Evol..

[339] Maurano M.T., Humbert R., Rynes E., Thurman R.E., Haugen E., Wang H., Reynolds A.P., Sandstrom R., Qu H., Brody J. (2012). Systematic localization of common disease-associated variation in regulatory DNA. Science.

[340] Leslie R., O’Donnell C.J., Johnson A.D. (2014). GRASP: Analysis of genotype-phenotype results from 1390 genome-wide association studies and corresponding open access database. Bioinformatics.

[341] Soldner F., Stelzer Y., Shivalila C.S., Abraham B.J., Latourelle J.C., Barrasa M.I., Goldmann J., Myers R.H., Young R.A., Jaenisch R. (2016). Parkinson-associated risk variant in distal enhancer of alpha-synuclein modulates target gene expression. Nature.

[342] Castelijns B., Baak M.L., Geeven G., Vermunt M.W., Wiggers C.R.M., Timpanaro I.S., Kondova I., de Laat W., Creyghton M.P. (2020). Recently Evolved Enhancers Emerge with High Interindividual Variability and Less Frequently Associate with Disease. Cell Rep..

[343] Villar D., Flicek P., Odom D.T. (2014). Evolution of TF binding in metazoans—Mechanisms and functional implications. Nat. Rev. Genet..

[344] Villar D., Berthelot C., Aldridge S., Rayner T.F., Lukk M., Pignatelli M., Park T.J., Deaville R., Erichsen J.T., Jasinska A.J. (2015). Enhancer evolution across 20 mammalian species. Cell.

[345] Pennacchio L.A., Bickmore W., Dean A., Nobrega M.A., Bejerano G. (2013). Enhancers: Five essential questions. Nat. Rev. Genet..

[346] Osterwalder M., Barozzi I., Tissieres V., Fukuda-Yuzawa Y., Mannion B.J., Afzal S.Y., Lee E.A., Zhu Y., Plajzer-Frick I., Pickle C.S. (2018). Enhancer redundancy provides phenotypic robustness in mammalian development. Nature.

[347] Carelli F.N., Liechti A., Halbert J., Warnefors M., Kaessmann H. (2018). Repurposing of promoters and enhancers during mammalian evolution. Nat. Commun..

[348] Gupta S., Stamatoyannopoulos J.A., Bailey T.L., Noble W.S. (2007). Quantifying similarity between motifs. Genome Biol..

[349] Castro-Mondragon J.A., Jaeger S., Thieffry D., Thomas-Chollier M., van Helden J. (2017). RSAT matrix-clustering: Dynamic exploration and redundancy reduction of TF binding motif collections. Nucleic Acids Res..

[350] La Manno G., Soldatov R., Zeisel A., Braun E., Hochgerner H., Petukhov V., Lidschreiber K., Kastriti M.E., Lonnerberg P., Furlan A. (2018). RNA velocity of single cells. Nature.

[351] Bergen V., Lange M., Peidli S., Wolf F.A., Theis F.J. (2020). Generalizing RNA velocity to transient cell states through dynamical modeling. Nat. Biotechnol..

[352] Tedesco M., Giannese F., Lazarevic D., Giansanti V., Rosano D., Monzani S., Catalano I., Grassi E., Zanella E.R., Botrugno O.A. (2021). Chromatin Velocity reveals epigenetic dynamics by single-cell profiling of heterochromatin and euchromatin. Nat. Biotechnol..

[353] Worley B., Powers R. (2015). A Sequential Algorithm for Multiblock Orthogonal Projections to Latent Structures. Chemom. Intell. Lab. Syst..

[354] Zimmerman K.D., Espeland M.A., Langefeld C.D. (2021). A practical solution to pseudoreplication bias in single-cell studies. Nat. Commun..

[355] Liu Q., Sheng Q., Ping J., Ramirez M.A., Lau K.S., Coffey R.J., Shyr Y. (2019). scRNABatchQC: Multi-samples quality control for single cell RNA-seq data. Bioinformatics.

[356] Hashimshony T., Senderovich N., Avital G., Klochendler A., de Leeuw Y., Anavy L., Gennert D., Li S., Livak K.J., Rozenblatt-Rosen O. (2016). CEL-Seq2: Sensitive highly-multiplexed single-cell RNA-Seq. Genome Biol..

[357] Finak G., McDavid A., Yajima M., Deng J., Gersuk V., Shalek A.K., Slichter C.K., Miller H.W., McElrath M.J., Prlic M. (2015). MAST: A flexible statistical framework for assessing transcriptional changes and characterizing heterogeneity in single-cell RNA sequencing data. Genome Biol..

[358] Raj A., van Oudenaarden A. (2008). Nature, nurture, or chance: Stochastic gene expression and its consequences. Cell.

[359] Van den Berge K., Perraudeau F., Soneson C., Love M.I., Risso D., Vert J.P., Robinson M.D., Dudoit S., Clement L. (2018). Observation weights unlock bulk RNA-seq tools for zero inflation and single-cell applications. Genome Biol..

[360] Pierson E., Yau C. (2015). ZIFA: Dimensionality reduction for zero-inflated single-cell gene expression analysis. Genome Biol..

[361] Lin P., Troup M., Ho J.W. (2017). CIDR: Ultrafast and accurate clustering through imputation for single-cell RNA-seq data. Genome Biol..

[362] Svensson V. (2020). Droplet scRNA-seq is not zero-inflated. Nat. Biotechnol..

[363] Lun A.T., Bach K., Marioni J.C. (2016). Pooling across cells to normalize single-cell RNA sequencing data with many zero counts. Genome Biol..

[364] Lun A.T.L., Marioni J.C. (2017). Overcoming confounding plate effects in differential expression analyses of single-cell RNA-seq data. Biostatistics.

[365] Crowell H.L., Soneson C., Germain P.L., Calini D., Collin L., Raposo C., Malhotra D., Robinson M.D. (2020). muscat detects subpopulation-specific state transitions from multi-sample multi-condition single-cell transcriptomics data. Nat. Commun..

[366] Cusanovich D.A., Hill A.J., Aghamirzaie D., Daza R.M., Pliner H.A., Berletch J.B., Filippova G.N., Huang X., Christiansen L., DeWitt W.S. (2018). A Single-Cell Atlas of In Vivo Mammalian Chromatin Accessibility. Cell.

[367] Swanson E., Lord C., Reading J., Heubeck A.T., Genge P.C., Thomson Z., Weiss M.D., Li X.J., Savage A.K., Green R.R. (2021). Simultaneous trimodal single-cell measurement of transcripts, epitopes, and chromatin accessibility using TEA-seq. eLife.

[368] Fang R., Preissl S., Li Y., Hou X., Lucero J., Wang X., Motamedi A., Shiau A.K., Zhou X., Xie F. (2021). Comprehensive analysis of single cell ATAC-seq data with SnapATAC. Nat. Commun..

[369] Kiselev V.Y., Andrews T.S., Hemberg M. (2019). Challenges in unsupervised clustering of single-cell RNA-seq data. Nat. Rev. Genet..

[370] McInnes L., Healy J., Melville J. (2018). Umap: Uniform manifold approximation and projection for dimension reduction. arXiv.

[371] Becht E., McInnes L., Healy J., Dutertre C.A., Kwok I.W.H., Ng L.G., Ginhoux F., Newell E.W. (2018). Dimensionality reduction for visualizing single-cell data using UMAP. Nat. Biotechnol..

[372] Chen H., Lareau C., Andreani T., Vinyard M.E., Garcia S.P., Clement K., Andrade-Navarro M.A., Buenrostro J.D., Pinello L. (2019). Assessment of computational methods for the analysis of single-cell ATAC-seq data. Genome Biol..

[373] Xiang R., Wang W., Yang L., Wang S., Xu C., Chen X. (2021). A Comparison for Dimensionality Reduction Methods of Single-Cell RNA-seq Data. Front. Genet..

[374] Linderman G.C. (2021). Dimensionality Reduction of Single-Cell RNA-Seq Data. Methods Mol. Biol..

[375] Ma K.Y., Schonnesen A.A., Brock A., Van Den Berg C., Eckhardt S.G., Liu Z., Jiang N. (2019). Single-cell RNA sequencing of lung adenocarcinoma reveals heterogeneity of immune response-related genes. JCI Insight.

[376] Mendoza-Parra M.A., Van Gool W., Mohamed Saleem M.A., Ceschin D.G., Gronemeyer H. (2013). A quality control system for profiles obtained by ChIP sequencing. Nucleic Acids Res..

[377] Li Q., Brown J.B., Huang H., Bickel P.J. (2011). Measuring reproducibility of high-throughput experiments. Ann. Appl. Stat..

[378] Thanawala S.U., Rister J., Goldberg G.W., Zuskov A., Olesnicky E.C., Flowers J.M., Jukam D., Purugganan M.D., Gavis E.R., Desplan C. (2013). Regional modulation of a stochastically expressed factor determines photoreceptor subtypes in the Drosophila retina. Dev. Cell.

[379] Martin-Gayo E., Cole M.B., Kolb K.E., Ouyang Z., Cronin J., Kazer S.W., Ordovas-Montanes J., Lichterfeld M., Walker B.D., Yosef N. (2018). A Reproducibility-Based Computational Framework Identifies an Inducible, Enhanced Antiviral State in Dendritic Cells from HIV-1 Elite Controllers. Genome Biol..

[380] Karabacak Calviello A., Hirsekorn A., Wurmus R., Yusuf D., Ohler U. (2019). Reproducible inference of TF footprints in ATAC-seq and DNase-seq datasets using protocol-specific bias modeling. Genome Biol..

[381] Zhang X., Zhang Y., Zhu X., Purmann C., Haney M.S., Ward T., Khechaduri A., Yao J., Weissman S.M., Urban A.E. (2018). Local and global chromatin interactions are altered by large genomic deletions associated with human brain development. Nat. Commun..

[382] Spielmann M., Lupianez D.G., Mundlos S. (2018). Structural variation in the 3D genome. Nat. Rev. Genet..

[383] Zheng H., Xie W. (2019). The role of 3D genome organization in development and cell differentiation. Nat. Rev. Mol. Cell Biol..

[384] Mateo L.J., Murphy S.E., Hafner A., Cinquini I.S., Walker C.A., Boettiger A.N. (2019). Visualizing DNA folding and RNA in embryos at single-cell resolution. Nature.

[385] Takei Y., Shah S., Harvey S., Qi L.S., Cai L. (2017). Multiplexed Dynamic Imaging of Genomic Loci by Combined CRISPR Imaging and DNA Sequential FISH. Biophys. J..

[386] Boettiger A., Murphy S. (2020). Advances in Chromatin Imaging at Kilobase-Scale Resolution. Trends Genet..

[387] Takei Y., Zheng S., Yun J., Shah S., Pierson N., White J., Schindler S., Tischbirek C.H., Yuan G.C., Cai L. (2021). Single-cell nuclear architecture across cell types in the mouse brain. Science.

[388] Betzig E., Patterson G.H., Sougrat R., Lindwasser O.W., Olenych S., Bonifacino J.S., Davidson M.W., Lippincott-Schwartz J., Hess H.F. (2006). Imaging intracellular fluorescent proteins at nanometer resolution. Science.

[389] Beliveau B.J., Boettiger A.N., Nir G., Bintu B., Yin P., Zhuang X., Wu C.T. (2017). In Situ Super-Resolution Imaging of Genomic DNA with OligoSTORM and OligoDNA-PAINT. Methods Mol. Biol..

[390] Beliveau B.J., Joyce E.F., Apostolopoulos N., Yilmaz F., Fonseka C.Y., McCole R.B., Chang Y., Li J.B., Senaratne T.N., Williams B.R. (2012). Versatile design and synthesis platform for visualizing genomes with Oligopaint FISH probes. Proc. Natl. Acad. Sci. USA.

[391] Jungmann R., Avendano M.S., Woehrstein J.B., Dai M., Shih W.M., Yin P. (2014). Multiplexed 3D cellular super-resolution imaging with DNA-PAINT and Exchange-PAINT. Nat. Methods.

[392] Rust M.J., Bates M., Zhuang X. (2006). Sub-diffraction-limit imaging by stochastic optical reconstruction microscopy (STORM). Nat. Methods.

[393] Huang B., Wang W., Bates M., Zhuang X. (2008). Three-dimensional super-resolution imaging by stochastic optical reconstruction microscopy. Science.

[394] Yu M., Ren B. (2017). The Three-Dimensional Organization of Mammalian Genomes. Annu. Rev. Cell Dev. Biol..

[395] Cavalli G., Heard E. (2019). Advances in epigenetics link genetics to the environment and disease. Nature.

[396] Bintu B., Mateo L.J., Su J.H., Sinnott-Armstrong N.A., Parker M., Kinrot S., Yamaya K., Boettiger A.N., Zhuang X. (2018). Super-resolution chromatin tracing reveals domains and cooperative interactions in single cells. Science.

[397] Barth R., Fourel G., Shaban H.A. (2020). Dynamics as a cause for the nanoscale organization of the genome. Nucleus.

[398] Su J.H., Zheng P., Kinrot S.S., Bintu B., Zhuang X. (2020). Genome-Scale Imaging of the 3D Organization and Transcriptional Activity of Chromatin. Cell.

[399] Liu Y., Yang M., Deng Y., Su G., Enninful A., Guo C.C., Tebaldi T., Zhang D., Kim D., Bai Z. (2020). High-Spatial-Resolution Multi-Omics Sequencing via Deterministic Barcoding in Tissue. Cell.

[400] Stevens T.J., Lando D., Basu S., Atkinson L.P., Cao Y., Lee S.F., Leeb M., Wohlfahrt K.J., Boucher W., O’Shaughnessy-Kirwan A. (2017). 3D structures of individual mammalian genomes studied by single-cell Hi-C. Nature.

[401] Nagano T., Lubling Y., Varnai C., Dudley C., Leung W., Baran Y., Mendelson Cohen N., Wingett S., Fraser P., Tanay A. (2017). Cell-cycle dynamics of chromosomal organization at single-cell resolution. Nature.

[402] Flyamer I.M., Gassler J., Imakaev M., Brandao H.B., Ulianov S.V., Abdennur N., Razin S.V., Mirny L.A., Tachibana-Konwalski K. (2017). Single-nucleus Hi-C reveals unique chromatin reorganization at oocyte-to-zygote transition. Nature.

[403] Tan L., Xing D., Chang C.H., Li H., Xie X.S. (2018). Three-dimensional genome structures of single diploid human cells. Science.

[404] Ou H.D., Phan S., Deerinck T.J., Thor A., Ellisman M.H., O’Shea C.C. (2017). ChromEMT: Visualizing 3D chromatin structure and compaction in interphase and mitotic cells. Science.

[405] Wang H.V., Corces V.G. (2019). Seeing Is Believing: ORCA Allows Visualization of Three-Dimensional Genome Organization at Single-Cell Resolution. Biochemistry.

[406] Wang S., Su J.H., Beliveau B.J., Bintu B., Moffitt J.R., Wu C.T., Zhuang X. (2016). Spatial organization of chromatin domains and compartments in single chromosomes. Science.

[407] Nir G., Farabella I., Perez Estrada C., Ebeling C.G., Beliveau B.J., Sasaki H.M., Lee S.D., Nguyen S.C., McCole R.B., Chattoraj S. (2018). Walking along chromosomes with super-resolution imaging, contact maps, and integrative modeling. PLoS Genet..

[408] Boettiger A.N., Bintu B., Moffitt J.R., Wang S., Beliveau B.J., Fudenberg G., Imakaev M., Mirny L.A., Wu C.T., Zhuang X. (2016). Super-resolution imaging reveals distinct chromatin folding for different epigenetic states. Nature.

[409] Kundu S., Ji F., Sunwoo H., Jain G., Lee J.T., Sadreyev R.I., Dekker J., Kingston R.E. (2017). Polycomb Repressive Complex 1 Generates Discrete Compacted Domains that Change during Differentiation. Mol. Cell.

[410] Tasic B., Menon V., Nguyen T.N., Kim T.K., Jarsky T., Yao Z., Levi B., Gray L.T., Sorensen S.A., Dolbeare T. (2016). Adult mouse cortical cell taxonomy revealed by single cell transcriptomics. Nat. Neurosci..

[411] Payne A.C., Chiang Z.D., Reginato P.L., Mangiameli S.M., Murray E.M., Yao C.C., Markoulaki S., Earl A.S., Labade A.S., Jaenisch R. (2021). In situ genome sequencing resolves DNA sequence and structure in intact biological samples. Science.

[412] Cao B., Coelho S., Li J., Wang G., Pertsinidis A. (2021). Volumetric interferometric lattice light-sheet imaging. Nat. Biotechnol..

[413] Li Y., Eshein A., Virk R.K.A., Eid A., Wu W., Frederick J., VanDerway D., Gladstein S., Huang K., Shim A.R. (2021). Nanoscale chromatin imaging and analysis platform bridges 4D chromatin organization with molecular function. Sci. Adv..

[414] Patel A.P., Tirosh I., Trombetta J.J., Shalek A.K., Gillespie S.M., Wakimoto H., Cahill D.P., Nahed B.V., Curry W.T., Martuza R.L. (2014). Single-cell RNA-seq highlights intratumoral heterogeneity in primary glioblastoma. Science.

[415] Usoskin D., Furlan A., Islam S., Abdo H., Lonnerberg P., Lou D., Hjerling-Leffler J., Haeggstrom J., Kharchenko O., Kharchenko P.V. (2015). Unbiased classification of sensory neuron types by large-scale single-cell RNA sequencing. Nat. Neurosci..

[416] Buettner F., Natarajan K.N., Casale F.P., Proserpio V., Scialdone A., Theis F.J., Teichmann S.A., Marioni J.C., Stegle O. (2015). Computational analysis of cell-to-cell heterogeneity in single-cell RNA-sequencing data reveals hidden subpopulations of cells. Nat. Biotechnol..

[417] Kolodziejczyk A.A., Kim J.K., Tsang J.C., Ilicic T., Henriksson J., Natarajan K.N., Tuck A.C., Gao X., Buhler M., Liu P. (2015). Single Cell RNA-Sequencing of Pluripotent States Unlocks Modular Transcriptional Variation. Cell Stem Cell.

[418] Nowakowski T.J., Bhaduri A., Pollen A.A., Alvarado B., Mostajo-Radji M.A., Di Lullo E., Haeussler M., Sandoval-Espinosa C., Liu S.J., Velmeshev D. (2017). Spatiotemporal gene expression trajectories reveal developmental hierarchies of the human cortex. Science.

[419] Li L., Dong J., Yan L., Yong J., Liu X., Hu Y., Fan X., Wu X., Guo H., Wang X. (2017). Single-Cell RNA-Seq Analysis Maps Development of Human Germline Cells and Gonadal Niche Interactions. Cell Stem Cell.

[420] Mayer C., Hafemeister C., Bandler R.C., Machold R., Batista Brito R., Jaglin X., Allaway K., Butler A., Fishell G., Satija R. (2018). Developmental diversification of cortical inhibitory interneurons. Nature.

[421] Zhong S., Zhang S., Fan X., Wu Q., Yan L., Dong J., Zhang H., Li L., Sun L., Pan N. (2018). A single-cell RNA-seq survey of the developmental landscape of the human prefrontal cortex. Nature.

[422] Polioudakis D., de la Torre-Ubieta L., Langerman J., Elkins A.G., Shi X., Stein J.L., Vuong C.K., Nichterwitz S., Gevorgian M., Opland C.K. (2019). A Single-Cell Transcriptomic Atlas of Human Neocortical Development during Mid-gestation. Neuron.

[423] Setty M., Kiseliovas V., Levine J., Gayoso A., Mazutis L., Pe’er D. (2019). Characterization of cell fate probabilities in single-cell data with Palantir. Nat. Biotechnol..

[424] Zhang K., Hocker J.D., Miller M., Hou X., Chiou J., Poirion O.B., Qiu Y., Li Y.E., Gaulton K.J., Wang A. (2021). A single-cell atlas of chromatin accessibility in the human genome. Cell.

[425] Zibetti C., Liu S., Wan J., Qian J., Blackshaw S. (2017). Lhx2 regulates temporal changes in chromatin accessibility and transcription factor binding in retinal progenitor cells. bioRxiv.

[426] Steiner F.A., Talbert P.B., Kasinathan S., Deal R.B., Henikoff S. (2012). Cell-type-specific nuclei purification from whole animals for genome-wide expression and chromatin profiling. Genome Res..

[427] Schreiber J., Bilmes J., Noble W.S. (2021). Prioritizing transcriptomic and epigenomic experiments using an optimization strategy that leverages imputed data. Bioinformatics.

[428] Wahls W.P. (2018). The NIH must reduce disparities in funding to maximize its return on investments from taxpayers. eLife.

[429] Barnett A.G., Clarke P., Vaquette C., Graves N. (2017). Using democracy to award research funding: An observational study. Res. Integr. Peer Rev..

[430] Gasparyan A.Y., Yessirkepov M., Voronov A.A., Gerasimov A.N., Kostyukova E.I., Kitas G.D. (2015). Preserving the Integrity of Citations and References by All Stakeholders of Science Communication. J. Korean Med. Sci..

[431] Dworkin J.D., Linn K.A., Teich E.G., Zurn P., Shinohara R.T., Bassett D.S. (2020). The extent and drivers of gender imbalance in neuroscience reference lists. Nat. Neurosci..

[432] Zurn P., Bassett D.S., Rust N.C. (2020). The Citation Diversity Statement: A Practice of Transparency, A Way of Life. Trends Cogn. Sci..

[433] Scanff A., Naudet F., Cristea I.A., Moher D., Bishop D.V.M., Locher C. (2021). A survey of biomedical journals to detect editorial bias and nepotistic behavior. PLoS Biol..

[434] Tiokhin L., Panchanathan K., Lakens D., Vazire S., Morgan T., Zollman K. (2021). Honest signaling in academic publishing. PLoS ONE.

[435] McConnell S.C., Westerman E.L., Pierre J.F., Heckler E.J., Schwartz N.B. (2018). United States National Postdoc Survey results and the interaction of gender, career choice and mentor impact. eLife.

[436] Davis S.M., Singh H., Weismann C.M., Bankston A., Ruiz Villalobos J.P. (2020). Actionable recommendations from trainees to improve science training. eLife.

[437] Byars-Winston A., Dahlberg M.L., National Academies of Sciences, Engineering, and Medicine (2019). The Science of Effective Mentorship in STEMM..

[438] Barres B.A. (2017). Stop blocking postdocs’ paths to success. Nature.

[439] Kaptein M. (2008). Developing and testing a measure for the ethical culture of organizations: The corporate ethical virtues model. J. Organ. Behav..

